# Cotton pan-genome retrieves the lost sequences and genes during domestication and selection

**DOI:** 10.1186/s13059-021-02351-w

**Published:** 2021-04-23

**Authors:** Jianying Li, Daojun Yuan, Pengcheng Wang, Qiongqiong Wang, Mengling Sun, Zhenping Liu, Huan Si, Zhongping Xu, Yizan Ma, Boyang Zhang, Liuling Pei, Lili Tu, Longfu Zhu, Ling-Ling Chen, Keith Lindsey, Xianlong Zhang, Shuangxia Jin, Maojun Wang

**Affiliations:** 1 National Key Laboratory of Crop Genetic Improvement, Huazhong Agricultural University, Wuhan, China; 2 College of Plant Science and Technology, Huazhong Agricultural University, Wuhan, China; 3 Hubei Key Laboratory of Agricultural Bioinformatics, College of Informatics, Huazhong Agricultural University, Wuhan, China; 4 Department of Biosciences, Durham University, Durham, UK

**Keywords:** Cotton, Domestication, Improvement, Pan-genome, Copy number variation (CNV), Presence/absence variation (PAV), Gene loss

## Abstract

**Background:**

Millennia of directional human selection has reshaped the genomic architecture of cultivated cotton relative to wild counterparts, but we have limited understanding of the selective retention and fractionation of genomic components.

**Results:**

We construct a comprehensive genomic variome based on 1961 cottons and identify 456 Mb and 357 Mb of sequence with domestication and improvement selection signals and 162 loci, 84 of which are novel, including 47 loci associated with 16 agronomic traits. Using pan-genome analyses, we identify 32,569 and 8851 non-reference genes lost from *Gossypium hirsutum* and *Gossypium barbadense* reference genomes respectively, of which 38.2% (39,278) and 14.2% (11,359) of genes exhibit presence/absence variation (PAV). We document the landscape of PAV selection accompanied by asymmetric gene gain and loss and identify 124 PAVs linked to favorable fiber quality and yield loci.

**Conclusions:**

This variation repertoire points to genomic divergence during cotton domestication and improvement, which informs the characterization of favorable gene alleles for improved breeding practice using a pan-genome-based approach.

## Background

Cotton is cultivated worldwide for its fiber and seed oil. Allotetraploid cultivated cotton (*Gossypium hirsutum* and *Gossypium barbadense*) originated from interspecies hybridization between putative diploid A genome (*Gossypium herbaceum* or *Gossypium arboretum*) and D genome (*Gossypium raimondii*) ancestors approximately 1–1.5 million years ago (MYA) [1, 2]. *G. hirsutum* was initially domesticated from wild cotton in the Yucatan peninsula and subsequently developed seven semi-wild forms, which were subject to directional domestication selection to form the American cultivated cotton with high yield and spinnable fine white fibers [3–6]. DNA-based molecular markers have revealed admixture population structure and high genetic diversity of *G. hirsutum* wild, landrace, and American cultivated cotton [7–10]. Founder cultivars of *G. hirsutum* in America have been introduced widely to other countries and improved modern cultivars in China show wide phenotypic variation and adaptation [3–6]. *G. barbadense* is native to the coastal areas of Peru and is cultivated in a limited number of areas with superior fibers [11]. The history of allotetraploid cotton domestication and selection has been revealed at small-scale variation level in specific population, but the understanding of entire genomic variome remains fragmentary.

Genome assemblies of *G. hirsutum* and *G. barbadense* cultivars have identified extensive variation between the species [12, 13]. Hundreds of diverse cotton accessions have been sequenced, providing an opportunity to construct a multi-dimensional variation genome (variome) to reveal genome divergence during domestication and identify loci underlying improvement traits [4–6, 14–16]. However, these genomic variations were identified by sequence reads mapped to a reference genome, giving an incomplete picture, especially for the lack of presence/absence variation (PAV) and copy number variation (CNV). To comprehensively capture the genetic variation missed by using one reference, the construction of a “pan-genome,” a collection of all the DNA sequences from all individuals in a species, has a great value [17]. Pan-genomic studies can identify PAVs between wild and cultivated accessions for a better understanding of crop domestication [18–31]. In tomato, 351 Mb non-reference sequences with 4873 novel genes, including 74% for core genes (present in all accessions) and 26% for dispensable genes (present in at least one accession), were assembled using 725 representative wild and improved accessions [26]. In soybean, based on long reads of 27 wild and cultivated soybean accessions, pan-genome assembly revealed that 36% and 64% of genes were core and dispensable respectively, some of which were associated with domestication traits [30]. These studies suggest that PAVs are widespread and play an important role in genetic determination of phenotypic variation [32], to reveal favorable genotypes for crop improvement.

Here we analyze genomic variation among 1961 cottons, revealing extensive genomic diversity, including 63 million single-nucleotide polymorphisms (SNPs), 4.9 million small insertion/deletions (InDels), and over 290,000 structural variations (SVs). We constructed pan-genomes of *G. hirsutum* and *G. barbadense*, which include 1041 Mb (32,569 genes) and 309 Mb (8851 genes) non-reference sequences, respectively. The domestication and improvement process has led to asymmetric gene gain and loss, which shaped the genomic architecture of cultivated cotton. The pan-genome data inform us to understand how domestication and improvement has driven genomic picture underlying the desirable agronomic traits for further cotton breeding.

## Results

### Genetic diversity and population properties

**Fig. 1:**
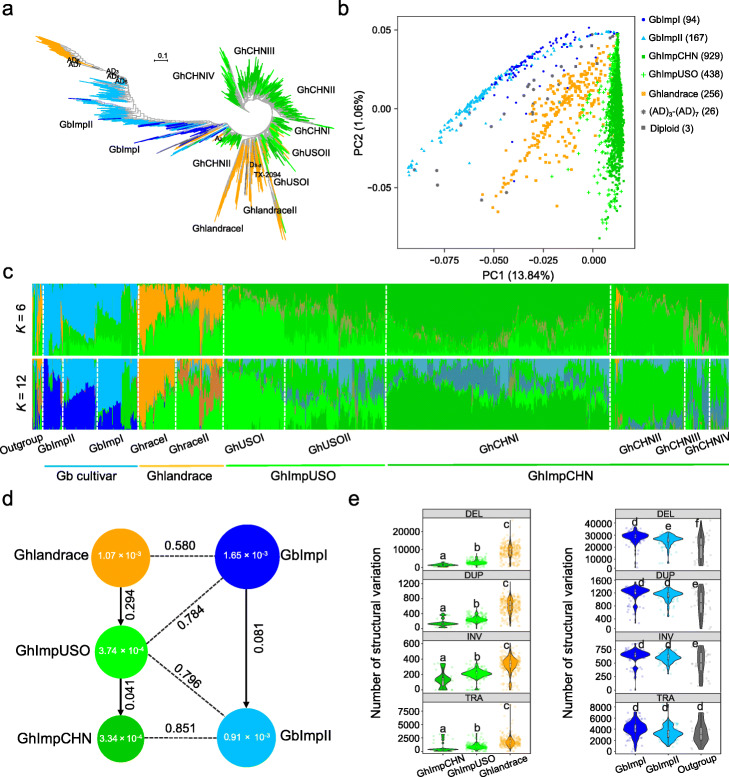
Population structure and genetic diversity in *G. hirsutum* and *G. barbadense* accessions. **a** The unweighted neighbor-joining phylogenetic tree of 1913 cotton accessions was constructed based on 20,000 random SNPs from core SNPs. The *G. tomentosum* (AD_3_), *G. mustelinum* (AD_4_), *G. darwinii* (AD_5_), *G. ekmanianum* (AD_6_), *G. stephensii* (AD_7_) of tetraploid species, *G. arboreum* (A_2_) and *G. davidsonii* (D_3-d_) of diploid species serve as outgroup. **b** Principal component analysis (PCA) plot of the first two components for all accessions. **c** STRUCTURE analysis of all cotton accessions with different numbers of clusters *K* = 6 and *K* = 12 (*K* = 12 is optimal value). The *x*-axis lists the outgroup species (gray), *G. barbadense* (blue), *G. hirsutum* landrace accessions (orange), and *G. hirsutum* improved accessions (green) respectively, and the *y*-axis quantifies genetic diversity in each accession. The other structure results are shown in the Additional file 2: Figure S2. **d** Nucleotide diversity (π) and fixation index divergence (*F*st) across the five groups. **e** The number of deletions, duplications, inversions, and translocations in five populations (two-sided Wilcoxon rank-sum test for adjacent groups, *P* < 0.001). Each node represents one accession. In this analysis, the number of SVs was shown with the TM-1 reference genome

We collected DNA re-sequencing data for 1961 cottons for a genomic variation analysis with an average depth of ~ 14.8× for each [3–6, 16, 33, 34]. After discarding duplicated accessions, a total of 1913 cotton accessions were used for SNP and InDel analysis, which included 256 *G. hirsutum* landraces (Ghlandraces), 438 improved *G. hirsutum* cultivars from the USA and other countries (GhImpUSO), 929 improved *G. hirsutum* cultivars from China (GhImpCHN), 261 *G. barbadense* accessions, and 29 other *Gossypium* species that were used as outgroup (Additional file 1: Table S1). We aligned these data against the reference genome of *G. hirsutum* acc. “TM-1” [12] and identified 63,084,975 SNPs and 12,354,432 small insertions or deletions (InDels length ≤ 20 bp), in which the core variation dataset includes 19,246,497 SNPs and 4,815,125 InDels with a minor allele frequency (MAF) ≥ 0.01 and more than five accessions having homozygous variations (Table 1; Additional file 1: Tables S2-S6; Additional file 3). Based on core SNP data, we investigated the population structure of *G. hirsutum* and *G. barbadense*. Neighbor-joining tree analysis showed the 1913 accessions classify into 12 clades. *G. hirsutum* accessions form 8 clades, *G. barbadense* accessions form 3 clades, and other species form 1 clade (Fig. 1a; Additional file 2: Figure S1). Population analysis showed that *G. barbadense* accessions were separated from the *G. hirsutum* landraces, GhImpUSO and GhImpCHN (Fig. 1b, c; Additional file 2: Figure S2). *G. hirsutum* nucleotide diversity (*π*) is estimated at 1.07 × 10^− 3^ in landraces, 3.74 × 10^− 4^ in GhImpUSO, 3.34 × 10^− 4^ in GhImpCHN, and 1.01 × 10^− 3^ in *G. barbadense* (Additional file 2: Figure S3), similar to the recent studies in cotton [3–6, 34] (Fig. 1d).

**Table 1:** Genome-wide genomic variations in a large cotton population

Variation type	Total (1913)	Gh cultivar (1623)	Ghlandrace (256)	GhImpUSO (438)	GhImpCHN (929)	Gb cultivar (261)	AD_3_-AD_7_ (26)
Bi-allele SNP^a^	19,246,497	9,546,748	9,265,438	4766,399	3,761,448	19,473,033	32,878,758
Splicing	2172	1213	1149	652	554	2041	11,366
Exonic	315,404	179,665	172,718	103,126	89,208	316,146	776,644
Intronic	607,301	335,212	322,141	189,798	152,656	575,524	1,010,509
UTR	220,664	120,198	116,269	65,226	52,342	197,420	390,008
Upstream	869,678	448,709	432,640	238,937	169,788	789,898	984,811
Downstream	797,469	413,937	399,140	222,266	161,602	729,445	959,584
Nonsynonymous	195,883	111,686	107,143	63,008	52,853	177,474	420,190
InDel (≤ 20 bp) ^a^	4,815,125	3,971,277	3,744,299	1,672,195	1,726,445	3,366,481	7,625,077
Splicing	1202	1128	941	570	735	1104	2465
Exonic	31,661	27,238	28,815	12,807	14,826	26,455	65,677
Intronic	262,657	231,561	215,663	95,674	94,830	183,387	539,379
UTR	113,824	100,811	96,003	36,418	37,175	76,684	261,351
Upstream	578,086	497,660	413,192	201,122	226,848	400,965	927,134
Downstream	429,514	369,517	311,164	148,050	166,059	309,829	717,980
Frameshift	23,330	20,367	22,040	9798	11,029	19,603	42,328
SV (> 50 bp)	214,310	104,523	97,933	64,064	61,616	132,499	281,476
Deletion^b^	32,099	22,340	9933	7029	23,559	13,982	15,484
Duplication^b^	7576	5146	4766	1721	NA	3252	3718
Inversion^b^	1112	724	615	310	NA	877	613
Translocation^b^	357	240	188	167	NA	504	412
CNV^c^	173,166	76,073	82,431	54,837	38,057	99,274	261,249

^a^The 261 *G. barbadense* accessions were aligned to the “TM-1” reference genome. The *G. barbadense* population SNP and InDel calling results against the “3–79” reference genome are shown in Additional file 1: Table S5. ^b^Genotyping structural variations (SVs) in 742 cottons. The *G. hirsutum* TM-1 reference genome was used for detecting variations. The number of genotypes in each group is in parentheses. “NA” represents the missing combined SVs. DUP, INV, and TRA were not included for the GhImpCHN population. ^c^CNVs were identified in 742 cottons. Only variation in each chromosome was counted and further analyzed

We used 742 cotton accessions with a high sequencing depth (> 10×) against the *G. hirsutum* “TM-1” reference genome (Additional file 1: Table S1; Additional file 3) and identified 32,099 deletions, 7576 duplications, 1112 inversions, and 357 translocations (Additional file 1: Table S7). There are more SVs in Ghlandrace than that GhImpUSO and GhImpCHN groups (Fig. 1e). In addition, 173,166 (MAF ≥ 0.01) copy number variations (CNVs) were identified in the 742 accessions, including 82,431 in the landraces, 59,309 in the GhImpUSO, and 38,057 in the GhImpCHN group (Additional file 1: Table S8). Population genetic properties of CNVs in 742 accessions showed that *G. hirsutum* landraces were clearly separated from the improved accessions, similar to SNP-based result, but were clustered together with the GhImpUSO and GhImpCHN accessions (Additional file 2: Figure S4). These results suggested that high-confidence CNVs have strong divergence between *G. hirsutum* landrace and improved population and can be used to discover complex quantitative trait loci (QTLs). This comprehensive variome dataset provides a genomic resource for cotton population genetics, domestication analysis, and agronomic allele identification (Additional file 2: Figure S5).

### Evidence for genomic divergence during domestication and improvement

**Fig. 2:**
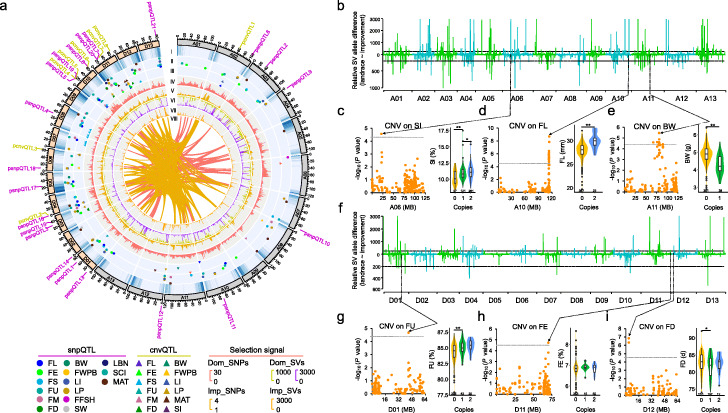
Multiple-scale variation for subgenomic divergence and GWAS on agronomic traits during cotton domestication. **a** Circos plot showing the SNP- and SV-based selection signals and QTLs during cotton domestication and improvement. The selection region was calculated in a 1-Mb sliding window with a step size of 200 kb. I–VIII, Circos plot from outer to inter tracks showing gene density (I), snpQTLs (II), cnvQTLs (III), the ratio of nucleotide diversity (π) based on SNPs between 256 landraces and 1364 improved accessions for domestication (IV), the ratio of nucleotide diversity (π) based on SNPs between 438 GhImpUSO accessions and 929 GhImpCHN accessions for improvement (V), the relative SV allele difference in the comparisons between landrace and improved accessions (VI), and between GhImpUSO and GhImpCHN (VII). The track (VIII) represents the domesticated homologous. Upper and lower panels (VI) represent deletion and duplication variation allele difference, respectively. The snpQTLs were identified using the meta-GWAS analysis of 890 cotton accessions. The outermost circle of the circos plot purple and yellow font shows pleiotropic snpQTLs (psnpQTLs) and pleiotropic cnvQTLs (pcnvQTLs), respectively. **b–i** Selective signals of copy number variations (CNVs) between the A (**b**) and D (**f**) subgenome during domestication. The horizontal gray dashed lines show the domestication signal threshold with the ratio of nucleotide diversity between wild/landrace and improved cotton accessions (π_landrace_/π_Improved_ > 200). **c–e** and **g–i** Six CNV-based GWAS hits that overlapped with domestication selection signals are shown for seed index (SI) (**c**), fiber length (FL) (**d**), boll weight (BW) (**e**), fiber uniformity (FU) (**g**), fiber elongation (FE) (**h**), and flowering date (FD) (**i**). The threshold of cnvQTL line was -log_10_ *P =* 4.4. The violin plot showed phenotypic variation with the lead CNV genotype. The numbers in the violin plot show the number of accessions for each copy. The significance difference was calculated with two-sided Wilcoxon rank-sum test (**P < 0.01, *P < 0.05)

Domestication-related traits arise from selected genetic variation in wild species, affecting seed size, flowering time, yield, quality, and crop adaptation [35–37]. To identify potential selection signals during cotton domestication, we scanned genetic variations with allele frequency differentiation in nucleotide diversity by comparing each cultivated group with its corresponding wild group. We identified 76 domestication sweep regions (DSRs) using π_Landrace_/π_Improved_ (ratio ≥ 15) and a likelihood method (XP-CLR, Top 5%) (Additional file 2: Figure S6a), occupying 66.8 Mb in the A subgenome and 51.4 Mb in the D subgenome associated with 837 and 1272 genes, including 274 homologous gene pairs (Fig. 2a). Compared with previous studies with small numbers of accessions [3–5], this domestication selection analysis identified 31 novel DSRs occupying 43.6 Mb (Additional file 1: Table S9). Some fiber-related and known domesticated genes were differentially expressed between wild/landraces and improved cultivars (Additional file 2: Figure S6b, c). The domestication selected genes were involved in stress response, cell wall regulation, jasmonic acid, ethylene, and circadian rhythm process (Additional file 2: Figure S7). Further manipulation of these genes in plant hormone pathway and stress response pathway may help illustrate their putative regulatory role in fiber quality improvement and environmental adaptation during cotton domestication [3, 38, 39]. We also identified 120 Mb (π_GhImpUSO_/π_GhImpCHN_ ≥ 2) with improvement signals, including 1006 selected genes in the A subgenome and 2369 in the D subgenome with 353 homologous gene pairs (Fig. 2a; Additional file 2: Figure S6d), and 79.5% (95.4 Mb) of the improvement selection regions were not identified previously [5] (Additional file 1: Table S10). Of note is the observation that 19 Mb of sequence was screened with both domestication and improvement selection signals, in which the D subgenome (441 genes) has more genes than the A subgenome (50 genes) (Additional file 1: Table S11). These data suggest that D subgenome has stronger SNP-based selection signals in both domestication and improvement processes.

Domestication is a driver for CNV allele frequency difference between wild/landrace and domesticated groups [37]. In total, 286 non-redundant CNV-based regions were identified with selection signals during cotton domestication, comprising 297 Mb in the A subgenome (Fig. 2b) and 105 Mb in the D subgenome (Fig. 2f). About 55% (65 Mb of 118 Mb) of SNP-based domestication signals overlapped CNV-based domestication sweeps (Additional file 1: Table S12). In total, 217 CNV regions were identified with improvement selection signals, comprising 156 Mb in the A subgenome and 133 Mb in the D subgenome. About 44% (52 Mb of 120 Mb) of SNP-based improvement signals overlapped the CNV-based improvement signals (Additional file 1: Table S13). In total, we identified 329 Mb (covering 6339 genes) of sequences in the A subgenome and 127 Mb (4955 genes) in the D subgenome with both SNP- and CNV-based domestication signals. A total of 173 Mb (5526 genes) and 184 Mb (8405 genes) of sequences have improvement signals in the A and D subgenomes. The identification of selection signals during domestication and improvement can facilitate to further identify genetic loci of important agronomic traits.

To identify QTLs for selection signals associated with agronomic traits, we conducted a genome-wide association study (GWAS) meta-analysis of 890 *G. hirsutum* accessions from three independent experimental cases with multiple environments (Additional file 3) [3, 5, 6]. Using the genotypic data of 2,291,437 high-quality SNPs with MAF ≥ 0.05 in 890 accessions, we identified 2952 significant SNPs (0.05/2,291,437; *P* < 2.18 × 10^− 8^) associated with fiber quality-related traits. After strict filtering, 91 major fiber-related QTLs were located, including 11 for fiber length (FL), 17 for fiber elongation (FE), 15 for fiber strength (FS), 19 for fiber length uniformity (FU), 10 for fiber micronaire (FM), 7 for fiber maturity (MAT), and 12 for spinning consistency index (SCI) (Additional file 1: Table S14 and Additional file 2: Figure S8). We also identified 31 yield-related and 3 flowering date (FD)-related QTLs. In total, 125 major QTLs with 4751 candidate genes for 15 agronomic traits were identified, in which 78 were consistent with previous studies [3, 5, 6, 15, 40, 41] and the other 47 were newly detected in meta-analysis (Additional file 1: Table S14). In the 125 QTLs, 14 have selection signals during domestication and improvement (Additional file 1: Table S15). In addition, twenty-one QTL loci showed pleiotropic effects on fiber quality, yield, and flowering date (Fig. 2a; Additional file 1: Table S16). For example, lint percentage (LP), fiber weight per boll (FWPB), and lint index (LI) are components of yield trait, with major QTLs co-localized on chromosome D02 (Additional file 2: Figure S9a). The LP, FD, and whole growth period (WGP) for flowering time traits have co-located QTLs on chromosome D03 (Additional file 2: Figure S9b).

We focused on novel QTLs related to fiber elongation that were identified in meta-GWAS. A novel QTL (mqFE253) was located on the D05 chromosome (at 11.3–12.5 Mb of genomic region). The 64 candidate genes were predicted by integrating haplotype analysis, gene expression, and functional annotation (Additional file 2: Figure S10). One candidate gene (*Ghir_D05G013680*, *GhIDD7*), encoding an indeterminate-domain 7 transcription factor, was differentially expressed in four fiber developmental stages (Additional file 2: Figure S10f). Accessions representing two main haplotypes of the 5′ UTR region showed a significant difference in fiber elongation and fiber length (Additional file 2: Figure S11a-b). After knock-out of *GhIDD7*, the mature fiber was significantly shorter than that in wild type plants (25.8 ± 0.3 vs. 27.1 ± 0.1) (Additional file 2: Figure S11c, d, e). These results indicated that *GhIDD7* was a previously uncharacterized gene contributing to fiber quality-related trait.

GWAS analysis of 26,831 high-confidence CNVs (MAF ≥ 0.05) in 419 *G. hirsutum* accessions revealed 370 significant CNVs for 50 QTLs (cnvQTLs) (Additional file 1: Table S17), of which 5 showed pleiotropic effects on both fiber quality and lint yield (Fig. 2a). Thirteen cnvQTLs overlapped with SNP-based QTLs (snpQTLs), and the other 37 cnvQTLs are only identified by CNVs. Of these cnvQTLs, 15 overlapped with domestication sweeps and 10 overlapped with improvement selection signals (Additional file 1: Table S18). The phenotypic data exhibit a significant difference in cotton accessions with different copy numbers of lead CNV (Fig. 2c–e, g–i; Additional file 2: Figure S12). For example, a seed index (SI) association with domestication signal was identified on the A06 chromosome (Fig. 2c). A fiber length (FL) association with domestication signal was located on the A10 chromosome, and FL with 2 duplication copies was significantly longer than that with 0 copy (reference) allele (*P* < 0.01) (Fig. 2d). The lead CNV-involved LD region has 78 candidate coding genes, in which some are involved in cotton fiber development, such as UDP-glucose pyrophosphorylase 3 (*Ghir_A10G024310*, *UGP3*) and AP2/B3-like transcriptional factor (*Ghir_A10G023950*). Another example shows a fiber maturity (MAT) association with improvement selection signal was located on the A12 chromosome (Additional file 2: Figure S13a, b, c). This association has one candidate gene encoding xyloglucan endotransglucosylase/hydrolase 5 (*Ghir_A12G008500*, *XTH5*). In the D subgenome, three cnvQTLs with strong selection signals were found to be associated with FD, FWPB, and FS on the D03, D06, and D07 chromosomes (Additional file 2: Figure S13d, e, f, g). These results provide a number of cnvQTL candidates that may be applied to cultivate desirable traits in future breeding.

### Pan-genomes of *G. hirsutum* and *G. barbadense* species

We used a reference-guided assembly approach [21] to construct pan-genomes of *G. hirsutum* and *G. barbadense*. The sequencing data of 1581 *G. hirsutum* (251 landraces, 424 GhImpUSO and 906 GhImpCHN) and 226 *G. barbadense* improved accessions were aligned to the “TM-1” and “3–79” reference genomes, respectively [12]. About 5800 million unmapped reads from *G. hirsutum* and 1127 million unmapped reads from *G. barbadense* were subject to de novo assembly (Additional file 2: Figure S14, S15), producing 5,047,083,790 bp and 1,517,253,311 bp of contig sequence respectively, with a minimum length of 500 bp (Additional file 1: Table S19). After removing redundancies, 3704 Mb and 1422 Mb non-reference sequences with a contig N50 of 1530 bp (*G. hirsutum*) and 1108 bp (*G. barbadense*) passed all filtering steps for the final non-reference genomes (Additional file 1: Table S20). The final 1041 Mb and 309 Mb non-reference sequences in *G. hirsutum* and *G. barbadense* with a contig length of more than 1000 bp were used for predicting protein-coding genes (Additional file 2: Figure S16). We obtained 32,569 *G. hirsutum* genes (65,679 transcripts) and 8851 *G. barbadense* genes (12,076 transcripts) (Additional file 1: Tables S21-S22). The final *G. hirsutum* pan-genome (Ghpan-genome) is 3388 Mb with 102,768 genes (2347 Mb with 70,199 genes in the “TM-1” reference genome) and *G. barbadense* (Gbpan-genome) is 2575 Mb with 80,148 genes (2266 Mb with 71,297 genes in the “3–79” reference genome) (Additional file 2: Figure S17).

The coverage of the Ghpan-genome was investigated using PacBio reads of 10 representative accessions, including *G. hirsutum yucatanense*, *G. hirsutum richmondi*, *G. hirsutum morrilli* from the wild/landraces, the Acala, Paymaster 54, Stoneville 2B from the GhImpUSO group, and Simian 3, CRI 7, Xinluzao 42, and Xuzhou 142 from the GhImpCHN group (Additional file 1: S23-S25; Additional file 2: Figure S18). After de novo assembly (Additional file 3), more than 93% of assembled contigs were mapped to the TM-1 reference genome. Approximately 18.9 Mb of unmapped contigs (a total of 641 Mb contigs from 10 accessions that were not mapped on the TM-1 reference genome) were aligned to the non-reference sequences of 1581 *G. hirsutum* accessions (the average non-reference sequence length is ~ 655 kb; 1041 Mb/1581 Mb). The PacBio-based assemblies provide evidence for non-reference genome sequences in *G. hirsutum*, indicating that our pipeline of pan-genome construction can retrieve PAVs in a large germplasm population. Some high-frequency PAVs were also verified by PCR in 23 representative accessions (Additional file 2: Figure S19).

**Fig. 3:**
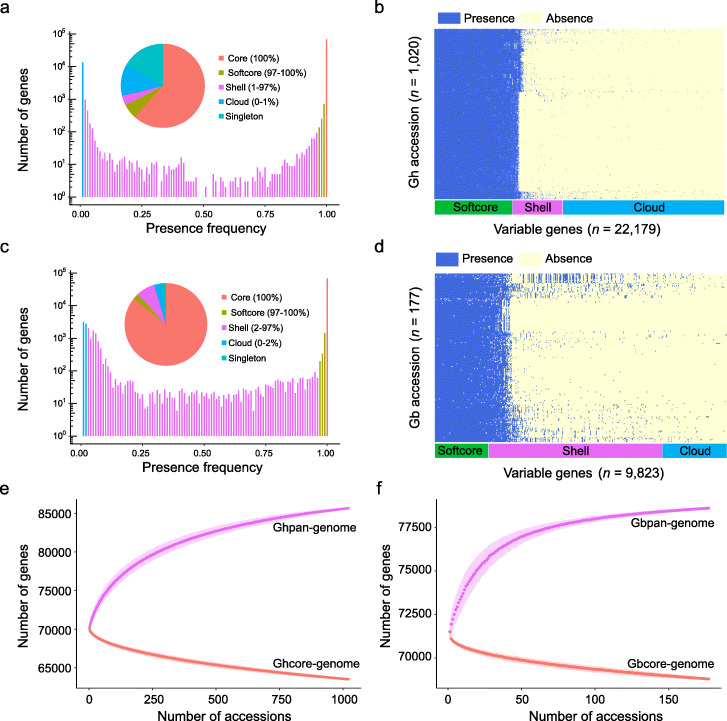
Pan-genomes of *G. hirsutum* and *G. barbadense* species. **a** Gene number and presence frequency in *G. hirsutum* pan genes. The pie chart corresponds to the core (present in all accessions), softcore, shell, and cloud genes. The singleton genes in low-depth (< 5) accessions were excluded for further PAV analysis. The variable genes are divided into reference and non-reference genes in Additional file 2: Figure S17. **b** 1020 *G. hirsutum* accessions heatmap showed presence and absence of variable PAVs. **c** Gene number and presence frequency in *G. barbadense* pan genes. **d** 177 *G. barbadense* accessions heatmap showed presence and absence of variable PAVs. **e, f** Saturation curve modeling the increase of pan-genome size and decrease of core-genome size in 1020 *G. hirsutum* (**e**) and 177 *G. barbadense* (**f**). The error bar was calculated based on 1000 random combinations with five replicates of cotton genomes. The top and bottom edges in purple and red represent the maximum and minimum gene number. The solid lines represent the number of pan genes and core genes

For the *G. hirsutum* population, we mapped re-sequencing reads against 102,768 pan genes, which resulted in 17,100 genes (16.64%, singleton) in 561 accessions (sequencing depth < 5) and 85,667 genes in 1020 accessions (depth > 5). The 1020 *G. hirsutum* accessions include 63,489 core genes shared by all *G. hirsutum* accessions, 5941 (5.78%) softcore genes in 990–1019 accessions (97–100%), 3803 (3.7%) shell genes in 11–989 accessions (1–97%), and 12,434 (12.1%) clouds in less than 10 accessions (0–1%) (Fig. 3a, b). For the *G. barbadense* pan-genome, the 1536 singleton genes only occurred in 49 low-depth accessions. We used 78,612 pan genes that occurred in 177 accessions for further PAV analysis. The 177 *G. barbadense* accessions include 68,789 (85.8%) core genes, 1796 (2.24%) softcore genes in 172–176 accessions (97–100%), 5867 (7.32%) shell genes in 4–171 accessions (2–97%), and 2160 (2.75%) clouds in less than 3 accessions (0–2%) (Fig. 3c, d). Modeling of pan-genome size with iteratively random sampling suggests that the Ghpan-genome has an average of 81,688 pan genes and an average of 65,595 core genes in 398 accessions (Fig. 3e). The Gbpan-genome has an average of 78,607 pan genes and 69,563 core genes in 59 accessions for modeling saturation (Fig. 3f). Therefore, the size of core-genome decreased and pan-genome increased with the increase of population size. GO analysis showed that core genes were involved in cellular metabolic process and development, whereas the variable genes were involved in “defense response,” “response to stress,” and “signaling transduction in environment fitness” (Additional file 2: Figure S20).

**Fig. 4:**
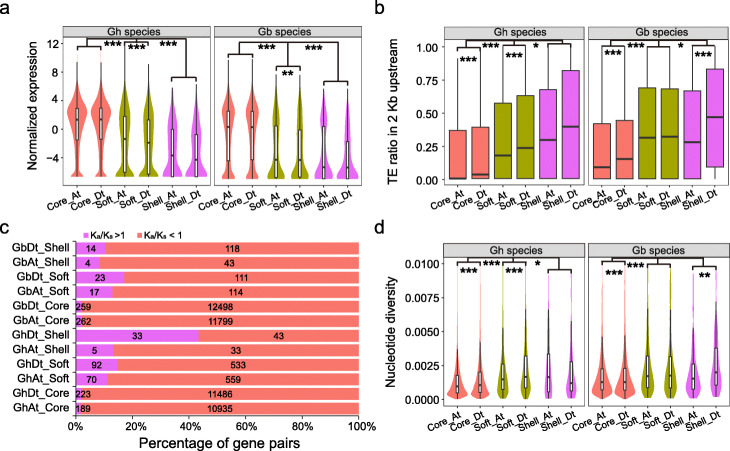
Comparison of core and variable genes in A and D subgenomes. **a** Expression levels of core and variable genes in *G. hirsutum* and *G. barbadense*. The softcore genes are represented by “Soft.” **b** Ratio of transposable element (TE) insertion frequency in upstream 2 kb of core and variable genes in the A and D subgenomes. **c** Ratio of nonsynonymous/synonymous (*K*_*a*_/*K*_*s*_) mutations of core and variable genes. **d** SNP diversity of core and variable genes. The comparison of gene expression, TE, and SNP diversity between core and variable genes were carried out using a two-sided Kolmogorov-Smirnov test (*P < 0.05, **P < 0.01, ***P < 0.001)

We next investigated the genomic characteristics of core and variable genes between A and D subgenome. Core genes have higher expression levels than variable genes in both *G. hirsutum* and *G. barbadense* (Additional file 2: Figure S21). Interestingly, A subgenomic variable genes have higher expression levels than D subgenomic genes (Fig. 4a). Variable genes have a higher adjacent (2 kb) TE insertion probability than core genes, especially for the *Gypsy* class (Additional file 2: Figure S22). The variable genes in the D subgenome have a higher ratio than those in the A subgenome (Fig. 4b). Evolutionary selection analysis showed that more variable genes have undergone positive selection than core genes in both *G. hirsutum* and *G. barbadense*, especially in the D subgenome (Fig. 4c). Furthermore, variable genes have a larger nucleotide diversity than core genes, and more variable genes in the D subgenome have a higher diversity (*P* < 0.001) (Fig. 4d; Additional file 2: Figure S23). These data indicated that D subgenomic variable genes had a faster evolutionary rate than A subgenomic genes.

### PAV selection during domestication and improvement

**Fig. 5:**
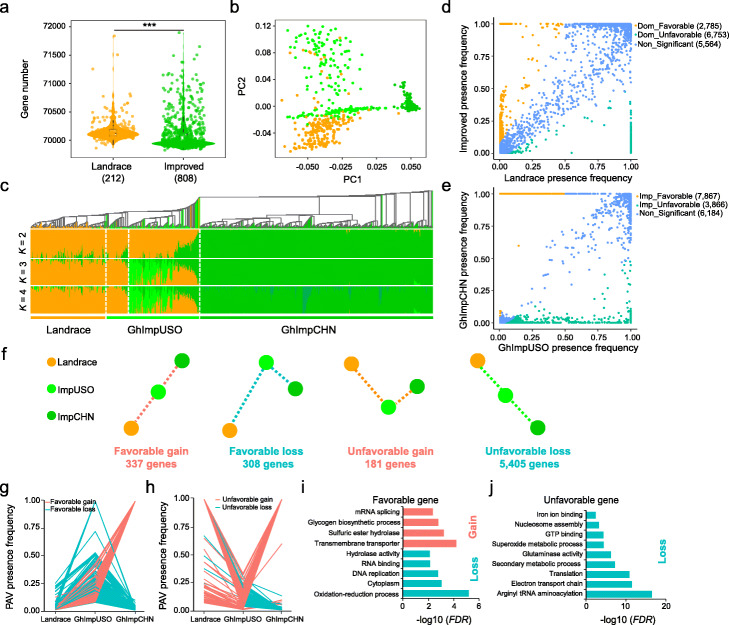
PAV selection signals during cotton domestication and improvement. **a** Gene number among the *G. hirsutum* landrace and improved accessions. The Wilcoxon rank-sum test (*P* < 0.001) was used for the significant statistics. **b** PCA analysis of 1020 accessions based on shell PAVs. **c** Maximum-likelihood phylogenetic tree and population structure with different number of clusters (*K* = 2, 3, and 4) in 1020 *G. hirsutum* accessions using 3803 shell PAVs. The population structure is sorted according to the phylogenetic tree. **d, e** Comparison of significant gene presence frequency between the landrace versus GhImpUSO group (domestication) and GhImpUSO versus GhImpCHN group (improvement) (*FDR* < 0.001, two-sided Fisher’s exact test). **f** Numbers of favorable and unfavorable genes during domestication and improvement. **g, h** PAV presence frequency of favorable and unfavorable genes during domestication and improvement. **i, j** GO enrichment analysis of favorable gene (**i**) and unfavorable gene (**j**) gain and loss during domestication and improvement

To establish landscape of selective PAVs between landrace and improved cotton, we compared PAV frequency between the landrace, GhImpUSO, and GhImpCHN groups. The landrace group has more variable genes than improved cultivars, suggesting a general trend of gene loss during cotton domestication (Fig. 5a). PCA and phylogenetic analysis of PAVs suggest that the landrace group was separated from the improved cultivar group (Fig. 5b, c). The landraces originating from native America had a population mixture with American cultivated cotton in genetic composition, consistent with the clustering analysis of high-confidence SNPs (Additional file 2: Figure S24). To control the false-positive rate, eight landraces and thirty-four GhImpUSO accessions in a mixed population structure with uncertain origin were excluded from further analysis.

To identify PAV-related genes with selection signals during domestication and improvement, we performed two comparisons between 182 landraces and 206 GhImpUSO accessions using the presence frequency of variable genes, for “domestication” (Fig. 5d; Additional file 2: Figure S25), and between 206 GhImpUSO and 592 GhImpCHN accessions for “improvement” (Fig. 5e). The genes with a significant change of presence frequency (*FDR* < 0.001 and frequency fold change > 2 for “unfavorable gene” or < 0.5 for “favorable gene”) were regarded as selected genes. Genes with higher presence frequency in landrace than in GhImpUSO, and higher presence frequency in GhImpUSO than in GhImpCHN were potentially “unfavorable gene,” while genes with reverse patterns of presence frequency were “favorable gene.” We identified 2785 and 7867 favorable genes with allele gain, and 6753 and 3866 unfavorable genes with allele loss during domestication and improvement, respectively (Additional file 1: Tables S26, S27). GO enrichment analysis showed that favorable genes were enriched in oxidation-reduction-related process, whereas unfavorable genes were enriched in fatty acid biosynthesis and gene regulation. The favorable and unfavorable genes were divided into four comparisons according to the presence frequency in three groups during domestication and improvement (Fig. 5f). The continuous selection of 337 favorable genes with both domestication and improvement signals may be elite candidates for breeding, whereas 308 unfavorable genes exhibiting lower presence frequencies in the GhImpCHN group represent loss alleles (Fig. 5g; Additional file 1: Table S28). More unfavorable genes than favorable were eliminated during cotton breeding (Fig. 5h). Favorable gain genes participated in transmembrane transport and oxidation-reduction process, whereas favorable loss genes involved in electron transport chain and secondary metabolic process (Fig. 5i, j). Unfavorable gain genes had no significantly enriched process during improvement (Fig. 5j). These analyses showed that many unfavorable gene were lost during domestication and considerable favorable genes were retained during improvement process.

### Genes for related traits using pan-genome dataset

**Fig. 6:**
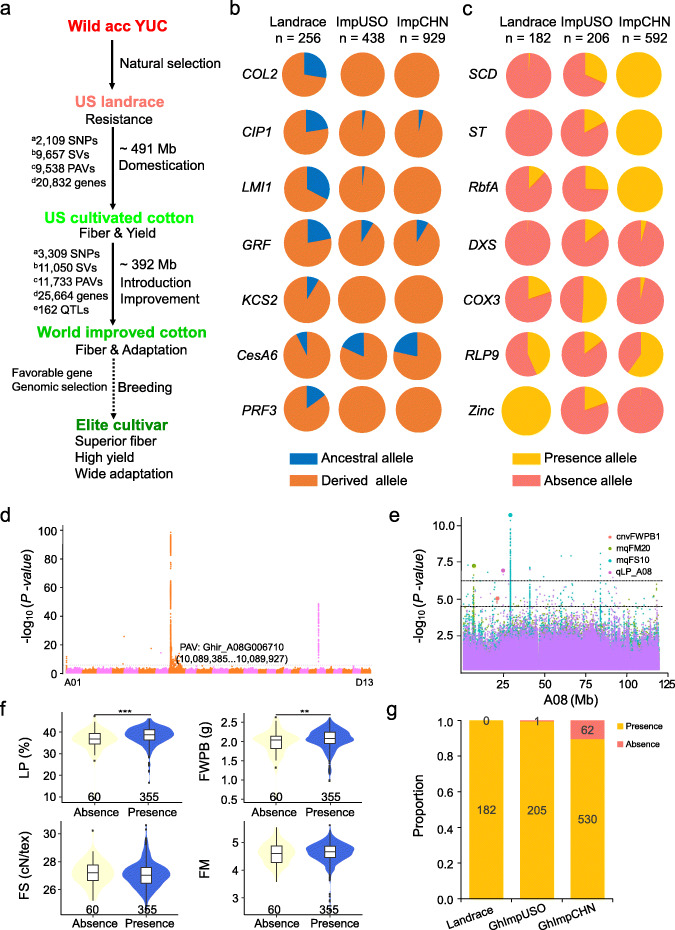
An available pan-genome dataset for cotton breeding. **a** A four-step model of variation during cotton domestication and breeding. **b** The spectrum of gene allele frequencies at the causal SNP polymorphisms of *COL2*, *CIP1, PRF3*, *LMI1*, *GRF*, *KCS2*, and *CesA6* in landrace and two geographic groups. **c** The spectrum of domesticated PAV allele frequ encies of seven genes in landrace and two geographic groups. **d** An example of functional PAV located on the A08 chromosome. The dashed line in Manhattan plot indicates the threshold for GWAS signals (*P* < 2.62 × 10^− 8^; −log *P* > 7.6). This locus includes four QTLs (lint percentage (LP), fiber weight per boll (FWPB), fiber micronaire (FM), fiber strength (FS)). **e** Four QTLs were displayed in a panel of multiple accessions. The two dashed lines represent GWAS thresholds for CNV (−log *P* > 6.45) and SNP (−log *P* > 4.42), respectively. **f** the phenotypic difference between presence and absence groups. The numbers below the violin plots show the accession numbers. The significance difference was calculated with a two-sided Wilcoxon rank-sum test (***P < 0.001, **P < 0.01). **g** Presence frequencies of *Ghir_A08G006710* in 182 landrace, 206 GhImpUSO, and 592 GhImpCHN accessions

Based on the above data, we propose a summary chart for cotton natural selection, domestication, and improvement (Fig. 6a). We identified nearly 456 Mb (19.4% of the assembled reference genome) and 357 Mb (15.2%) of sequences with domestication and improvement signals, through the integrated SNP, CNV, and PAV maps (Additional file 1: Table S29). There are 21,169 genes located in domestication regions, some of which have been demonstrated to be involved in the regulation of flowering date, morphology, and fiber development. For the flowering date, a significant GWAS peak on chromosome D03 has two candidate genes encoding a *COP1*-interactive protein [6] (*CIPI*, *Ghir_D03G008950*) and a CONSTANS-like protein [42] (*COL2*, *Ghir_D03G011010*), which are required for adaptation change in landrace cotton to cultivated varieties in different geographical areas with different photoperiods. Further investigation of causal SNP alleles shows that the ancestral alleles are mainly distributed in landraces, with lower allele frequencies in improved cultivars (Fig. 6b). Similarly, we found that landrace and improved groups exhibited allele differentiation in *LATE MERISTEM IDENTITY1* [43] (*LMI1*, *Ghir_D01G021810*) that regulates leaf shapes, and in the basic helix-loop-helix protein gene *GRF* (*Ghir_A12G025340*) that is a candidate gene for cotton glandular QTL [44] (Fig. 6b). Some genes responsible for fiber development that experienced domestication and improvement selection were also detected by the geographical differentiation analysis. *KCS2* (*Ghir_D10G015750*) and *CesA6* (*Ghir_D03G004880*), responsible for fiber elongation [45–48], were subject to domestication and improvement selection (Fig. 6b). The domestication gene *PRF3* (*Ghir_D13G021640*) has a strongly mutated allele in improved cultivars [49].

Pan-genome analysis uncovered favorable and unfavorable gene alleles during domestication and improvement, providing novel candidate genes for functional investigation (Fig. 5). For genes favorable to cotton improvement selection, *SCD* (short chain dehydrogenase, *GhirPan.00056999*), *ST* (sugar transporter, *GhirPan.00054328*), and *RbfA* (ribosome-binding factor A, *GhirPan.00033905*) have the lowest frequency in wild population and highest in domesticated cultivars (Fig. 6c; Additional file 2: Figure S26). Some favorable genes exhibiting a decrease of frequency in the improvement process could be eliminated (308 genes), having almost the same allele frequency between wild and cultivated accessions, such as *DXS* (deoxyxylulose-5-phosphate synthase, *Ghir_Scaffold1882G000030*) and *COX3* (cytochrome oxidase subunit 3, *Ghir_Scaffold1273G00008*). Genes unfavorable during domestication showed increased (182 genes) or decreased (5405 genes) frequency in the GhImpCHN group, such as *RLP9* (receptor like protein 9, *Ghir_D13G022380*) and *ZBD* (Zinc-binding dehydrogenase, *GhirPan.00044196*) (Fig. 6c).

To determine the contribution of PAV to agronomic traits, we identified PAV-associated SNPs for 1196 PAVs (MAF ≥ 0.02) in 415 accessions (4 accessions were discarded from 419) using 1,904,926 SNPs and obtained 56,486 significant SNPs (*P* < 2.62 × 10^− 8^) associated with 864 (72.2%) PAVs. Of these PAVs, 124 were overlapped with 89 trait-QTLs (Additional file 1: Table S30; Additional file 2: Figure S27). One representative PAV (*Ghir_A08G006710*, 543 bp, an uncharacterized gene in *G. hirsutum*) is located on chromosome A08 (Fig. 6d, Additional file 2: Figure S28). This hotspot region contained two yield-related (LP, FWPB) QTLs and two fiber quality-related (FM, FS) QTLs (Fig. 6e). These accessions with the presence haplotype of this gene showed significantly increased appearance of LP and FWPB traits than those with the absence haplotype, but no difference for FS and FM traits (Fig. 6f). Further presence frequency analysis showed that *Ghir_A08G006710* was present in nearly all landrace and GhImpUSO accessions, but was absent in only a few GhImpCHN accessions (Fig. 6g). Interestingly, in the population RNA-Seq data of 15 DPA fiber [15], absence of this gene in 18 accessions was accompanied by significant low expression of an adjacent gene *Ghir_A08G006730* (locating at upstream ~ 61 kb, encoding an AUX/IAA transcriptional regulator family protein) compared with that representing presence of this gene in 233 accessions, supported by the change of IAA content in fibers of representative accessions (Additional file 2: Figure S29, S30). These results implied that this gene represented a recent loss event with a potential regulatory role in other gene expression during cotton improvement. These PAV localization and QTL analyses may improve the efficiency of identifying favorable genes associated with desirable agronomic traits.

## Discussion

Crop domestication and improvement can alter the extent of genomic variation associated with agronomic traits [35–37]. Previous GWAS analyses identified a number of SNP-based genetic loci (snpQTLs) associated with fiber quality, fiber yield, and flowering date in cotton [3, 5, 6, 14, 15]. Following recently published reference-grade genomes for *G. hirsutum* with “TM-1” and *G. barbadense* with “3–79” [12], in this study, we constructed an integrated genomic strategy to construct variome. Using the variome data, we identified 47 novel SNP-based QTLs and 37 CNV-based QTLs, suggesting the power of QTL identification using a larger collection of genomic data. We found that ~ 19.4% (456 Mb) and ~ 15.2% (357 Mb) of reference genomic regions in *G. hirsutum* have selection signals during domestication and improvement, providing a genetic resource for exploring variations controlling the change of agronomic traits. Using a limited collection of wild and landrace accessions, previous studies have not been able to identify as many selection signals as possible, especially from landrace to the American cotton domestication process [3–6]. This comprehensive variome map provides a new perspective on variation landscape, QTL locations, domestication map, and potential molecular targets for cotton improvement.

Given the notion that variation identification based on mapping data against a single genome cannot fully resolve the entire variation repertoire of germplasm resources, pan-genome analysis provides an ideal alternative for allele mining associated with desirable phenotypes during crop improvement. In this study, we made an attempt of pan-genome assembly using the accessible genomic data. We constructed pan-genomes for *G. hirsutum* and *G. barbadense* species using a conservative reference-guide strategy [21], which include 1041 Mb and 309 Mb extra sequences not captured by the reference genomes. *G. hirsutum* species has a higher percentage of variable genes (38% of 39,278 genes) than *G. barbadense* (14% of 11,359 genes). For Gbpan-genome, the small number of variable genes is due to the fewer number of sequenced accessions. A deep re-sequencing of a larger set of *G. barbadense* germplasm resources is needed to improve the resolution of pan-genome analysis. *G. hirsutum* has a considerable proportion (38%) of variable genes as comparable to other plants, such as wild soybean [18] (51%), *Brassica oleracea* [21] (19%), bread wheat [22] (64%), *Brachypodium distachyon* [23] (45%), rice [24] (52%), tomato [26] (26%), sunflower [27] (25%), sesame [28] (42%), and *Brassica napus* [29] (38%). This proportion of variable genes may be increased with deep re-sequencing data and long-read-based pan-genome assembly. Pan-genome analysis provides an opportunity for understanding of genetic diversity using gene pools to discover gene loss during domestication and improvement, and beneficial alleles and genes in wild counterparts could be used for crop improvement. In this study, PAV presence frequency analyses reveal the loss of 6753 and 3866 genes during cotton domestication and improvement, consistent with the trend found in tomato domestication [26]. We analyzed some PAVs associated with cotton agronomic traits, which allows the identification of potential causal genes (Fig. 6d). Inevitably speaking, the current pan-genome assembly based on short reads leads to many iteratively assembled fragments, so more accessions need deep sequencing to allow de novo genome assembly. The annotation of non-reference genes may be combined with population transcriptome data, which can contribute to more precise annotation of coding genes and non-coding RNAs. In the future, long-read sequencing technologies will be required to integrate new genomics approaches to accurately identify structural variations and construct graphical pan-genomes [50–52].

## Conclusions

In summary, our variome analyses reveal genomic landscape diversity and domestication process in allotetraploid cotton and identify some novel QTLs that may contribute to phenotypic diversity. Pan-genome analyses discover genes that have been lost during domestication and explore the possible impact of some PAVs on fiber traits. Further genetic manipulation of these QTLs and genes will advance precision breeding of this important crop.

## Methods

### Collection of sequencing data of 1961 cotton accessions

Genome re-sequencing data of 1874 cottons were downloaded from National Center for Biotechnology Information (NCBI) database. In this study, we sequenced 87 *G. hirsutum* and *G. barbadense* cultivated accessions (Additional file 1: Table S1). In total, 1961 cotton accessions were obtained, including 1655 *G. hirsutum* (AD)_1_, 270 *G. barbadense* (AD)_2_, 26 wild *Gossypium* species (AD)_3_-(AD)_7_, and 10 diploid (two *Gossypium arboreum* (A_2_), two *Gossypium anomalum* (B_1_), one *Gossypium davidsonii* (D_3_), two *Gossypium gossypioides* (D_6_), three *Gossypium exiguum* (K_1_) accessions) species [3–6, 16, 33, 34] (Additional file 1: Table S1).

### Identification of SNPs and InDels from 1913 accessions

After discarding duplicated accessions, a total of 1913 cottons were used for SNPs/InDels variation calling (Additional file 1: Table S1), including 1623 allotetraploid *G. hirsutum* (AD)_1_, 261 *G. barbadense* (AD)_2_, and 26 wild *Gossypium* species (AD)_3_-(AD)_7_, two *Gossypium arboreum* (A_2_)-genome and one *Gossypium davidsonii* (D_3_) species [3–6, 16, 33, 34]. Two allotetraploid reference genomes (*Gossypium hirsutum* acc. TM-1 and *Gossypium barbadense* acc. 3–79) [12] and their annotations were downloaded from CottonGen https://www.cottongen.org/. Raw pair-end reads were filtered with Trimmomatic (v0.32, MINLEN: 75) [53]. Clean reads were aligned against reference genomes using BWA-MEM (v0.7.10-r789). Duplicated mapping reads were filtered using picard-tools, and uniquely mapped reads were retained for further analysis. The reads around InDels from BWA [54] alignment were realigned by GATK (v4.0.1) [55] with RealignerTargetCreator and InDelRealigner programs with parameter setting -stand_call_conf 30. To obtain high-confidence variants, we retained the shared variants by GATK and SAMtools [56] with sequencing depth of at least 6. The scaffolds were excluded from further analysis. Finally, GVCF files were merged with “CombineGVCFs” and the missing rate was filtered by VCFtools. The missing genotypes were imputed using Beagle (v5.0) with hidden Markov model [57]. A total of 119,678,187 SNPs and 12,354,432 InDels were identified from 1913 cotton accessions. The detailed filtering processes were as follows: (1) high-quality SNPs set with 1% ≤ minor allele frequency (MAF) ≤ 99% and InDels with a maximum length of 20 bp were retained. Missing rates of more than 80% were discarded in specified populations; (2) a filtered set of ~ 25 million SNPs from ~ 63 million bi-allelic SNPs was retained with MAF ≥ 0.01; (3) core SNP set of ~ 19 million was obtained from ~ 25 million SNPs with criteria of at least five accessions having homozygote for each SNP. The core variation set has 19,246,497 SNPs and 4,815,125 InDels which were used for further analysis (Additional file 1: Tables S2 and S3). The different sub-population SNPs were filtered with the same criteria. The core SNPs and InDels were annotated using ANNOVAR program [58].

### Population structure analysis of 1913 accessions

We chose randomly 5 SNP sets (200,000 SNPs for each) from the core SNP set (19,246,497) for population structure analysis. First, STRUCTURE [59] was run on 5 × 200,000 SNPs with *K* ranging from 3 to 15, and *K* = 12 was determined as the optimal value by the Structure Harvest subprogram with the “evanno” method. The Q-matrices were merged using CLUMPP [60] based on the sorting of *K* = 12. One random SNP set was used to construct a neighbor-joining tree with PHYLIP (v3.696) [61] with 1000 bootstrap replicates and was visualized using online tool iTOL (https://itol.embl.de/). Principal component analysis (PCA) was performed using gcta64 [62] program with MAF ≥ 0.05. The weighted Fixation statistics (*F*st) and nucleotide diversity (π) were calculated by VCFtools (v1.1.14) [63] in a 100-kb sliding window with a step size of 20 kb.

### Linkage disequilibrium (LD) analysis

Linkage disequilibrium (LD) was calculated using PLINK (v1.90b6.10) [64] with parameter settings (--ld-window-r^2^ 0 --ld-window 99999 --ld-window-kb 1000). The pairwise *r*^2^ values were calculated by two SNPs across whole genome. The LD decay plot was shown in average 1 kb bins using a Perl script.

### Genome-wide selective sweep analysis

We identified genomic selection and improvement signals using two strategies. For the domestication regions, we combined two major cultivated cotton groups (438 accessions from USO and other geographical regions, 929 accessions from China) into an improved group to exclude genetic drift. In total, 256 landraces and 1364 improved *G. hirsutum* accessions were used for domestication sweeps. Nucleotide diversity (*π*) was calculated from landrace, improved GhImpUSO and GhImpCHN groups. The ratio of nucleotide diversity (*π*_Landrace_/*π*_Improved_) for landrace versus improved cultivars was used to define candidate domestication selection regions. XP-CLR [65] (v1.0, -w1 0.005 200 2000 1 -p0 0.9) method was used to filter candidate domestication regions. To perform XP-CLR analysis, SNPs were assigned at genetic positions according to a released genetic map [12]. The top 5% XP-CLR values were selected. The overlapping regions in *π* ratio and XP-CLR analyses were identified to be high-confidence domestication sweep regions. The adjacent DSR signals were merged. The GhImpUSO/GhImpCHN ratio was used to identify improved regions during breeding. The domesticated homeologous gene pairs were detected by the reciprocal best BLAST hit between At- and Dt-subgenomes. Syntenic blocks were detected using MCScanX [66]. The expression levels of domestication-related genes were calculated between wild/landraces and improved cultivars using data in previous studies [3, 47].

### Identification of structural variations

The DELLY (v0.7.2) [67] program was used to identify structural variation (SV), integrating the strategies of read depth, read pair, and split read for SV identification. DELLY was used to identify deletions (DEL), insertions (INS), duplications (DUP), inversions (INV), and translocations (TRA) for each accession. Breakdancer (v1.3.6) [68] was also used to identify insertions, deletions, inversions, and inter- and intra-chromosomal translocations for each accession according to the mapped pair-end reads with unexpected separation distance or orientation. Breakdancer-max (-q 20 -y 30) was used for SV identification of each accession. The shared breakpoints of SVs were subject to a filtering process with mapping read depth of more than 10×. The SVs in all cotton accessions were merged into a population-scale VCF file using BCFtools. For the analysis of SV genotypes, the high-quality SVs filtered as “LowQual” and “IMPRECISE” were further retained only with split-read (SR) consensus alignment of more than 3 and the length of more than 50 bp and less than 1 Mb. Two adjacent SVs were combined as a single SV if the distance between start coordinate of one SV and end coordinate of the other SV was less than 500 bp, and the overlapping region occupied more than 50% of the total size.

### Identification of CNVs

The copy number variations (CNVs) were detected using CNVcaller [69]. Briefly, the reference genome was split into 800 bp overlapping sliding windows. Second, we generated the reference genome index and processed the BAM file of each accession. The boundaries of CNV regions (CNVR) were detected using normalized mean read depth (RD). The CNV minimum frequency of gain/loss individuals (-f 0.05), homozygotes (-h 3), and RD of adjacent windows are significantly correlated (-r 0.5). At last, the CNV genotype were clustered with the input sample using a Gaussian Mixture Model. The minor allele frequency of 0.01 was used in each specific population.

### Meta-genome-wide association study for fiber and agronomic traits

We performed a genome-wide association study on three independent experiments for fiber length (FL), fiber strength (FS), fiber micronaire (FM), fiber elongation (FE), length uniformity (FU), boll weight (BW), lint percentage (LP), seed index (SI), lint index (LI), fiber weight per boll (FWPB), and flowering date (FD), using re-sequencing data of 267 accessions from Huazhong Agricultural University (HZAU) [3, 14], 263 accessions from Nanjing Agricultural University (NJAU) [5], and 419 accessions from Hebei Agricultural University (HBAU) [6]. After discarding accessions with missing phenotypes, a total of 264, 207, and 419 accessions from the HZAU, NJAU, and HZAU were retained, respectively. We merged best linear unbiased prediction (BLUP) [70] values of 890 non-redundant accessions in three independent experiments to conduct Meta-GWAS. The 2,787,330, 677,013, 2,371,414 and 2,291,437 high-quality SNPs (MAF ≥ 0.05 and homozygote more than five accessions) were used for GWAS analysis in 264,207,419,890 accessions using the TASSEL5.0 [71] with a mixed linear model (P + G + Q + K) and FastLMM [72], respectively. The significant threshold was set as 1/*N* (independent case), and 0.05/*N* (Meta-GWAS) as filtering parameter (“*N*” represents the total number of SNPs). For the CNV-based GWAS, we used 26,831 CNVs identified from 419 *G. hirsutum* accessions released by the HBAU (MAF ≥ 0.05) to identify CNV-based QTLs.

### Pan-genome construction based on short reads

The unaligned reads were extracted using SAMtools with “-b -f 4” and “-f 68 -F 8 and -f 132 -F 8.” We assembled all unmapped paired reads and unpaired single reads for each accession with MaSuRCA (v3.2.1) [73] assembler (cgwErrorRate = 0.15, PE = “PE 300 50,” LIMIT_JUMP_COVERAGE = 300, KMER_COUNT_THRESHOLD = 1). The initial contigs with a length of longer than 500 bp were retained. The long contigs were aligned against reference genome using nucmer (-c 90 -l 40) program in MUMmer (v4.0.0) package [74]. The redundant sequences were filtered using CD-HIT (v4.8.1) [75] with command “-c 0.9 -G 0 -aL 0.90 -AL 500 -aS 0.9 -T 0 -M 1500000.” The contig filtering steps are as follows: (1) remaining sequences from cotton chloroplast genome (GenBank: DQ345959) and mitochondrial genome (GenBank: JX944505.1) were identified using BLASTN and MUMmer package with nucmer “-l 90.” To ensure that contigs of each accession were absent from the reference genome, we aligned these contigs against the reference genome; (2) unaligned contigs from the archaea, bacteria, and viral genomes (Jun 18, 2019) were discarded using the Kraken (v2) [76]; (3) non-redundant contigs were used to search the NCBI nt database (20171030) using BLAST (-e 1e-05; -best_hit_overhang 0.25 -perc_identity 0.8; -max_target_seqs 10) to identify other contaminants; (4) remaining contigs were subject to an all-versus-all alignment with nucmer and BLASTN (-e 1e-05, -b 200 -v 200) to ensure the non-redundancy. Contig sequences with a similarity of 90% and a length of 90% were filtered out among the cultivars. The non-reference sequences that were not aligned to sequences of higher plants in NCBI nt database were considered as contaminant sequences, according to previous rice pan-genome [24]. The Ghpan-genome sequences were generated by combining the 2347 Mb of “TM-1” reference sequences and 1041 Mb of final non-reference sequences. The Gbpan-genome sequences consisted of 2266 Mb of “3–79” reference sequence and 309 Mb of final non-reference sequences.

### PacBio sequencing and de novo assembly of 10 representative cotton accessions

According to phylogenetic tree, three wild/landrace accessions, three GhImpUSO, and four GhImpCHN cultivars of *G. hirsutum* for different sub-population were used for evaluating Ghpan-genome coverage. Genomic DNA was extracted from young leaves using CTAB method. The PacBio library was constructed and sequenced on PacBio Sequel platform. Long reads were assembled using MECAT (ErrorRate = 0.02) assembler [77]. The 70× depth Illumina pair-end reads were used to polish the PacBio assembly using pilon (v1.23) program [78]. After two rounds of polishing, 2,550,224 SNPs, 13,154,090 insertions, and 2,151,774 deletions were corrected on average for each cotton accession. The assemblies of wild accessions, landraces, and modern cultivated cotton were subject to assessment of assembly completeness using BUSCO (v3.1.0) [79] with embryophyta_odb9 database as a reference. This showed that 1369 (95.1%), 1380 (95.8%), and 1374 (95.4%) integrity BUSCO hits were found for landrace, GhImpUSO, and GhImpCHN groups, respectively (Additional file 2: Figure S18b).

### Annotation of pan-genome genes

We used de novo and homology-based prediction of non-reference genes (only contigs with a length of more than 1000 bp were used for gene prediction). First, RepeatModeler (v1.0.11) (http://www.repeatmasker.org/RepeatModeler/) was used for de novo construction of repeat library in the non-reference genome, and the repeat sequences were masked by RepeatMask (v4.0.7) [80]. The protein-coding genes were predicted in non-reference genomes with MAKER2 pipeline [81]. Gene prediction included ab initio prediction and protein homology-based prediction. For ab initio gene prediction, AUGUSTUS (v3.3.1) [82] and SNAP (v2006-07-28) [83] were trained for two rounds by MAKER. Cotton expressed sequence tags (ESTs, MAY 2019) were downloaded from NCBI and aligned against the non-reference sequences using BLASTN. Cotton protein sequences were downloaded from NCBI and UniProtKB databases and were aligned against the non-reference sequences with BLASTX. We excluded non-reference genes with less than 500 bp on both sides of contigs. These transcripts were aligned to reference transcripts to remove potential redundant transcripts. These non-reference transcripts were also subjected to all-by-all alignment. The final protein sequences translated from transcripts were aligned using InterProScan (v5) [84]. Transcripts with at least one evidence (Interpro, Pfam, GO, KEGG) supporting annotation were retained.

For the functional annotation of non-reference genes, protein sequences were aligned against the NCBI non-redundant (nr) and InterProScan (v5). GO enrichment analysis of core and variable genes were performed for Ghpan-genome and Gbpan-genome using Fisher’s exact test method.

### Gene presence/absence variation (PAV) analysis

First, the raw reads from each accession were aligned to the pan-genome sequences using BWA-MEM with default parameters. The PAVs were detected by SGSGeneLoss [85] (v1.0) with at least two covered reads (minCov = 2, lostCutoff = 0.2). If more than 80% of exon regions were covered, this gene was called present with the “1/1” genotype. We defined variable genes and divided them into three categories: softcore, shell, and cloud genes. For the *G. hirsutum* pan-genome, the softcore, shell, and cloud genes were present in 97–100%, 1–97%, and less than 1% of the accessions in specified population, respectively. For *G. barbadense* pan-genome, the softcore, shell, and cloud genes were present in 98–100%, 3–97%, and less than 2% of the accessions in specified population, respectively. The *K*_*a*_ and *K*_*s*_ values were calculated to estimate evolutionary rate by KaKs_Calculator (v2.0) [86] with multiple alignments of core, softcore, shell, and cloud genes using MAFFT (v7.453) [87]. The shell and cloud genes were combined as flexible genes. For the PAV population analysis, we selected PAV genes present in specified population and discarded un-mapping non-reference genes. The phylogenetic tree was constructed using IQ-TREE [88] program based on the binary flexible PAV genes with 1000 bootstraps according to a maximum-likelihood method. The pan-genome saturation curve analysis was repeated for 1000 random combinations with five replicates of cotton genome orders starting with two and ending with 1020 of *G. hirsutum* accessions, and 177 of *G. barbadense* accessions.

### Selection of PAVs during cotton domestication and improvement

To identify PAVs undergoing selection during cotton domestication and improvement, the PAV presence frequencies were calculated in two groups (landrace versus Improved USO and Improved CHN for domestication; GhImpUSO versus GhImpCHN for improvement). The significantly different PAV frequency for each gene between domestication and improvement groups was calculated using Fisher’s exact test. The *P* value was determined in all PAVs and was then corrected via false discovery rate (*FDR*). PAVs with significantly different frequencies (*FDR* < 0.001 and Ghlandrace/GhImproved fold change > 2 defined as “unfavorable” or fold change < 0.5 “favorable”) were identified as those with domestication/improvement selection signals. The continuously selected genes in landrace, GhImpUSO, and GhImpCHN sub-population were defined as “favorable gain” (gene presence frequency: GhImpCHN > GhImpUSO > landrace), “favorable loss” (GhImpUSO > landrace and GhImpCHN < GhImpUSO), “unfavorable gain” (landrace > GhImpUSO and GhImpCHN > GhImpUSO), and “unfavorable loss” (GhImpCHN < GhImpUSO < landrace).

### Identification of PAV-associated SNPs

To associate SNPs with PAV genes, we analyzed linkage disequilibrium between PAVs and SNPs using FastLMM [72]. According to the *G. hirsutum* PAV analysis, 1196 PAVs (MAF ≥ 0.02) were used for genome-wide association analysis in 415 accessions (exclude abnormal samples) [6]. The PAV presence and absence served as the “phenotype,” and 1,904,926 SNPs served as “genotype” according to a previous study [27]. To control the false-positive rate of significant hits, we used a threshold of 0.05/*N* to filter association peaks. The significant PAV loci that overlapped with trait-QTLs were considered to be associated with agronomic traits, and the location of QTLs represented the positions of non-reference PAVs. Reference PAV-associated trait-SNPs were selected manually.

### CRISPR/Cas9 mutagenesis experiment

Computational sgRNA design for *Ghir_D05G013680* gene and vector construction were described in a previous study [89]. *Gossypium hirsutum* cultivar accession Jin668 was used for *Agrobacterium*-mediated transformation as described in our previous study [90]. The transgenic cotton plants were confirmed by genotyping polymerase chain reaction (PCR), and then positive individual was used for Hi-TOM target sequencing [91]. The T0 transgenic positive plants were transplanted in the greenhouse, in order to harvest T0 seeds. The T1 generation plants were cultivated in the experimental field of Huazhong Agricultural university. The edited T1 transgenic line was validated by Sanger sequencing. The fiber quality-related traits were measured with 10 cotton bolls from T1 and wild type plants by a High-Volume Instrument (HVI) (HFT9000, Premier, India).

### Phytohormone measurement

About 150-mg fiber samples were extracted twice with cold methanol (80% [v/v]) by sharking overnight at 4 °C with three biological replicates. Indole-3-acetic-2,2-d2 acid (IAA; Sigma-Aldrich), ^2^H_6_-abscisic acid (ABA; Olchemim), and (±) 9,10-dihydro-jasmonic acid (JA; Olchemim) were added to each sample as an internal standard. The quantification of IAA, ABA, JA, and jasmonoyl-isoleucine (JA-Ile) was performed on an ABI 4000 Q-Trap system (Applied Biosystems) according to a method described previously [92].

## Supplementary Information

**Additional file 1: **Table S1. Summary of genomic sequencing data of 1961 cottons in this study. Table S2. Summary of SNP filtering in 1913 *G. hirsutum*, *G. barbadense* and other *Gossypium* species accessions for each chromosome. Table S3. Summary of InDels filtering in 1913 accessions for each chromosome. Table S4. Summary of SNP filtering in 1623 *G. hirsutum* accessions for each chromosome. Table S5. Summary of SNP and InDel filtering in 261 *G. barbadense* accessions for each chromosome. Table S6. The SNP and InDel annotation of subpopulation. Table S7. The number of structural variations filtered by four steps in each subpopulation. Table S8. Summary of copy number variations for each chromosome in different subpopulation. Table S9. The SNP-based domestication sweeps and covering genes. Table S10. The SNP-based improvement signals and covering genes. Table S11. Continuously selected signals and genes during domestication and improvement. Table S12. The CNV-based domestication signals and overlapped SNP-based signals. Table S13. The CNV-based improvement signals and overlapped SNP-based signals. Table S14. Significant SNP-based GWAS signals for 15 agronomic traits. Table S15. Summary of snpQTLs that overlapped with domestication and improvement signals. Table S16. Summary of pleiotropic snpQTLs in multiple panels. Table S17. Summary of pleiotropic cnvQTLs in 419 panel accessions. Table S18. Summary of cnvQTLs that overlap with domestication and improvement signals. Table S19. Summary of assembly non-reference contigs between *G. hirsutum* and *G. barbadense*. Table S20. Filtering non-reference sequences following several filtering steps. Table S21. Protein-coding genes predicted in the *G. hirsutum* non-reference genome. Table S22. Protein-coding genes predicted in the *G. barbadense* non-reference genome. Table S23. Summary of PacBio reads and Illumina reads for 10 representative *G. hirsutum* cotton accessions. Table S24. Summary of polished contigs for 10 representative *G. hirsutum* accessions and draft genomes mapped to the GhPangenome. Table S25. Meta-genome likely assembly for landrace, American and modern cotton variety groups. Table S26. Selected PAVs during cotton domestication. Table S27. Selected PAVs during cotton improvement. Table S28. Selected PAVs during both domestication and improvement. Table S29. Summary of selective regions and covering genes during domestication and improvement. Table S30. Detail information of functional SNP variation in landrace and two geographic groups. Table S31. Summary of PAVs associated with QTLs.

**Additional file 2.** Figures S1-S30.

**Additional file 3.** Supplementary Notes.

**Additional file 4.** Review history.

## Data Availability

The short reads of 87 cotton accessions and PacBio long reads of 10 representative *G. hirsutum* accessions have been submitted to NCBI under the accession number PRJNA576032 (https://www.ncbi.nlm.nih.gov/bioproject/PRJNA576032, [93]. The variome data set (SNPs, InDels, CNVs, PAVs), non-reference sequences, annotated genes of *G. hirsutum* and *G. barbadense* species, and other source data have been deposited in Figshare database (https://figshare.com/s/cb3c104782a1dcd90ab0) [94].
