# Supplementary material for: Cotton pan-genome retrieves the lost sequences and genes during domestication and selection
Source: Genome Biol. 2021 Apr 23;22:119. doi: 10.1186/s13059-021-02351-w (PMC8063427; doi:10.1186/s13059-021-02351-w)
Supplement: Supplementary file 2 — Additional file 2. Figures S1-S30. [file 13059_2021_2351_MOESM2_ESM.pdf]

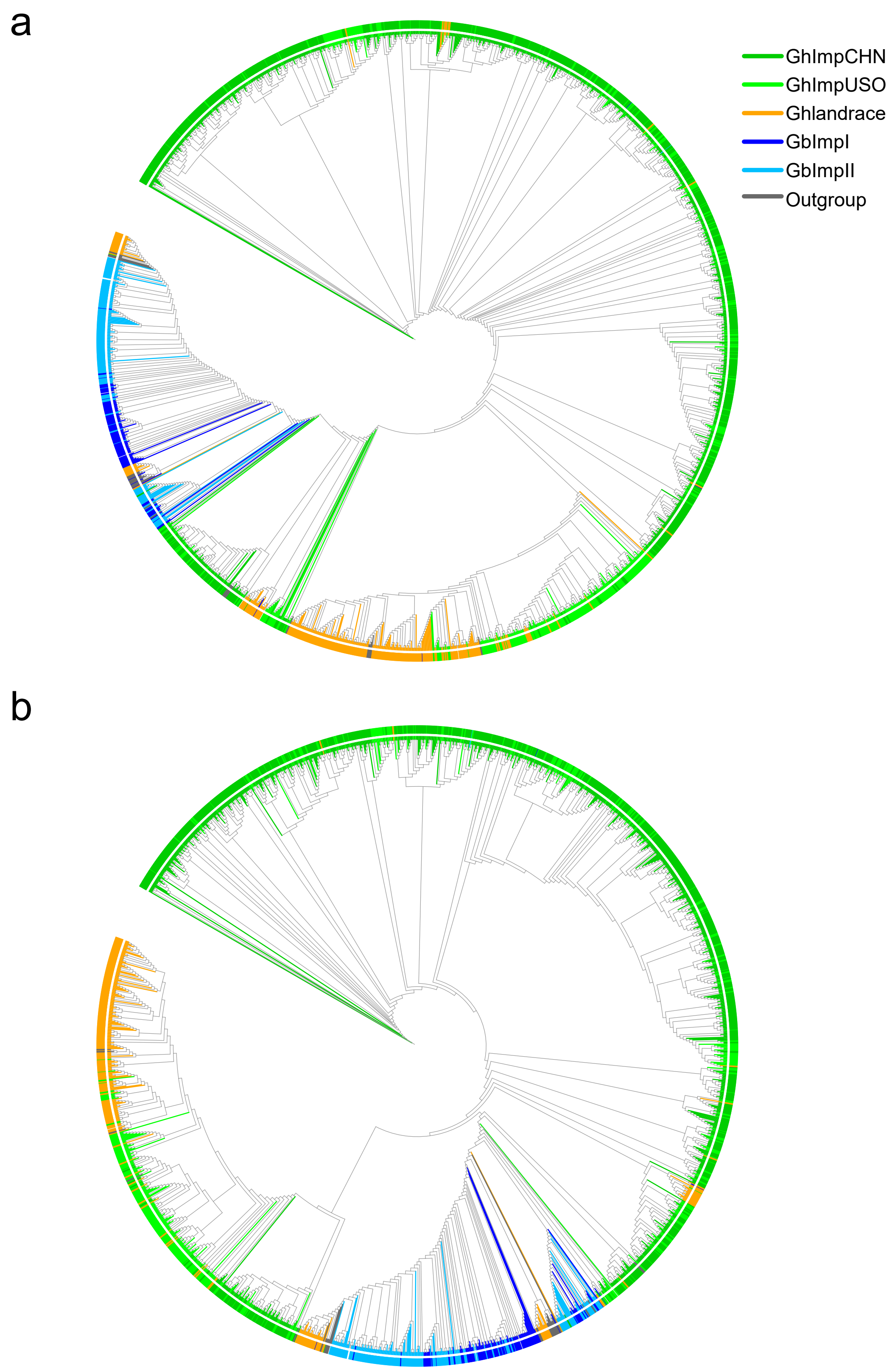

Figure S1

Phylogenetic tree for 1,913 cottons in the A (a) and D (b) subgenomes.

Colors represent different groups or species as indicated in **Fig. 1b**.

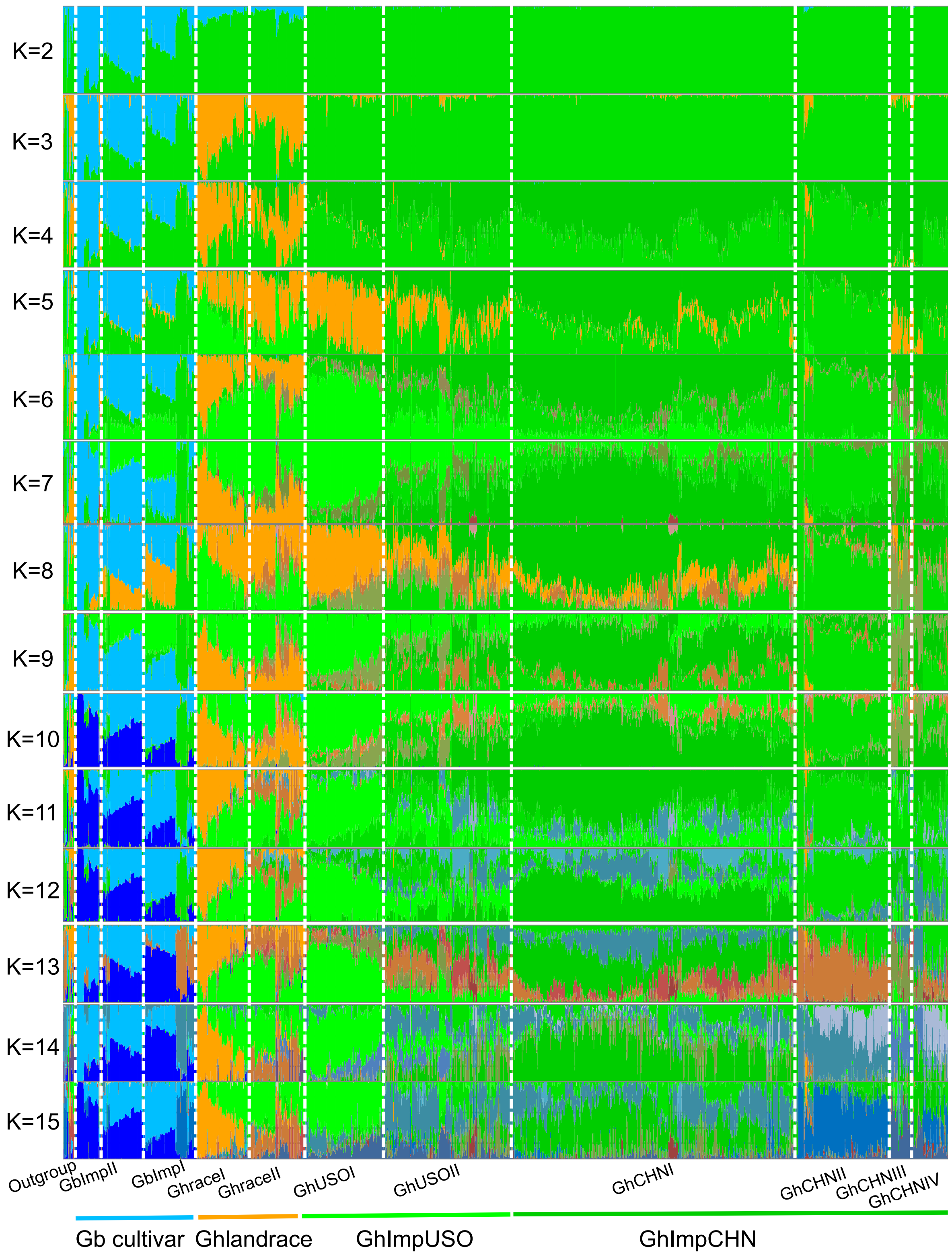

Figure S2

Model-based clustering analysis with different numbers of clusters assuming *K* = 2 to *K* = 15 in 1,913 cottons. The y axis quantifies genetic diversity in each accession, and the x axis lists the different cotton accessions. The orders and positions of these accessions on the x axis are sorted based on K = 12 using the CLUMMP best result. The x axis lists the outgroup species, *G. barbadense* (light blue and dark blue), *G. hirsutum* landraces (orange), *G. hirsutum* improved USO regions (GhImpUSO) (green1 and improved China regions (GhImpCHN) (green3), respectively.

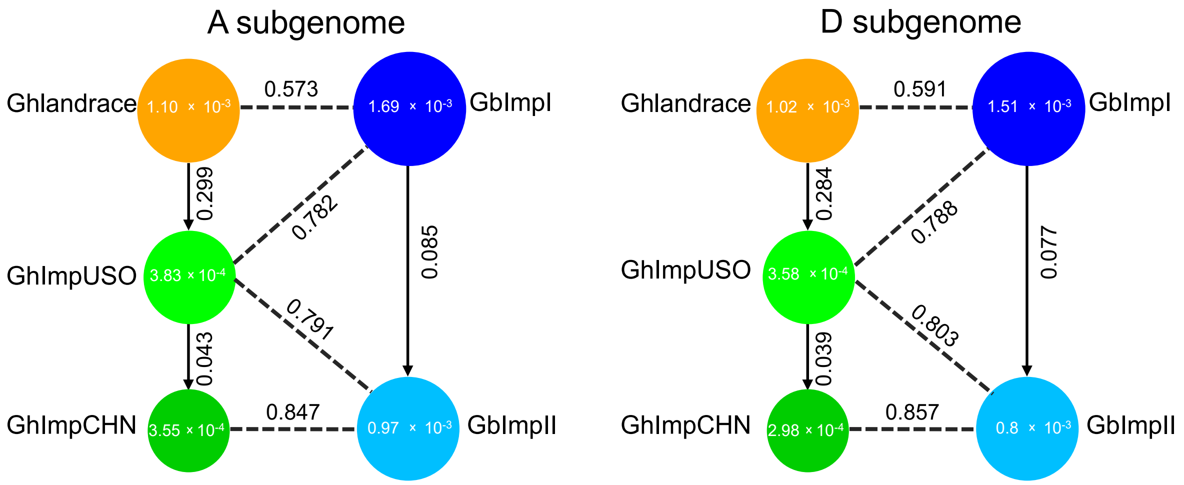

Figure S3

Nucleotide diversity (π) and fixation index divergence (*F*st) in the A and D subgenomes for five groups. The A and D subgenomes are shown in **Fig. 1d.** For each group, the values in the circles represent genetic diversity (π), and the values between the groups indicate population differentiation (*F*st). The solid arrows represent the domestication and improvement process.

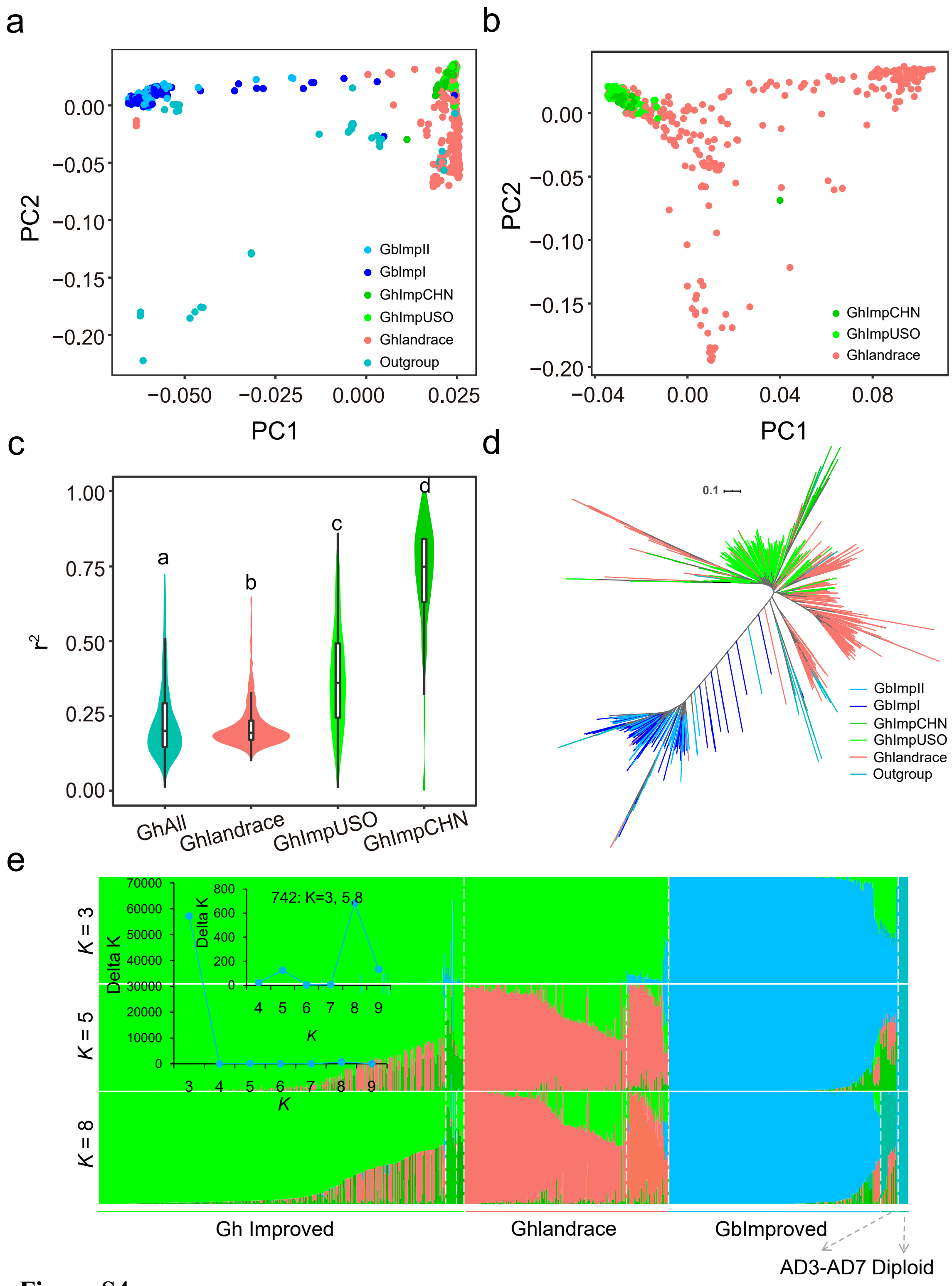

Figure S4

Population structure and genetic diversity in *G. hirsutum* and *G. barbadense* accessions based on CNVs. a PCA of the 742 cottons based on CNVs. This population include 192 Gb accessions, 251 Ghlandrace, 240 GhImpUSO, 30 GhImpCHN accessions, 10 diploid species, 19 AD_3-AD_7 tetraploid cottons. **b** PCA plot of 521 *G. hirsutum* landraces and improved cultivars. **c** LD properties of CNVs (Two-sided Wilcoxon rank-sum test for adjacent groups, $P < 0.001$). **d** Phylogenetic tree of 742 cottons based on 173,166 CNVs. **e** Population structure analysis of 742 cottons based on random 20,000 CNVs according to $K = 8$ sorting for $K = 2$ to $K = 10$.

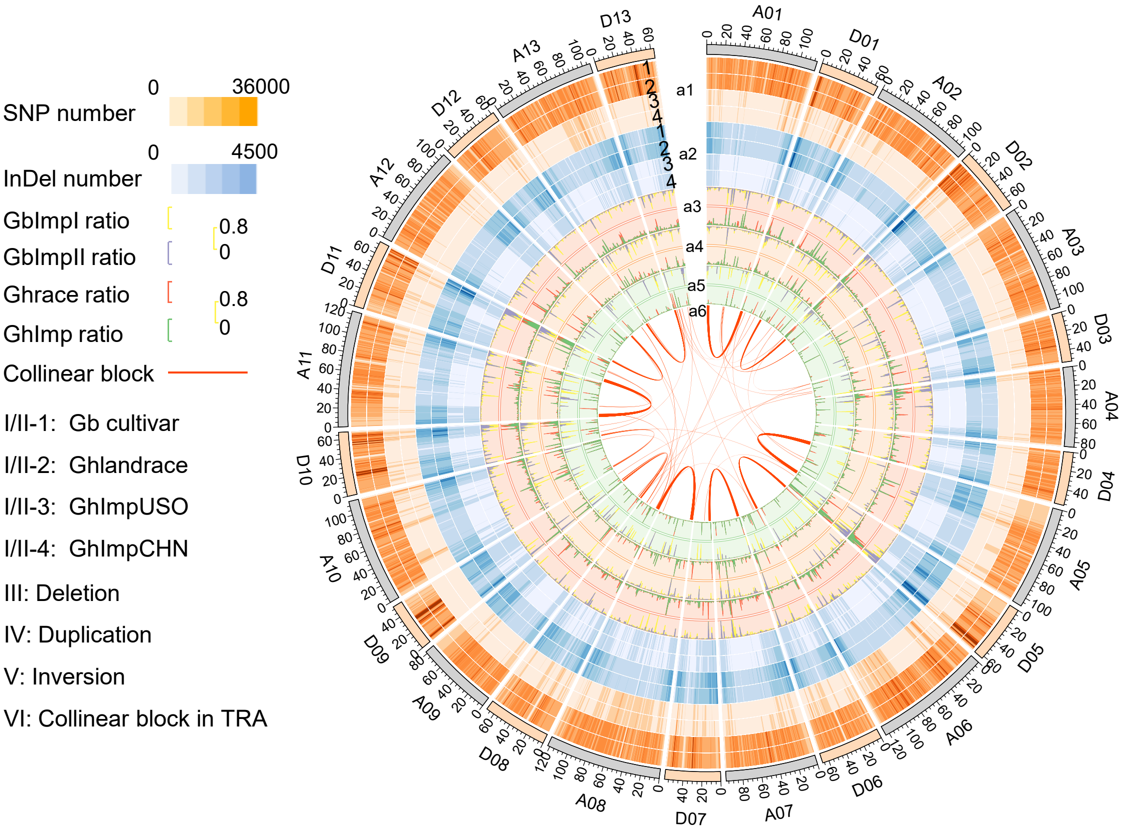

Figure S5

Circos plot of cotton variome. The chromosomes in the A and D subgenomes were divided into 1 Mb windows sliding 200 Kb. I-VI, The SNP and InDel density in each chromosome. The outer to inner tracks represent the SNP density (I) and InDel density (II) for *G. barbadense* cultivars, Ghlandrace, GhImpUSO, GhImpCHN, respectively. III-V, The ratio of deletions (DELs), duplications (DUPs), inversions (INVs) and translocations (TRAs) of SVs in GbImpI, GbImpII, Ghlandrace, Ghimproved population (GhImpUSO and GhImpCHN). VI, The 617 collinear blocks for translocations (TRAs).

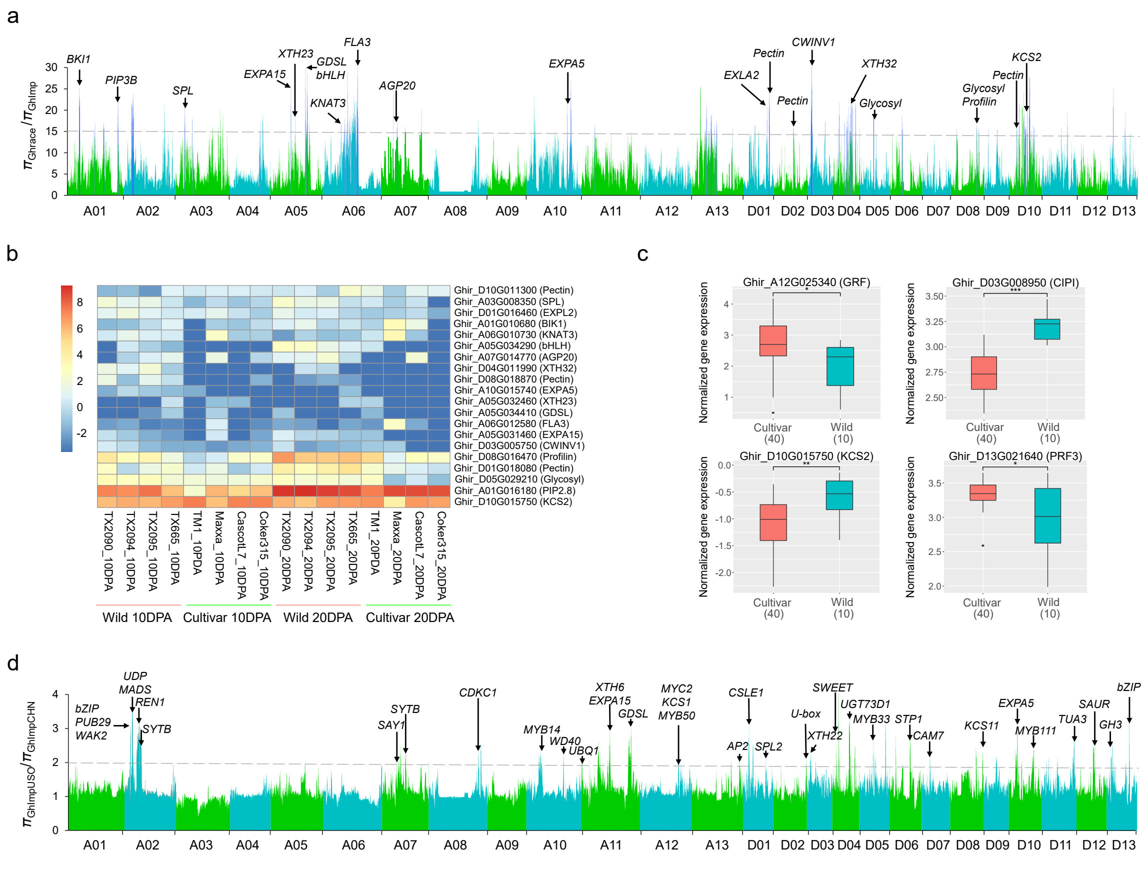

Figure S6

Selection signals and genes during cotton domestication and improvement. a The horizontal grey dashed lines showed the genome-wide threshold for domestication signals identified from the ratio of nucleotide diversity (π) between 256 landraces and 1,364 improved cotton accessions ($\pi_{\text{landrace}}/\pi_{\text{Improved}} \geq 15$). The overlapped high confidence regions from the XP-CLR software results were highlighted by the blue histogram. **b** Expression analysis of 20 (**a**) fiber-related domesticated genes in four wild/landrace and four cultivar accessions at the 10 DPA and 20 DPA fibers. This data was from a previous study (Yoo and Wendel, 2014). Gene expression level was calculated using Fragments Per Kilobase of exon model per Million mapped fragments (FPKM), and further normalized by log2(FPKM + 0.1). **c** Gene expression of domesticated marked gene in young leaves between 10 wild/landrace and 40 improved cultivars. Numbers in parentheses indicated the number of accessions. This data was from our previous study (Wang et al., 2017). **d** Whole-genome analysis of the selective improvement signals through the comparison between GhImpUSO and GhImpCHN accessions ($\pi_{\text{GhImpUSO}}/\pi_{\text{GhImpCHN}} \geq 2$). Some functional genes associated with fiber development have been highlighted above the selection peaks.

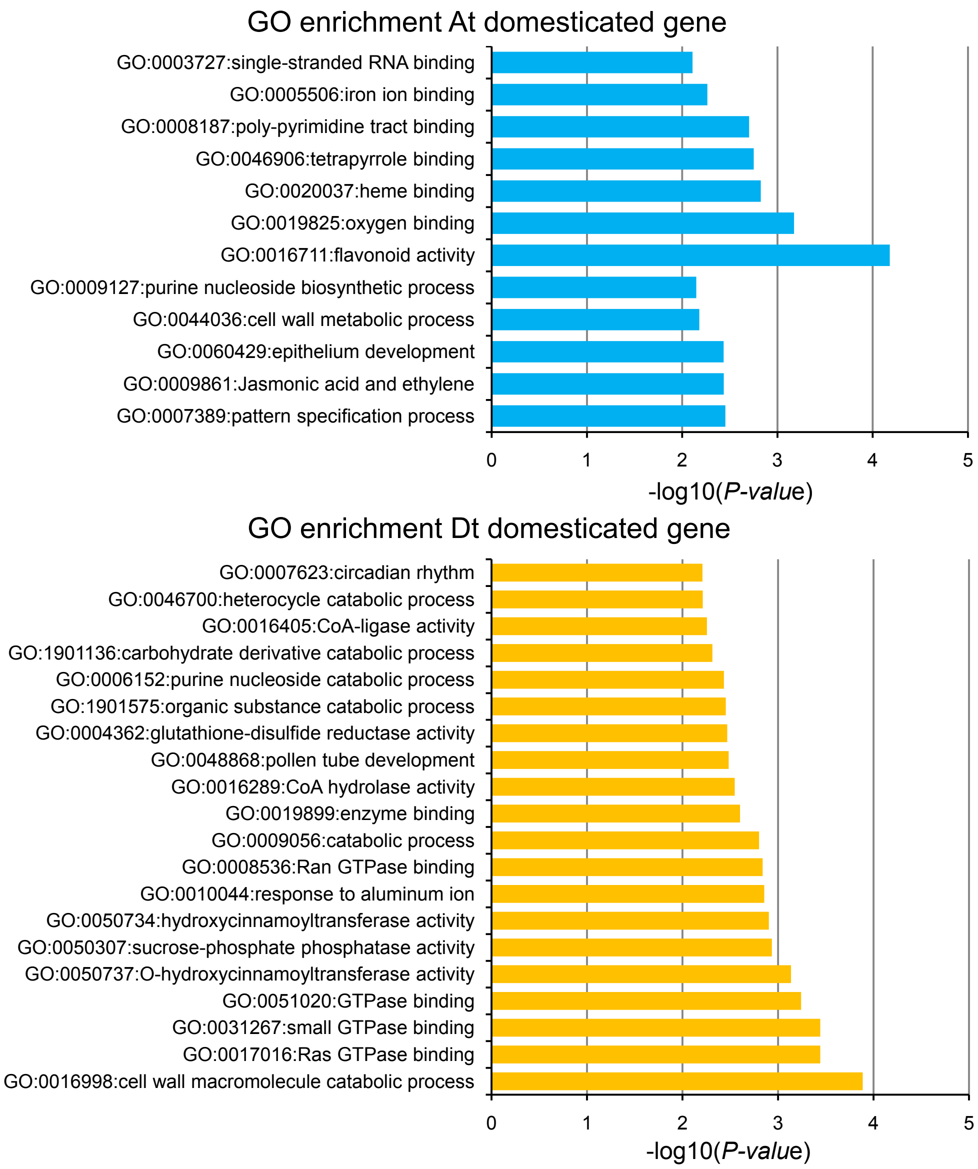

Figure S7

GO enrichment analysis of 837 domestication genes in the A-subgenome and 1,272 domestication genes in the D-subgenome. The *P*-value was calculated by the Fisher's exact test ($P < 0.01$).

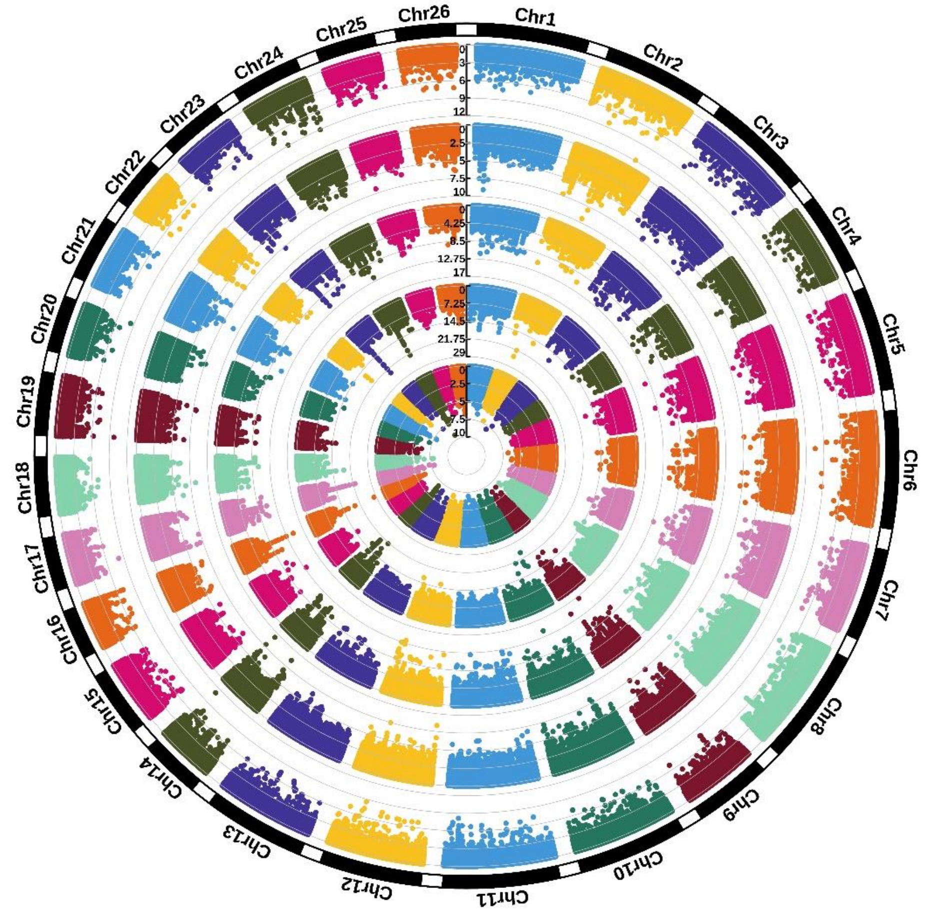

Figure S8

Genome-wide association meta-analysis study of fiber length (FL), fiber elongation (FE), fiber strength (FS), fiber length uniformity (FU), fiber micronaire (FM) in 890 accessions. Breeding values were calculated using the R package lme4 to eliminate the environmental effect from independent case study of 207, 264, 419 accessions, which were combined into 890 non-redundant accessions. BLUP breeding values for 13 agronomic traits were calculated across 12 different environments in 419 accessions (Ma et al., 2018). BLUP breeding values for 15 gronomic traits were calculated across 12 environments in 264 accessions (Huang et al., 2016; Wang et al., 2017). BLUP breeding values for 15 agronomic traits were calculated across 9 environments in 207 accessions (Fang et al., 2017. The final Meta-QTL information was provided in **Table S14**.

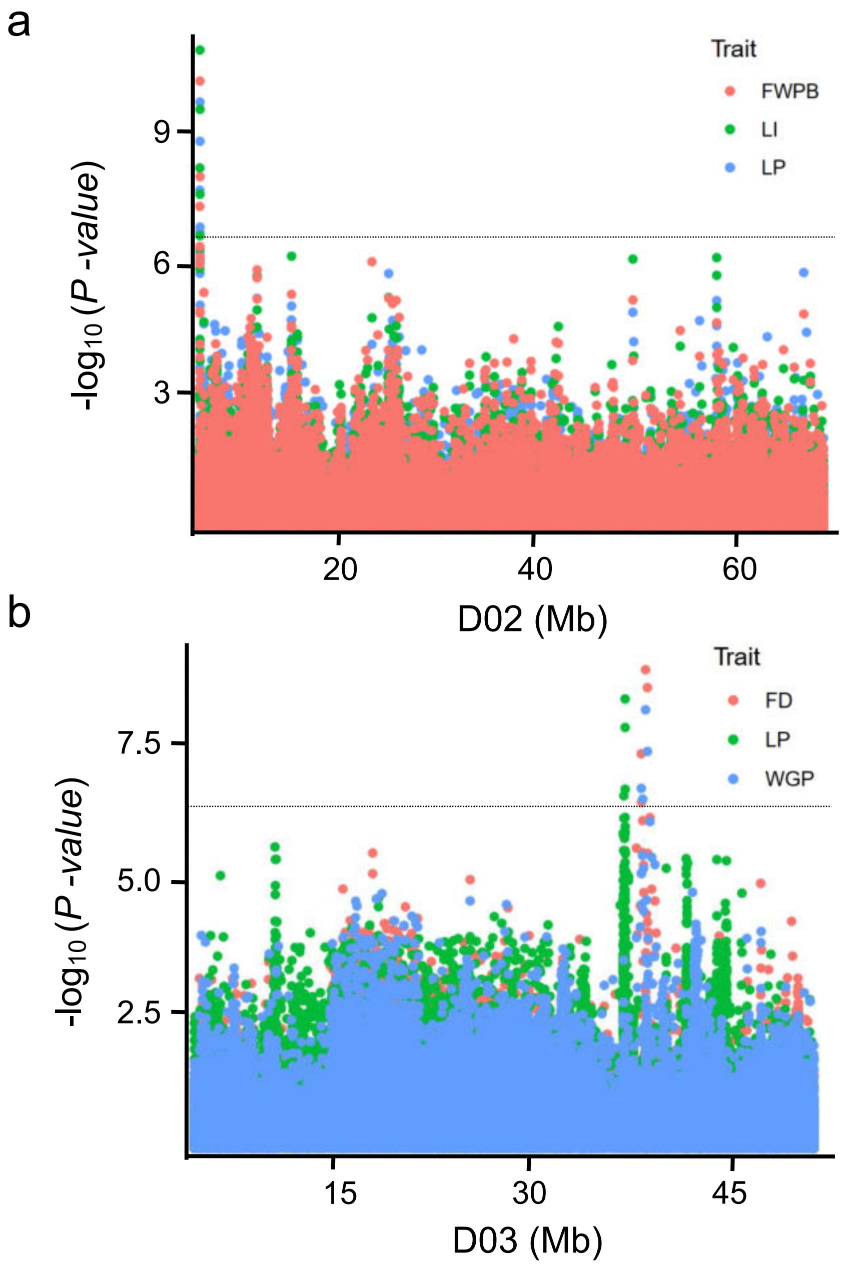

Figure S9

Example of two pleiotropic QTLs.

a Genome-wide association analysis study for a pleiotropic QTL on chromosome D02 using 419 panel accessions. BLUP breeding values were calculated across 12 different environments (Ma et al., 2018). Dashed lines in Manhattan plot indicate the threshold for GWAS signals ($P < 4.22\times10^{-7}$; $-\log P > 6.4$). **b** Genome-wide association analysis study for a pleiotropic QTL (hotspot) on chromosome D03 using 264 panel accessions. BLUP breeding values were calculated in 12 different environments (Huang et al., 2017). Dashed lines in Manhattan plots indicate the threshold for GWAS signals ($P < 3.5\times10^{-7}$; $-\log P > 6.45$).

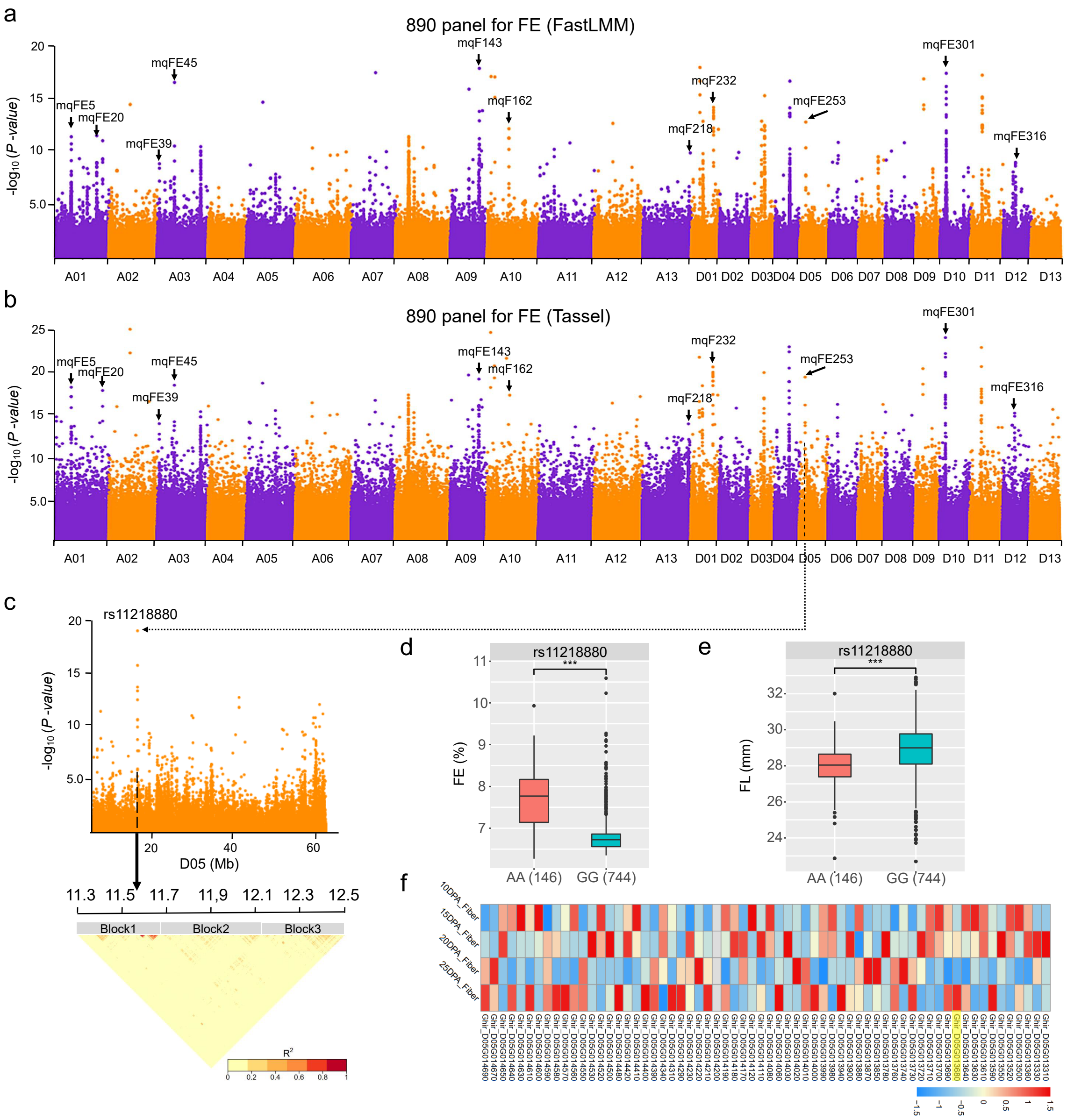

Figure S10

Novel QTL associated with fiber elongation (FE) in 890 panel accessions.

a-b GWAS analysis for fiber elongation in 890 panel accessions using FastLMM (**a**) and TASSEL (**b**). The black arrow represents novel QTL. **c** GWAS identification of FE_D05 novel QTL. Manhattan plot (upper) of FE and LD heatmap (lower) of 1.2 Mb region. The lead SNP significant value is 1.08E-19 ($-\log_{10}P$ = 18.96). Three LD blocks were highlighted by grey boxes. **d-e** The FE and FL variation with lead SNP haplotype **f** Expression of 72 candidate genes during four stage fiber development. Days post-anthesis (DPA).

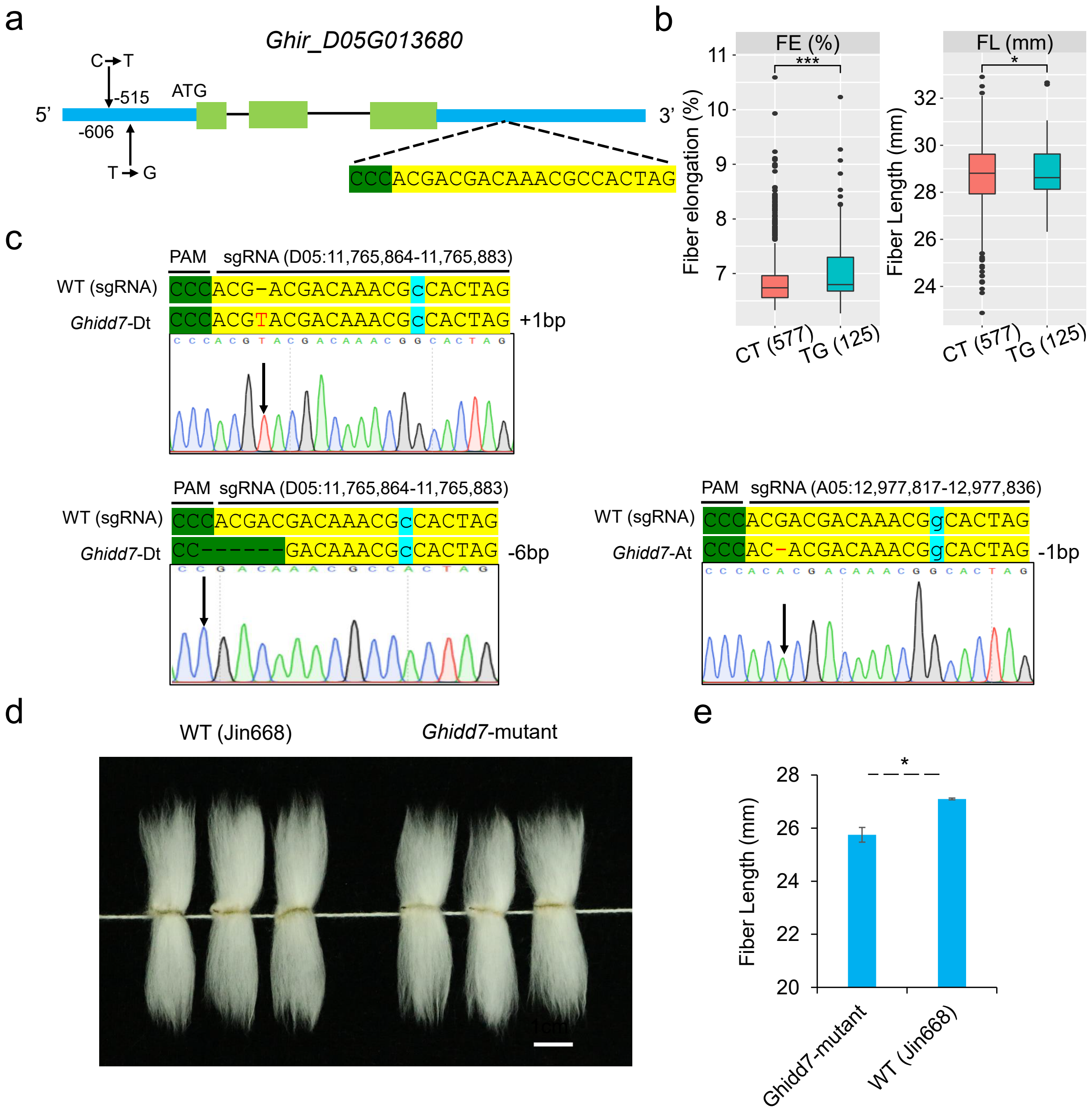

Figure S11

A novel candidate gene (*Ghir_D05G013680*) on chromosome D05 was validated by CRISPR/Cas9 experiment. a Gene model of candidate gene. There are two SNPs in 5' UTR of Ghir_D05G013680. The lower panel shows the sgRNA genome location. The '-515' and '-606' indicate two functional variations of 5' UTR. **b** Box-plot of fiber elongation (FE) and fiber length (FL) phenotype variation in two major haplotypes. These two mutations mainly differentiated into two haplotype combinations: CT containing 577 accessions and TG containing 125 accessions. **c** Ghir_D05G013680 and Ghir_A05G013930 homologous genes were knocked out by CRISPR/Cas9 genome editing. The 1 bp insertion / 6 bp deletion (Dt) and 1 bp deletion (At) were shown in homologous gene. The lower panel illustrated the Sanger sequencing spectrogram. **d** Phenotype of mature fibers of CRISPR/Cas9 mutant at the T1 generation and wild type (WT). Scale = 1 cm. **e** Mature fiber length between CRISPR/Cas9 mutant and wild type (WT) (Two-sided t-test, * *P* = 0.0012).

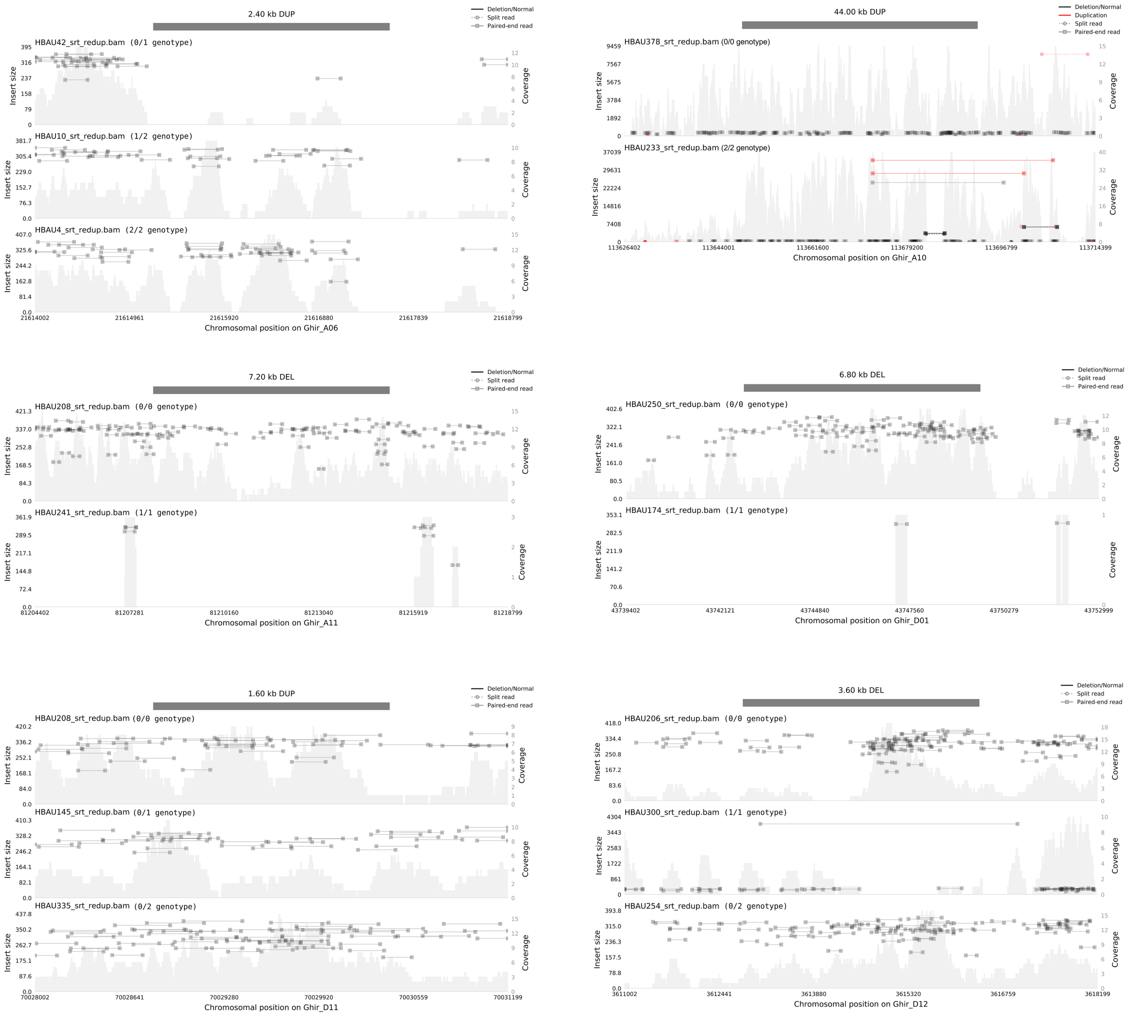

Figure S12

Genome alignment for the lead CNVs in representative accessions. The DUP and DEL represent duplication and deletion variation, respectively. The results of six lead CNV mapping result were related to **Fig.2**.

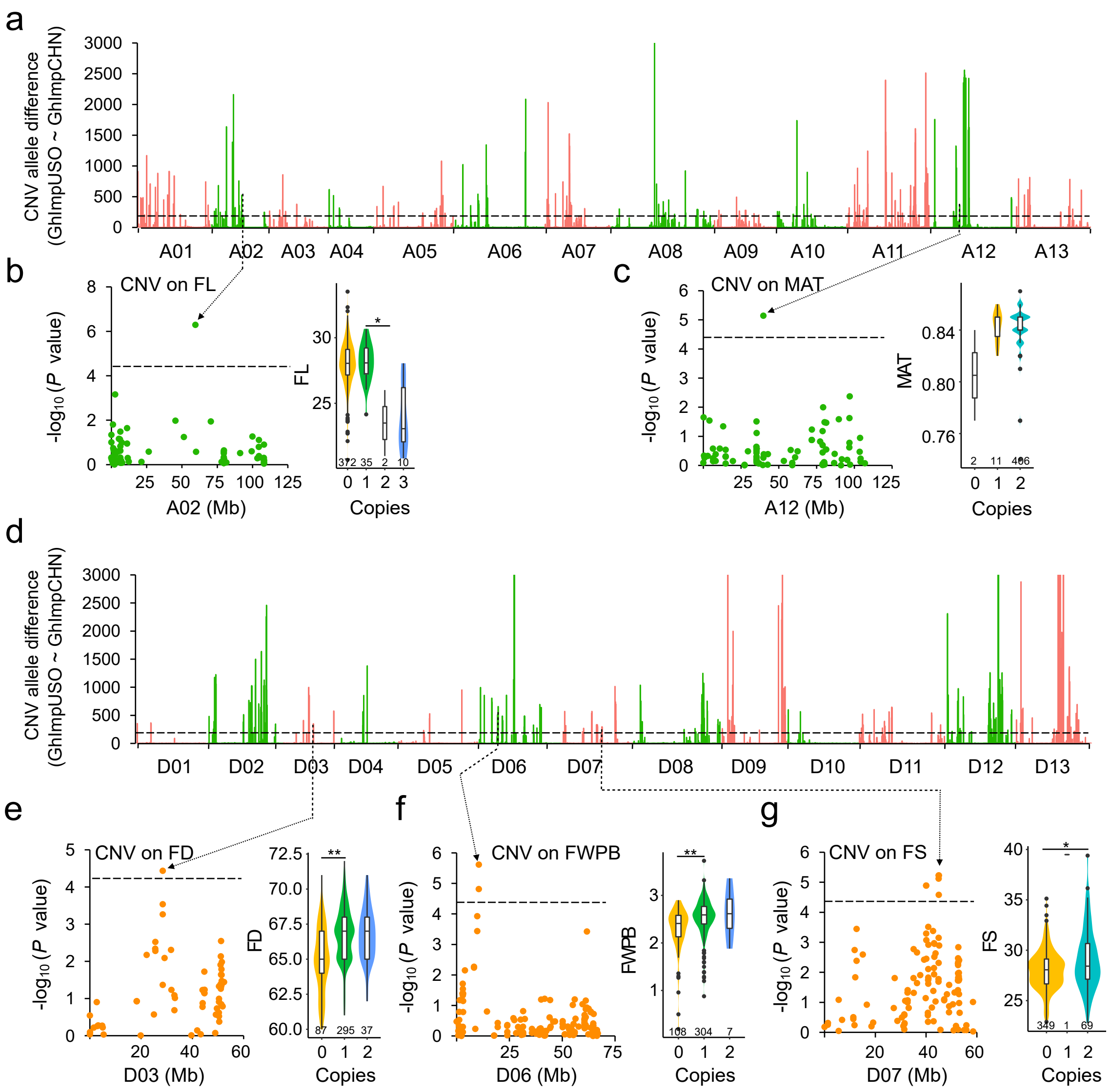

Figure S13

CNV selection signals and GWAS on fiber quality and yield traits during cotton improvement. a and **d** CNV-based improvement selection signals in the A (**a**) and D (**d**) subgenomes. The horizontal dashed lines show the improvement signal threshold with the ratio of nucleotide diversity between GhImpUSO and GhImpCHN cotton accessions ($\pi_{GhImpUSO}/\pi_{GhImpCHN} > 200$). **b** and **c** Two CNV-based GWAS associations overlapped with fiber length (**b**), and maturity (**c**). **e-g** Three GWAS hits strongly overlapped with the improvement selection CNVs for flowering date (**e**), fiber weight per boll (**f**), and fiber strength (**g**) in the D subgenome. The number in the violin plot below is number of accessions for each copy. The significance difference was calculated with two-sided wilcoxon test (** P <0.01; * P <0.05).

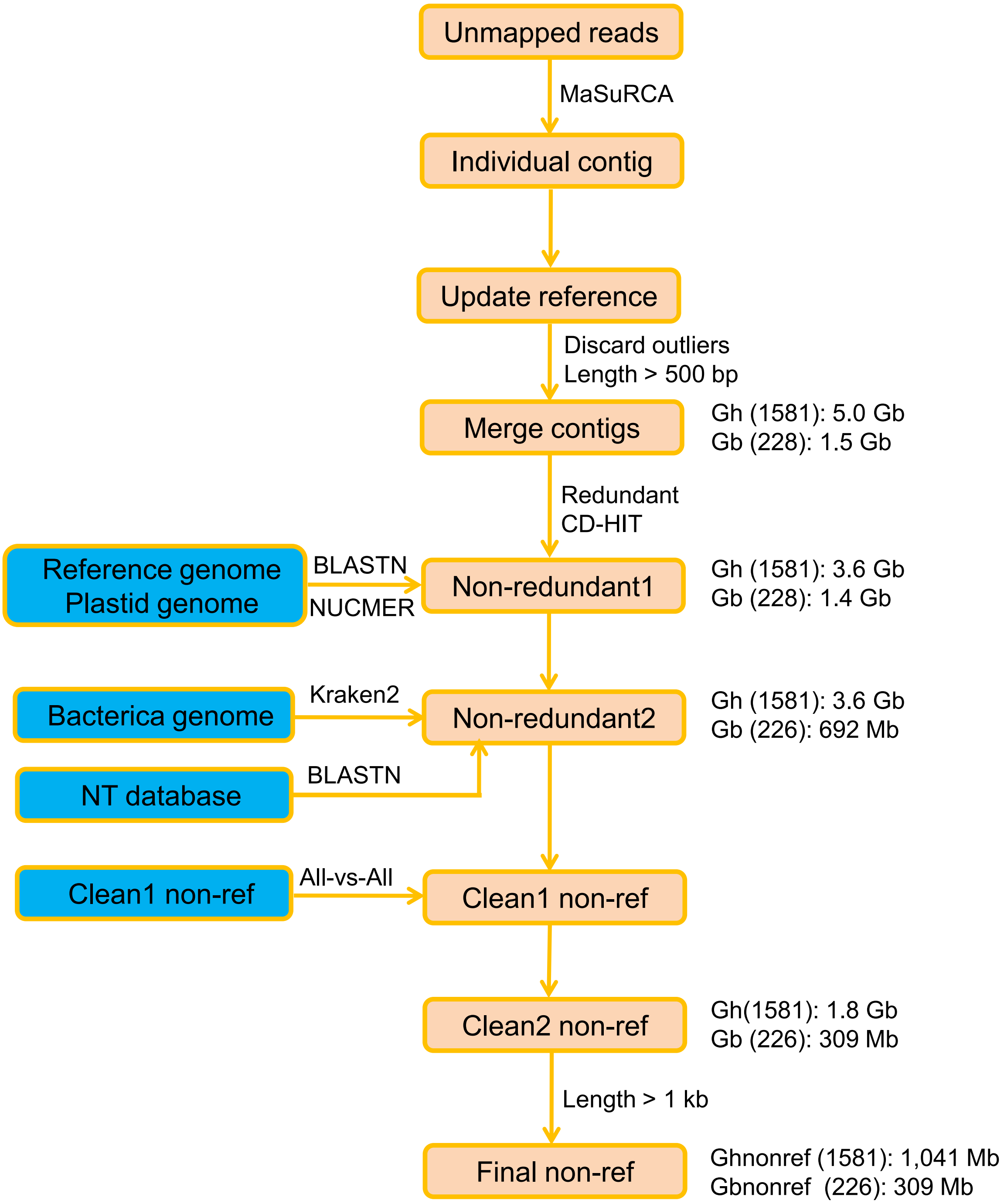

Figure S14

Pipeline for pan-genome construction and filtering steps in *G. hirsutum* and *G. barbadense* based on unmapped reads with MaSuRCA assembly. Each step contains the results of filtering in each subpopulation.

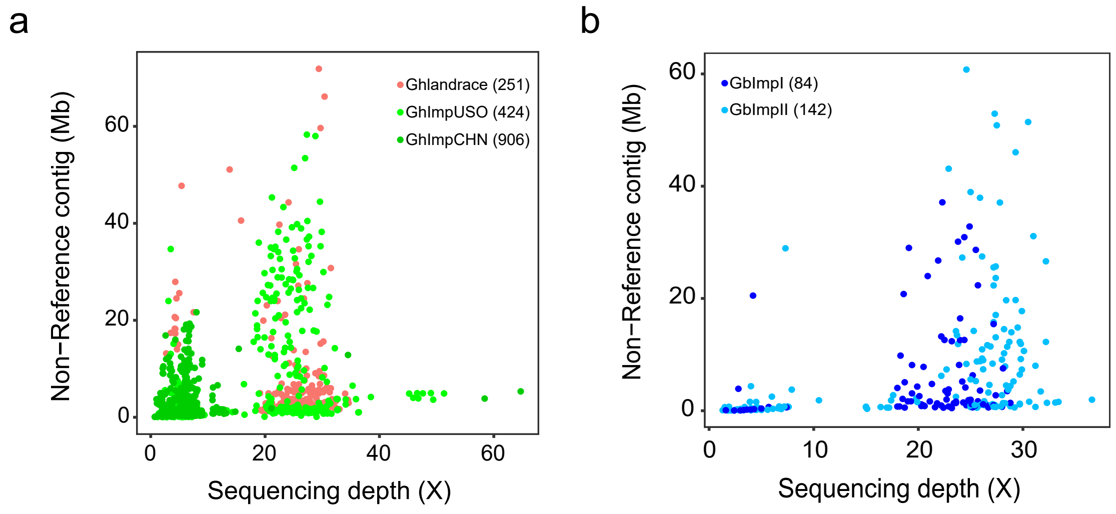

Figure S15

Correlation between different sequencing depth and assembled non-reference contig size (Mb). a Correlation of assembled non-reference contigs and cotton accessions with different sequencing depths of 251 accessions in landrace, 424 accessions in GhImpUSO and 906 accessions in GhImpCHN population ($r^2 = 0.33$, $P < 2.2\times10^{-16}$). **b** Correlation of assembled non-reference contigs and cotton accessions with different sequencing depths of 226 *G. barbadense* accessions (Pearson's correlation coefficient, $r^2 = 0.32$, $P < 9.02\times10^{-7}$).

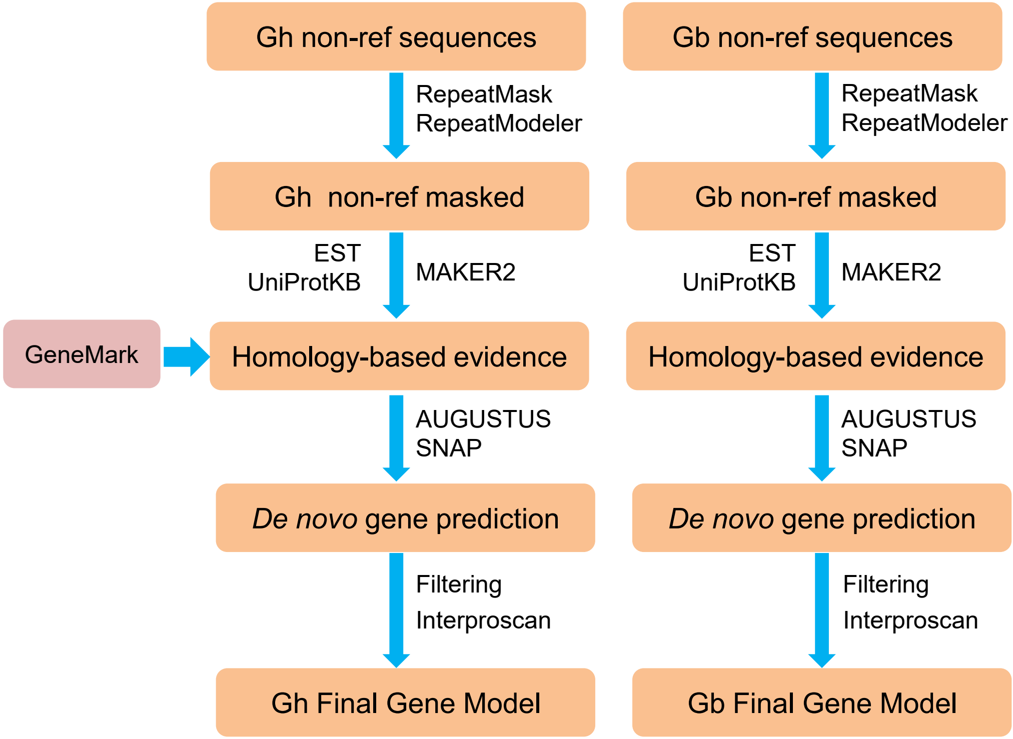

Figure S16

The protein coding gene prediction pipeline in the non-reference sequences.

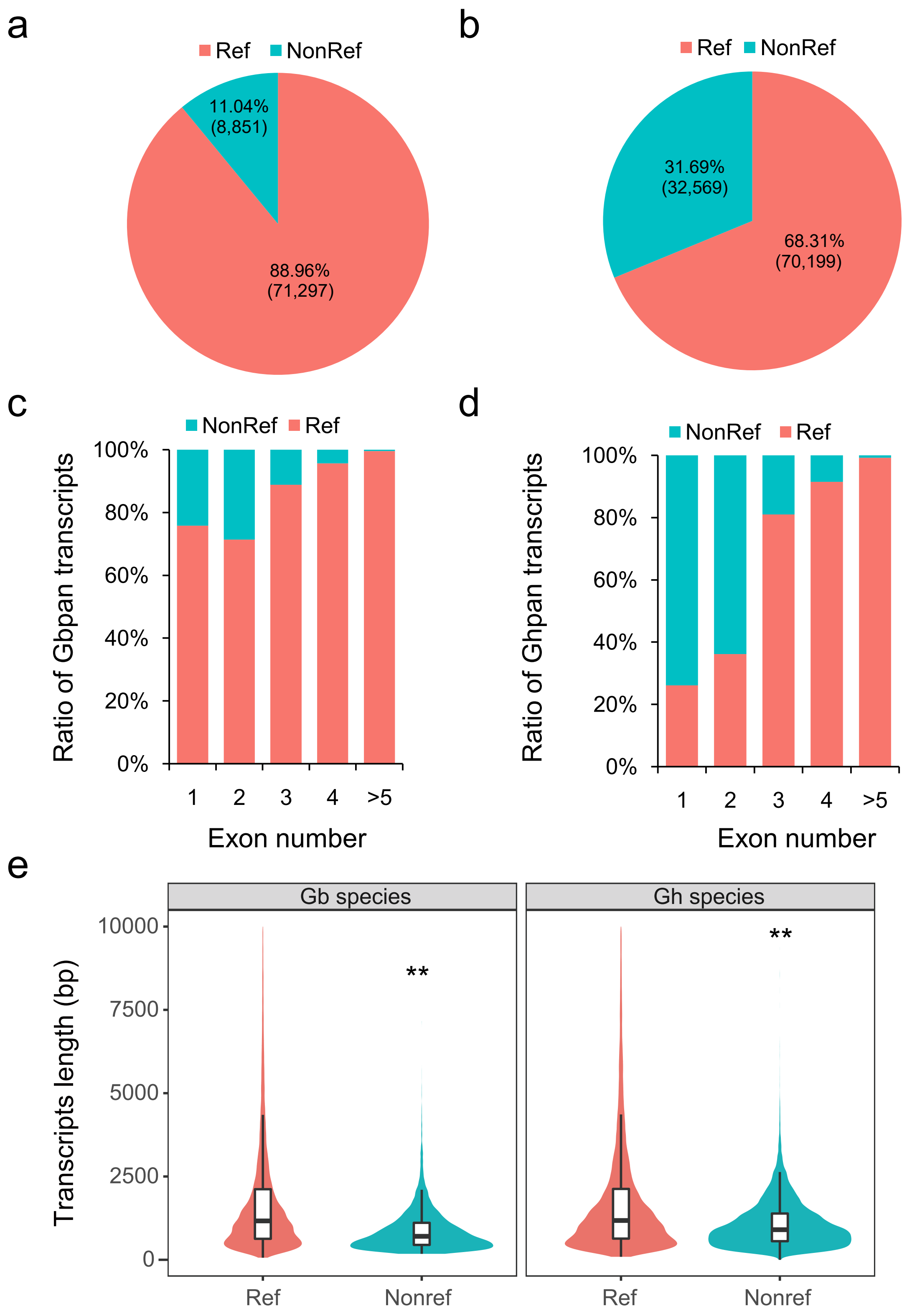

Figure S17

Comparison of reference genes and non-reference genes between *G. barbadense* and *G. hirsutum* pan-genomes. a-b Ratio of reference genes and non-reference genes between *G. barbadense* (**a**) and *G. hirsutum* (**b**). **c-d** Comparison of exon number between *G. barbadense* (**c**) and *G. hirsutum* (**d**) pan genes. **e** Transcript length of pan genes. The wilcoxon rank-sum test was used for significant analysis (**$P < 2.2\times10^{-16}$). Transcripts longer than 10 kb are not shown in this violin.

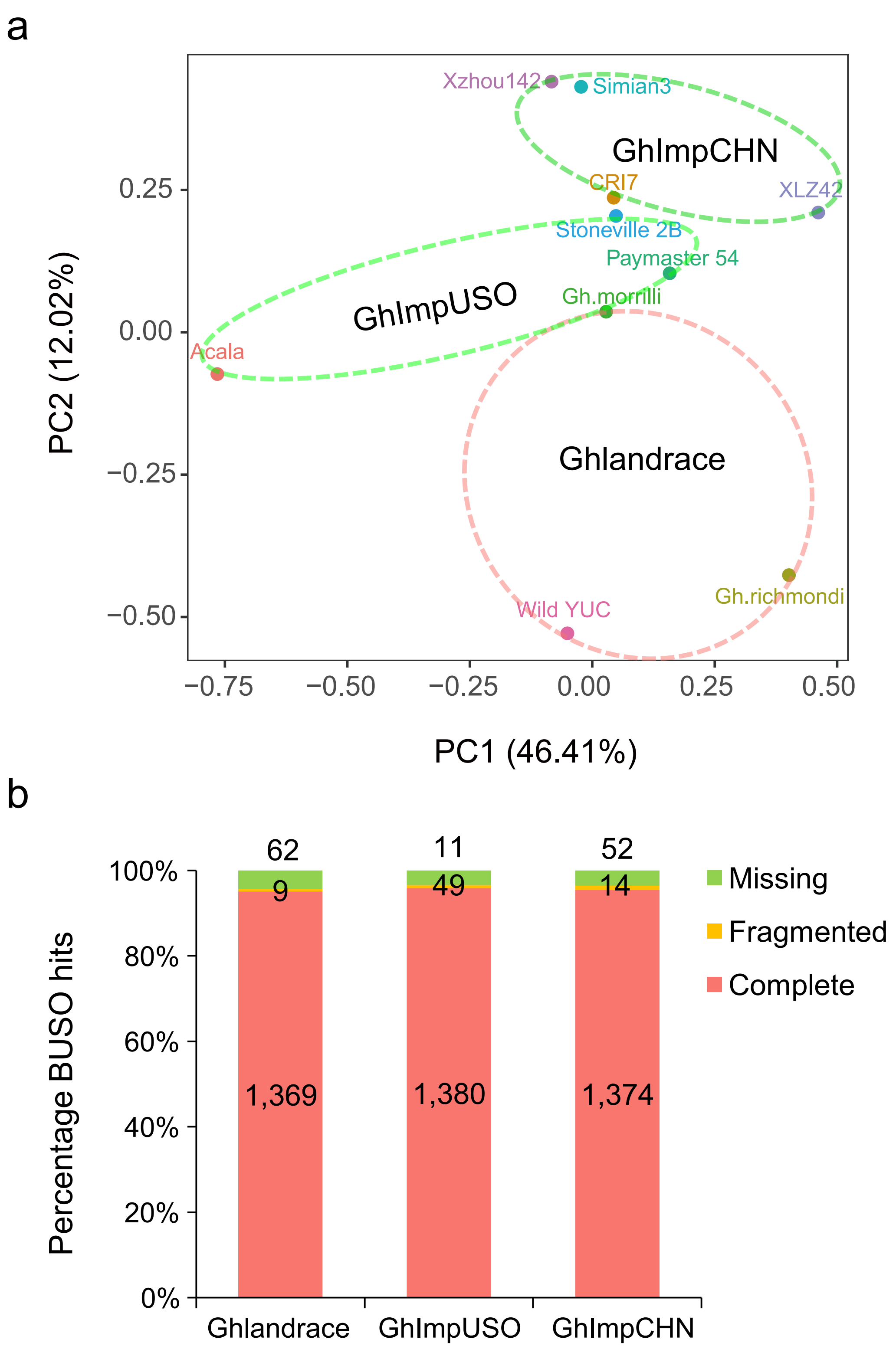

Figure S18

PCA and BUSCO analyses of 10 representative draft-genome assemblies. A PCA analysis of 10 accessions was based on the deletion variation. **b** Result of BUSCO analysis for contigs.

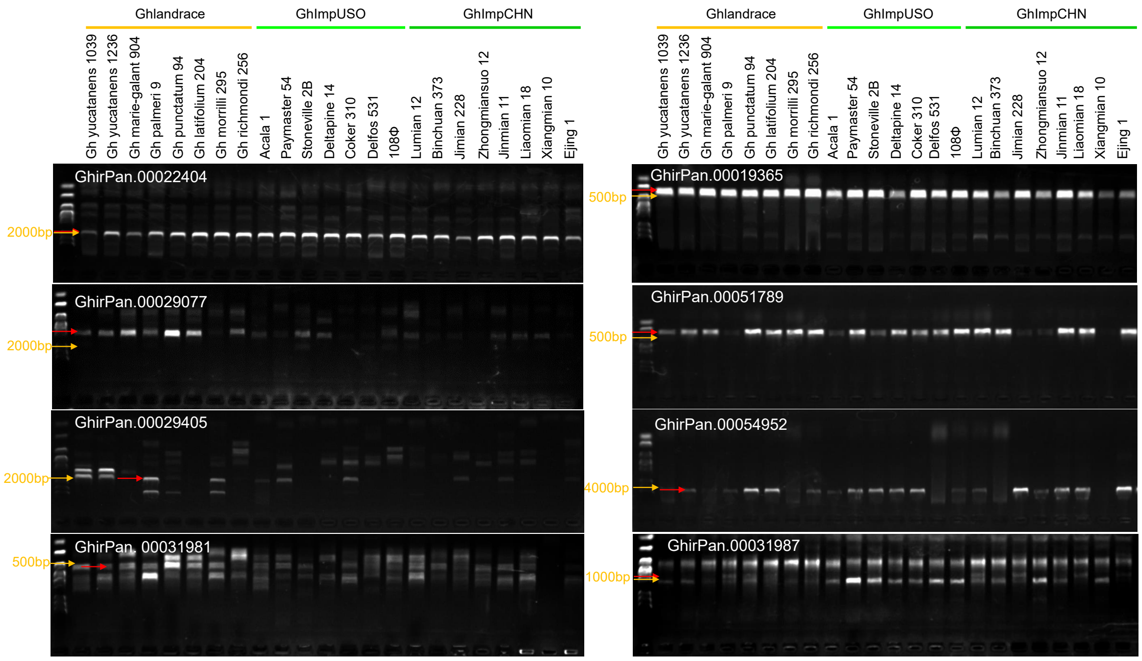

Figure S19

The eight random non-reference gene sequences were validated by PCR. For each gene, PCR product was amplified through 23 cotton accessions. The size of the PCR product was highlighted by red arrows. The size of DNA maker was highlighted by yellow arrows.

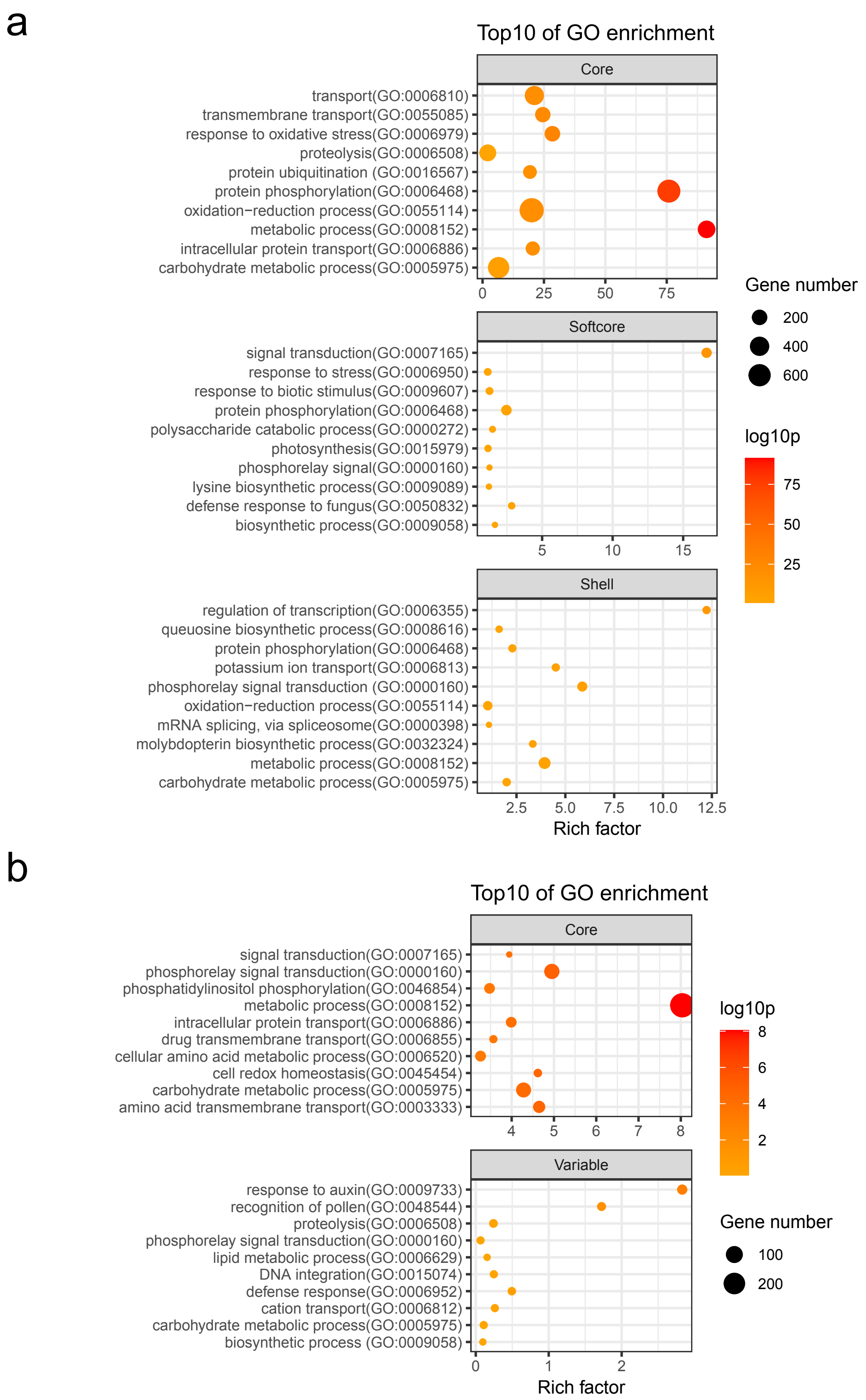

Figure S20

GO enrichment of core genes and variable genes in Ghpan-genome and Gbpan-genome. Percentage of biological process GO term for core genes and variable genes. The total number of genes in each GO term and significant level were calculated by Fisher's exact test. The 10 top GO term was shown. The top and bottom panel represent the *G. hirsutum* (**a**) and *G. barbadense* (**b**) pan-gene enrichment.

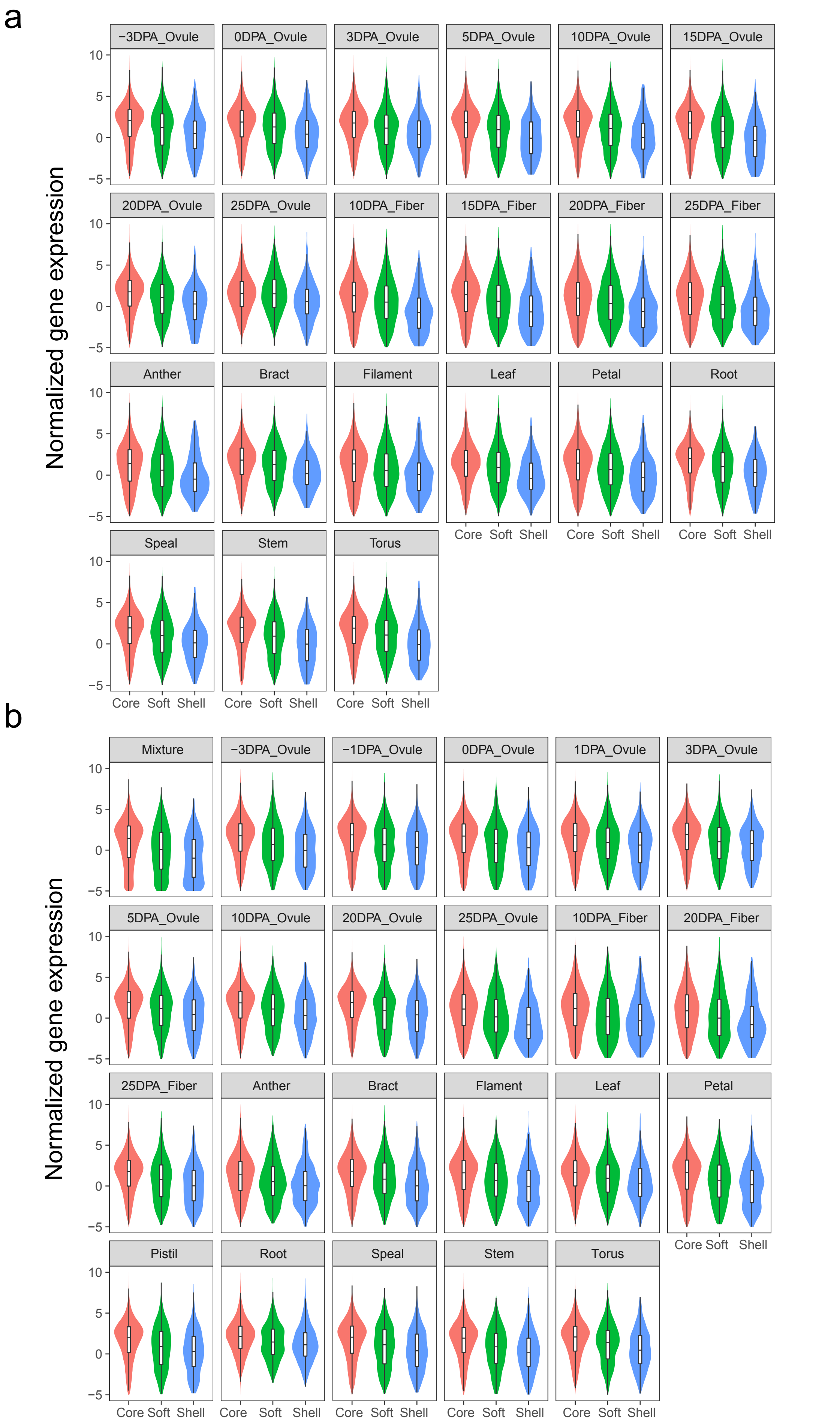

Figure S21

Expression levels of core, softcore and shell genes in multiple tissues of *G. hirsutum* (a) and *G. barbadense* (b). Core versus softcore and softcore versus shell comparisons are both significant ($P < 0.001$). This figure is related to **Fig. 4a.**

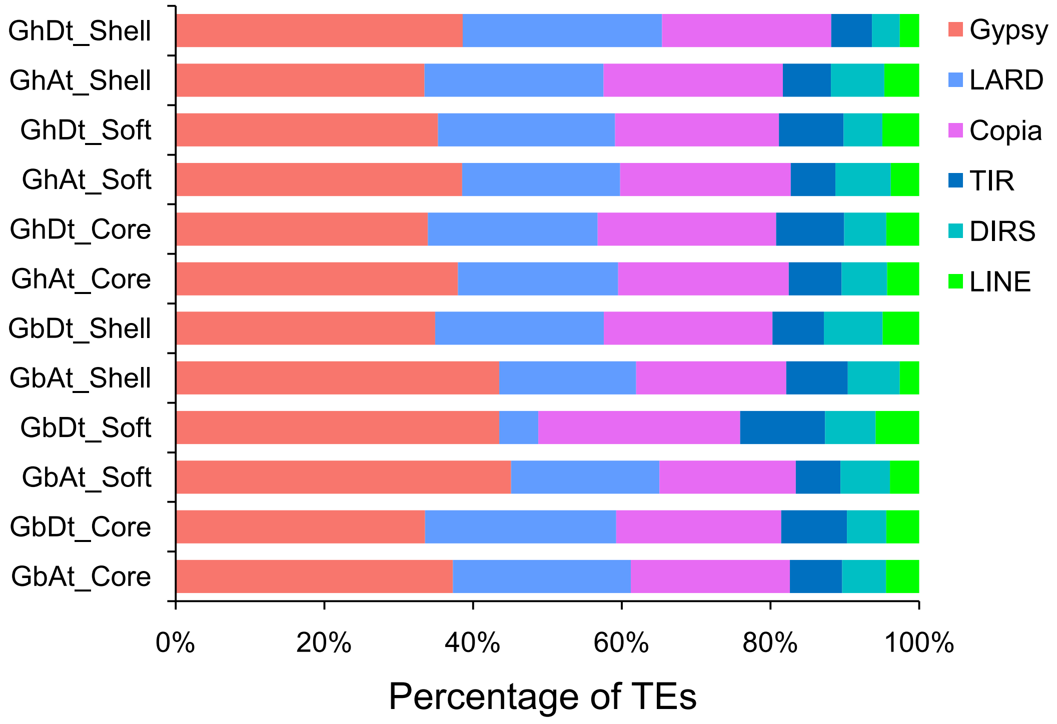

Figure S22

Ratio of *Gypsy*, *LARD*, *Copia*, *TIR*, *DIRS* and *LINE* transposons in upstream 2 kb of core and variable genes in the A- and D-subgenome.

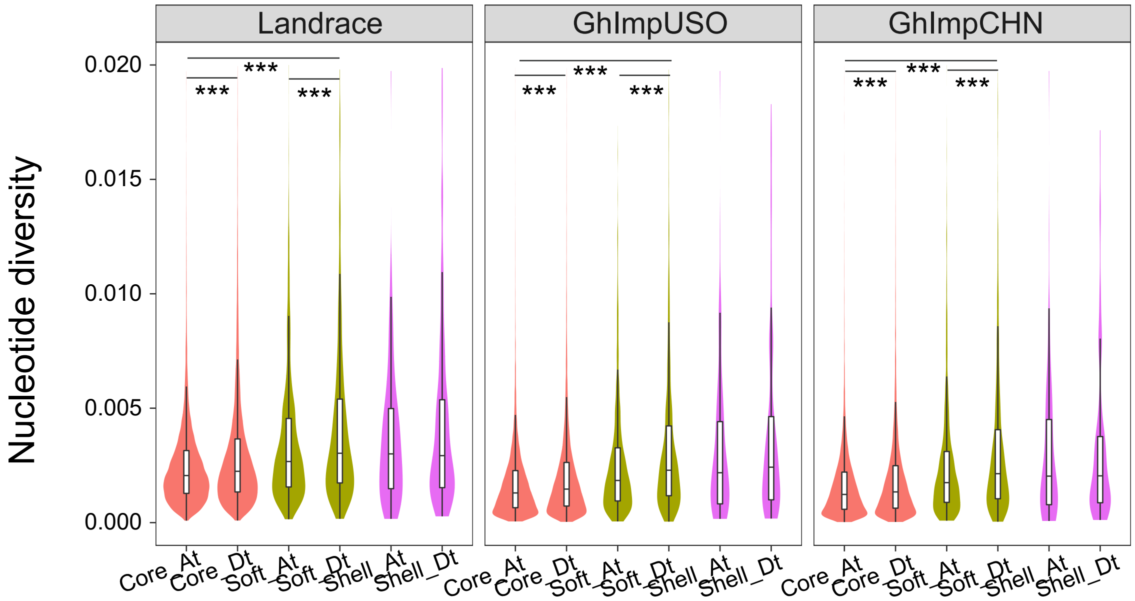

Figure S23

SNP diversity of core and variable genes for landrace, GhImpUSO and GhImpCHN population between the A and D subgenomes (Wilcoxon rank-sum test, *** *P* < 0.001). This figure is related to **Fig. 4d.**

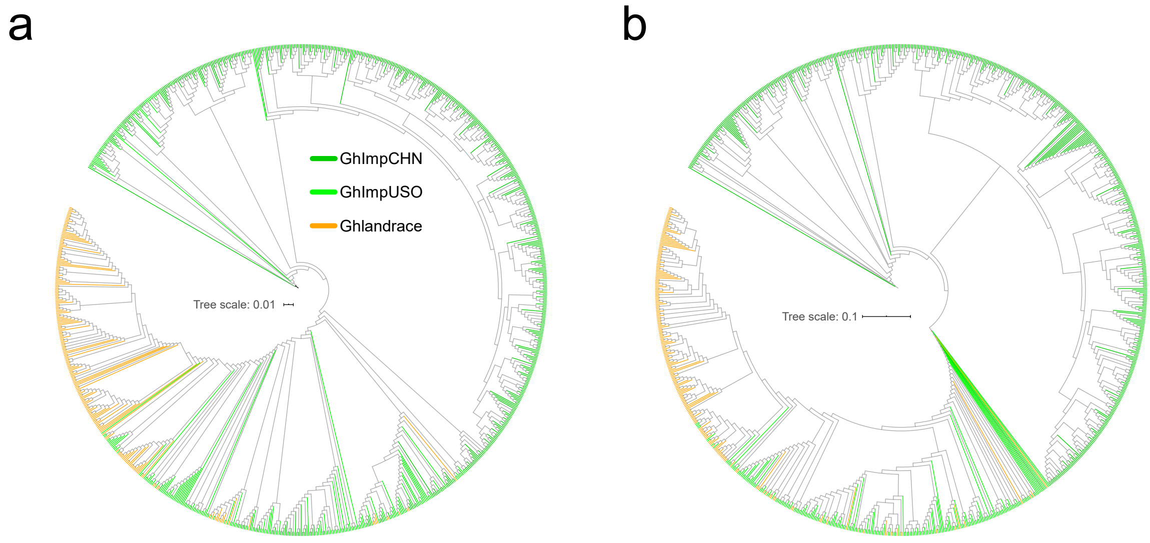

Figure S24

Phylogenetic tree of 1,020 *G. hirsutum* accessions based on 9,236,212 SNPs (a) and 3,806 shell PAVs (b).

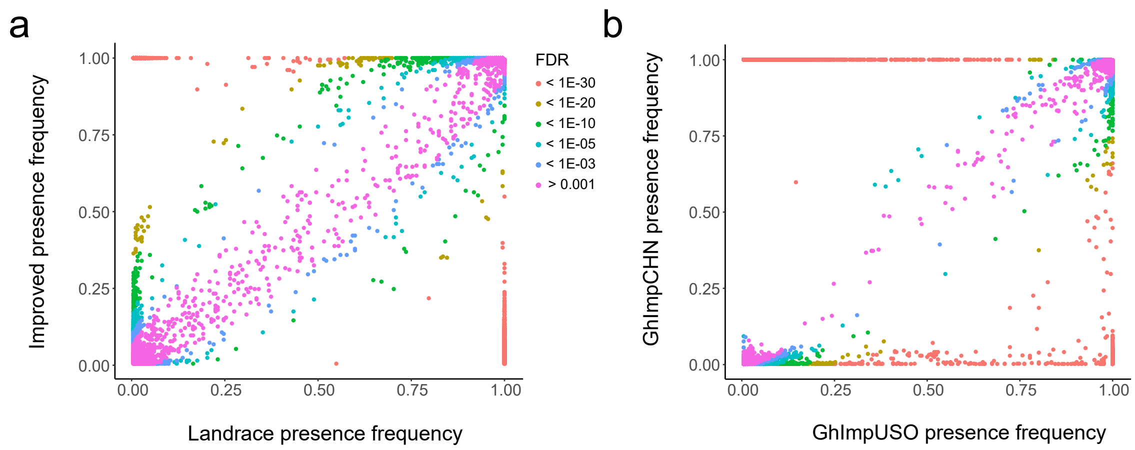

Figure S25

Comparison of presence frequency of variable genes during domestication and improvement. Scatter plots showing gene presence frequencies in Landrace and ImpUSO (**a**) and in ImpUSO and ImpCHN (**b**). Two-side Fisher's exact test was used for gene presence frequency significant in two groups. The *FDR* < 0.001 and frequency fold change > 2 for 'unfavorable gene' or < 0.5 for 'favorable gene' were regarded as selected genes.

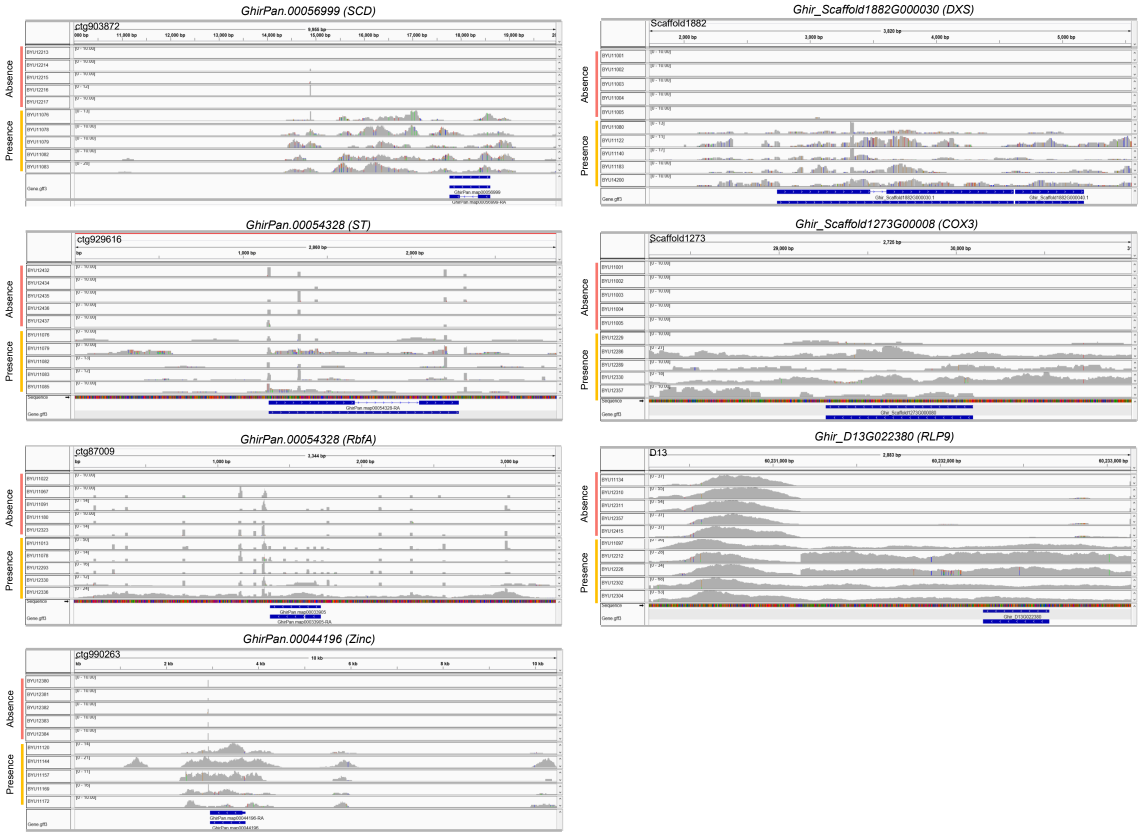

Figure S26

Genome alignment for seven PAVs and their flanking regions in representative accessions with presence and absence variations.

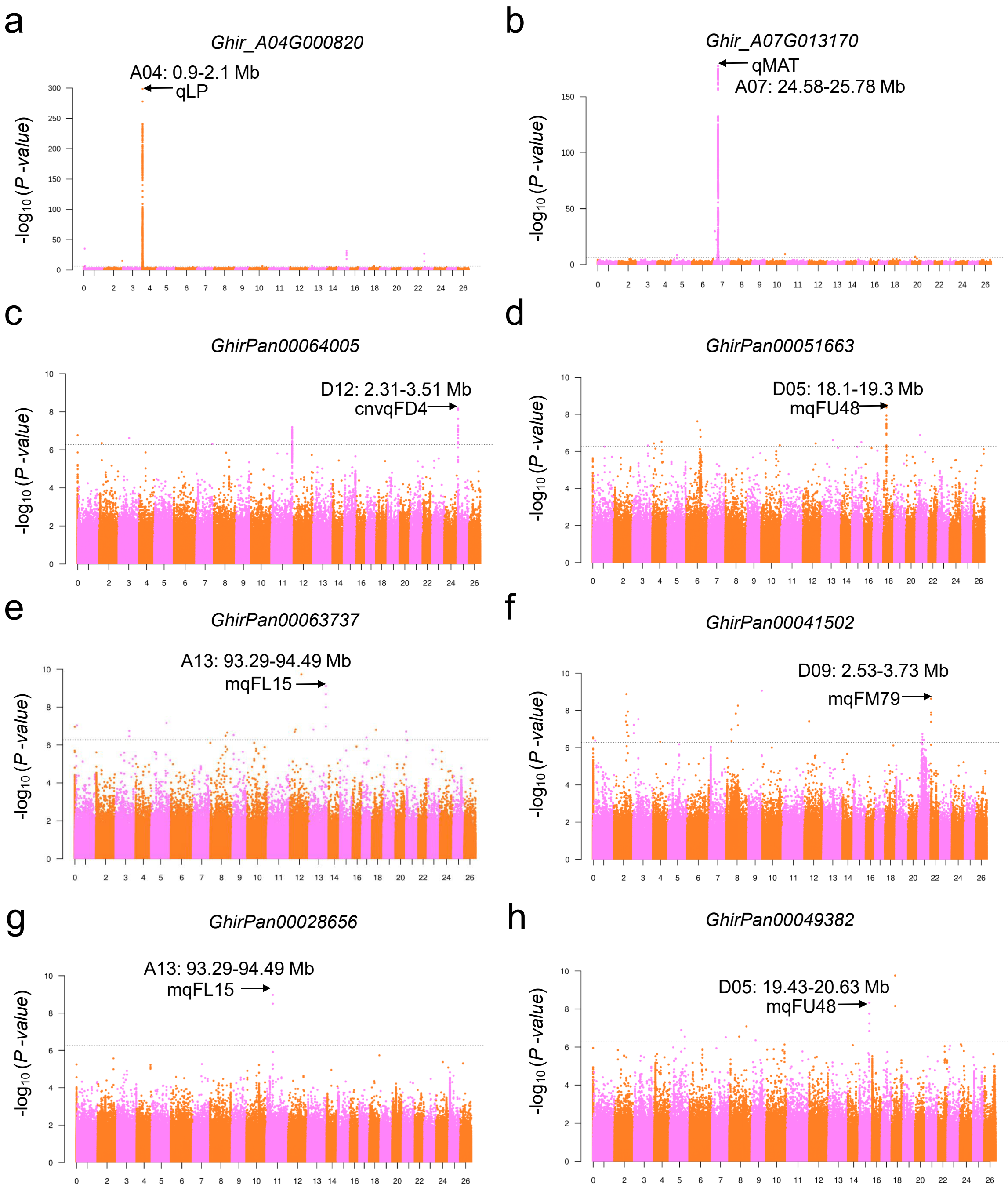

Figure S27

Example of PAVs-associated SNPs overlapping trait-QTLs. a-b Reference PAVs were associated with local QTLs**. c-h** the non-reference PAVs were associated with QTLs. PAVs are overlapped with trait-associated SNPs. The numbers of '1..26' represent the 'A01..D13' chromosomes. Seven fiber quality-related traits include fiber length (FL), fiber strength (FS), fiber micronaire (FM), fiber elongation (FE), fiber uniformity (FU), fiber maturity (MAT), spinning consistency index (SCI). Ten yield-related traits include boll weight (BW), lint percentage (LP), seed index (SI), lint index (LI), fiber weight per boll (FWPB), first fruit spur height (FFSH), seed-cotton weight (SW) and leafy shoot branch number (LBN). Two adaptation traits include whole growth period (WGP) and flowering date (FD). The PAV-associated QTL information was found in **Table S31.**

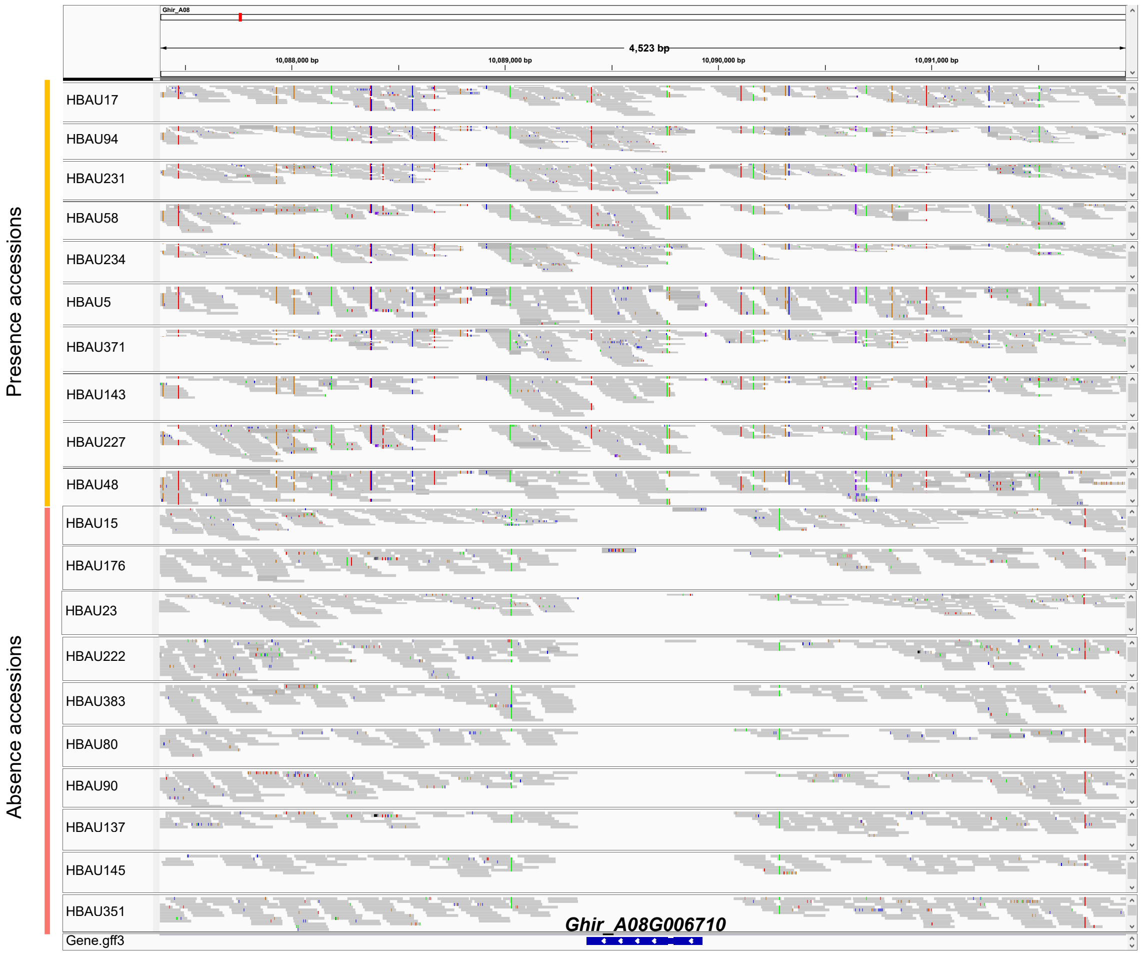

Figure S28

Genome alignment for the *Ghir_A08G006710* gene region in accessions with presence and absence variations. The upstream and downstream 2 kb region was shown.

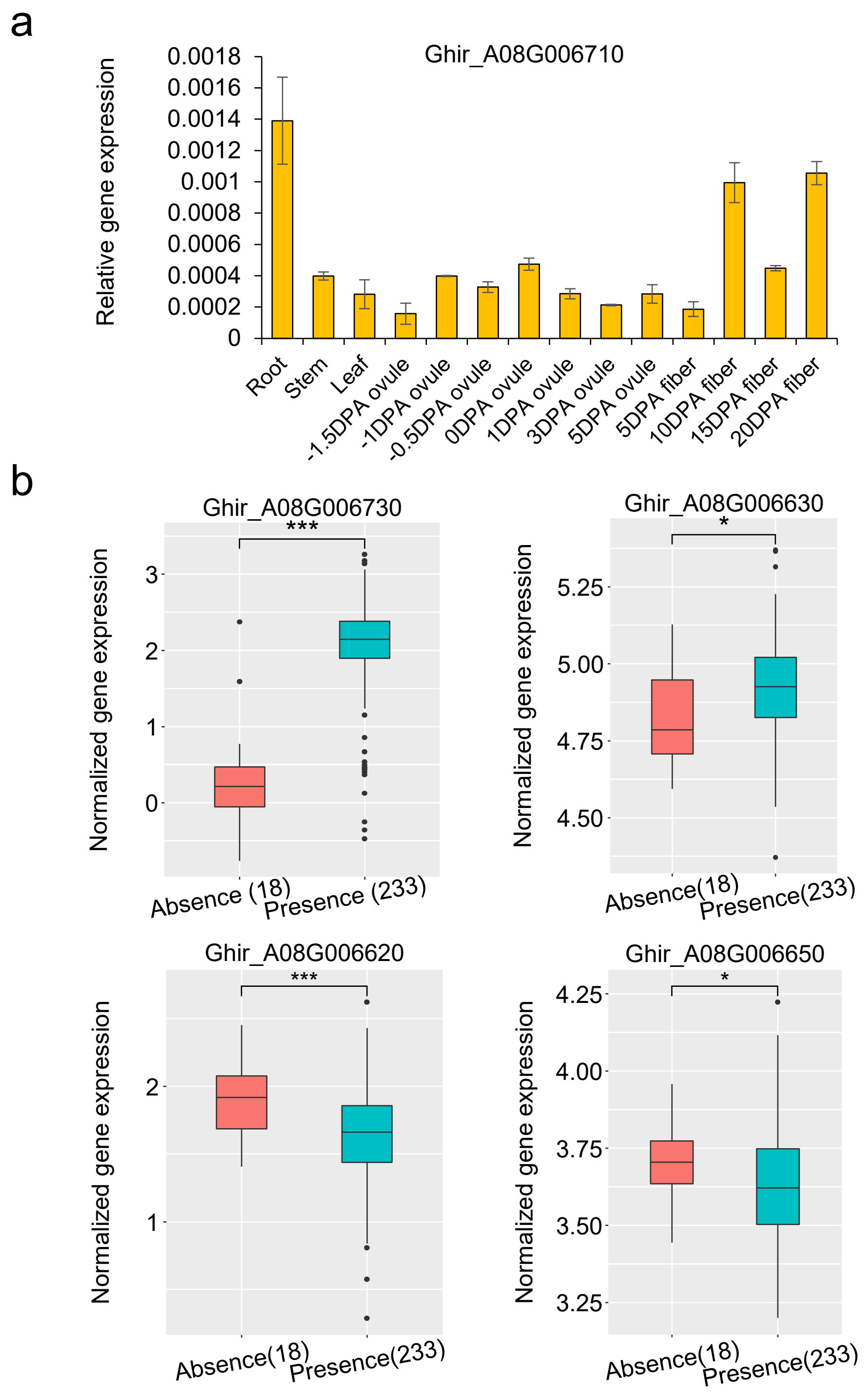

Figure S29

The *Ghir_A08G006710* gene expression and adjacent gene expression. **a** Quantitative real -time PCR (qPCR) analysis of *Ghir_A08G006710* in 14 cotton tissues of TM-1 cultivar. The *GhUB7* was used as the reference gene. The standard error bars were calculated from three biological replicates . **b** The flanking gene expression at the 15 DPA fibers of *Ghir_A08G006710* in 251 cotton accessions. The gene expression data were from 251 cotton accessions (Li et al., 2020; 226:1738-1752). This gene was absent in 18 accessions (Absence (18)) and was present in 233 accessions (Presence (233)). Gene expression of the flanking 200 Kb region (17 genes) was analyzed, of which 4 genes were differentially expressed between absence and presence haplotypes. Days post-anthesis (DPA).

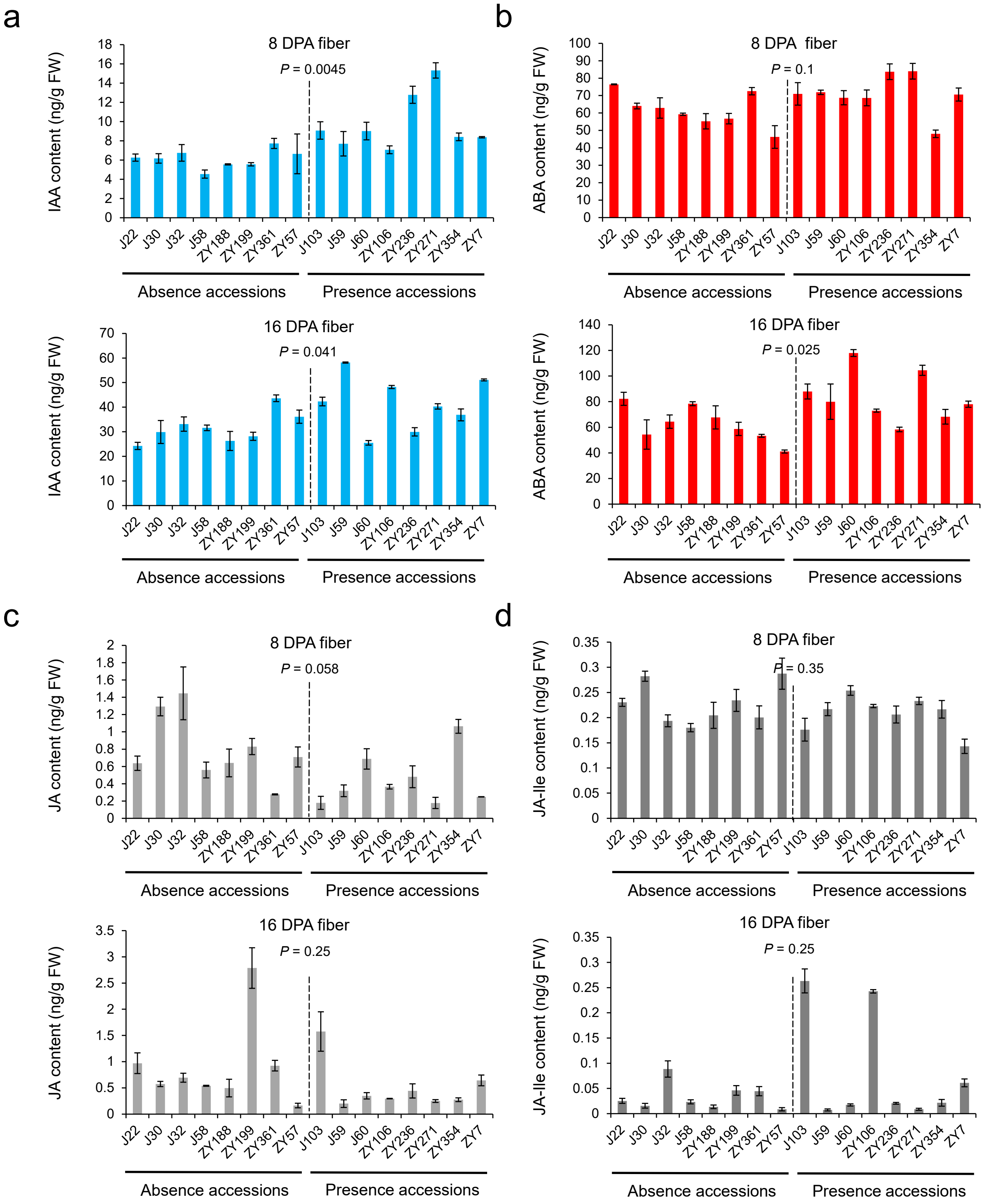

Figure S30

The content of indole-3-acetic acid (IAA), abscisic acid (ABA), jasmonic acid (JA) and jasmonoyl-isoleucine (JA-Ile) were measured in 16 accessions with the presence and absence haplotypes at 8 DPA and 16 DPA cotton fibers. Each sample was performed with three biological replicates. Two-sided student's t-test was used for significance analysis between absence and presence of haplotypes.

References

1. Yoo MJ, Wendel JF. Comparative evolutionary and developmental dynamics of the cotton (*Gossypium hirsutum*) fiber transcriptome. PLoS Genet. 2014;10:e1004073.
2. Wang M, Tu L, Lin M, Lin Z, Wang P, Yang Q, Ye Z, Shen C, Li J, Zhang L, et al. Asymmetric subgenome selection and cis-regulatory divergence during cotton domestication. Nat Genet. 2017;49:579-587.
3. Ma Z, He S, Wang X, Sun J, Zhang Y, Zhang G, Wu L, Li Z, Liu Z, Sun G, et al. Resequencing a core collection of upland cotton identifies genomic variation and loci influencing fiber quality and yield. Nat Genet. 2018;50:803-813.
4. Huang C, Nie X, Shen C, You C, Li W, Zhao W, Zhang X, Lin Z. Population structure and genetic basis of the agronomic traits of upland cotton in China revealed by a genome-wide association study using high-density SNPs. Plant Biotechnol J. 2017; 15:1374-1386.
5. Fang L, Wang Q, Hu Y, Jia Y, Chen J, Liu B, Zhang Z, Guan X, Chen S, Zhou B, et al. Genomic analyses in cotton identify signatures of selection and loci associated with fiber quality and yield traits. Nat Genet. 2017;49:1089-1098.
6. Li Z, Wang P, You C, Yu J, Zhang X, Yan F, Ye Z, Shen C, Li B, Guo K, et al. Combined GWAS and eQTL analysis uncovers a genetic regulatory network orchestrating the initiation of secondary cell wall development in cotton. New Phytol. 2020;226:1738-1752.
