# Supplementary material for: Cotton pan-genome retrieves the lost sequences and genes during domestication and selection
Source: Genome Biol. 2021 Apr 23;22:119. doi: 10.1186/s13059-021-02351-w (PMC8063427; doi:10.1186/s13059-021-02351-w)
Supplement: Supplementary file 3 — Additional file 3. Supplementary Notes. [file 13059_2021_2351_MOESM3_ESM.doc]

**Supporting Notes**

**Cotton pan-genome retrieves the lost sequences and genes during domestication and selection**

Jianying Li^1^, Daojun Yuan^2^, Pengcheng Wang^1^, Qiongqiong Wang^1^, Mengling Sun^1^, Zhenping Liu^1^, Huan Si^1^, Zhongping Xu^1^, Yizan Ma^1^, Boyang Zhang^1^, Liuling Pei^1^, Lili Tu^1^, Longfu Zhu^1^, Ling-Ling Chen^3^, Keith Lindsey^4^, Xianlong Zhang^1^, Shuangxia Jin^1*^ and Maojun Wang^1*^

^1^National Key Laboratory of Crop Genetic Improvement, Huazhong Agricultural University, Wuhan, China

^2^College of Plant Science and Technology, Huazhong Agricultural University, Wuhan, China

^3^Hubei Key Laboratory of Agricultural Bioinformatics, College of Informatics, Huazhong Agricultural University, Wuhan, China

^4^Department of Biosciences, Durham University, Durham, UK

E-mail: [mjwang@mail.hzau.edu.cn](mailto:mjwang@mail.hzau.edu.cn); [jsx@mail.hzau.edu.cn](mailto:jsx@mail.hzau.edu.cn)

**Supplementary Notes**

**1. Sample collection and SNP/InDel variation filtering**

DNA sequencing data of 1,913 cottons for the SNP and InDel variation analysis, which include 261 *G. barbadense* cultivars with a depth of 1-52 × (average 21.8 ×), 256 *G. hirsutum* landraces (Ghlandrace) with a depth of 3-37.6 × (average 25.8 ×), 438 cultivars from American and other countries (GhImpUSO) with a depth of 1-57 × (average 18.2 ×), 929 China modern improved cultivars (GhImpCHN) with a depth of 1-76 × (average 7.1 ×) and 29 other *Gossypium* species ((AD)_3_-(AD)_7_) with a depth of 4-44× (average 22.9 ×) (**Additional file 1: Table S1**). For the China germplasm collection, this population includes 315 accessions from the Yellow River Region (YRR), 236 accessions from the Yangtze River Region (YtRR), 117 accessions from the Northwestern Inland Region (NIR), 60 accessions from the Northern Specific Early Maturation Region (NSEMR), 27 accessions from the Southern China Region (SCR) and 174 accessions from other regions.

After imputation of SNP dataset, a total of 119,678,187 and 60,919,990 bi-allele SNPs based on the 'TM-1' reference genome (discarding SNPs in the scaffold sequences) was retained in 1,913 and 1,623 allotetraploid cotton accessions, respectively (**Additional file 1: Tables S2 and S4**). The filtered set of 63,084,975 and 34,288,482 SNPs was further filtered with parameters of minor allele frequencies (MAF) greater than 0.001, with missing rate less than 80% in 1,913 and 1,623 population. In order to exclude putative assembly errors in reference genomes, we discarded the variation with MAF greater than 99% in each specific population. The 25,322,823 and 12,548,837 high-quality SNPs were obtained with MAF ≥ 1% and ≤ 99% in populations with 1,913 and 1,623 accessions, respectively. Finally, the 19,246,497 and 9,546,745 core SNPs were obtained with criteria of at least five accessions having homozygous loci. At the same time, the 261 *G. barbadense* accessions have 8,645,255 high-confident SNPs and 731,641 InDels against the '3-79' reference genome (**Additional file 1: Table S5**).

**2. Sample selection for SV analysis**

The 742 cottons were used for SV and CNV variation analysis. The 742 accessions included 693 accessions (sequencing depth > 10×) of 1,913 population and 49 accessions in other SRA projects (PRJNA321738, PRJNA450479, PRJNA576032) were also used for SV population (**Additional file 1: Table S1**). To control the false positive rate of SV identification in 742 cottons, we selected 10 diploid species (two *Gossypium arboretum* (A2), one *Gossypium davidsonii* (D3), two *Gossypium gossypioides* (D6), two *Gossypium anomalum* (B1), three *Gossypium exiguum* (K1) genomes) with high sequencing depth as the first outgroup, 19 tetraploid *Gossypium* species (6 *Gossypium tomentosum* (AD3) cottons, 4 *Gossypium mustelinum* (AD4) cottons, 4 *Gossypium darwinii* (AD5) cottons, 6 *Gossypium ekmanianum* (AD6) cottons and one *Gossypium stephensii* (AD7)) as the second outgroup, 192 *G. barbadense* accessions, and 521 *G. hirsutum* accessions (251 Ghlandraces, 240 GhImpUSO accessions, 30 GhImpCHN accessions). Combined with these outgroup, population structure can be clearly separated with *G. hirsutum* and *G. barbadense* species (Figure S4e). According to CNVcaller instructions, the 419 accessions resequencing data (with a depth of more than 5) from Hebei Agricultural University (HBAU) [3] were also used for CNV calling.

**3. Multiple panel GWAS loci filtering**

In this study, 267 accessions from Huazhong Agricultural University (HZAU) [1], 258 accessions from Nanjing Agricultural University (NJAU) [2], and 419 accessions from Hebei Agricultural University (HBAU) [3] were used for GWAS analysis, respectively. There are 44 overlapping accessions in the NJAU and HBAU accessions. After discarding missing phenotype and poor-quality genotyping data (10 low sequenced accessions in NJAU, depth < 1×), 207 accessions were collected in NJAU, 264 in HZAU and 419 in HBAU for GWAS analysis. The broad-sense heritability and best linear unbiased predictions (BLUPs) [4] breeding values were calculated across different environments. In total, fiber quality BLUP values of 890 non-redundant accessions from three independent experiments were used for meta-GWAS. Meta-GWAS was performed for fiber length (FL), fiber strength (FS), fiber micronaire (FM), fiber elongation (FE) and fiber uniformity (FU). The heritability values of FL, FS, FU, FM, FE were 0.93, 0.92, 0.80, 0.81 and 0.69, respectively. GWAS significant hits were obtained using the TASSEL5.0 [5] with a mixed linear (P + G + Q + K) model and FastLMM [6] program. The sharp peaks of continuous significant SNPs were screened as snpQTLs in meta-GWAS analysis and the random single peaks were discarded (**Additional file 1: Table S14**). Meanwhile, three independent case GWAS were performed in multiple environments, in which significant SNPs detected in two environments were also treated as QTLs. The significant thresholds were set as 0.05/N and 1/N in Meta-GWAS and Case-GWAS, respectively ("N" represents the total number of SNPs). Significant SNPs located upstream and downstream of 600 kb (GhImpCHN group LD decay is 587 kb; flanking 600 kb) sequences were considered as a QTL. CNVs-based GWAS was performed using 26,831 CNVs for 13 agronomic traits in the 419 population. The CNV-based QTLs (GhImpCHN group LD decay is 655 kb in CNV-level; selection flanking 1 Mb) were detected in at least two environments. The same overlapping QTL regions from different agronomic traits were regarded as pleiotropic QTLs.

**4. *De novo* assembly of short reads and filtering of non-reference sequences**

In total, the 1,581 *G. hirsutum* and 226 *G. barbadense* cotton accessions were subject to *de novo* assembly. First, SAMtools was used to extract unmapped reads, including the single unmapped reads with '-b -f 4' and paired unmapped reads '-f 68 -F 8' and '-f 132 -F 8' parameters [7]. These unmapped reads were initially assembled with 'PE = 300 50' and 'LIMIT_JUMP_COVERAGE = 300' for each accession. After filtering of contigs with a length < 500 bp and discarding outlier samples, we obtained 1,138 Mb, 1,241 Mb, 1,819 Mb, 1,422 Mb for 251 Ghlandrace, 424 GhImpUSO, 906 GhImpCHN accessions, and 226 *G. barbadense* cultivars. After discarding redundant sequences using CD-HIT [8], we obtained 1,084 Mb, 1,750 Mb, 1,183 Mb, and 1,356 Mb with contig N50 of 1,526 bp, 1,299 bp, 1,367 bp, 1,108 bp in Ghlandrace, GhImpUSO, GhImpCHN and *G. barbadense* groups, respectively (**Additional file 1: Table S19**). A total of 3,704 Mb in 1,581 *G. hirsutum* and 1,422 Mb in 226 *G. barbadense* were used for the following filtering steps. The *G. hirsutum* non-reference sequences were aligned to 'TM-1' reference genome, *G. barbadense* non-reference sequences were aligned to'3-79' reference genome [9,10] and plasmid genomes were used to identify *bone fide* non-reference sequences, which resulted in 3,612 Mb in *G. hirsutum* and 1,064 Mb in *G. barbadense.* After filtering all-by-all alignments, we found that 1,806 Mb and 692 Mb non-redundant sequences in *G. hirsutum* and *G. barbadense* were absent in 'TM-1' and '3-79' reference genomes, respectively (**Additional file 1: Table S20**). Following these filtering steps, the retained non-reference sequences with a length of more than 1000 bp were used to predict protein coding genes (**Additional file 2: Figure S14)**.

**5. *De novo* assembly of 10 representative accessions**

We selected 10 representative cotton accessions in the 1,625 *G. hirsutum* population. The PacBio Sequel II platform was used to generate 28,289,938 subreads for 10 accessions, with a total length of 286 Gb. The average subread N50 of each accession is 15 kb with an average depth of 11.9×. The 25,059,382 subreads were filtered (length < 1 Kb). The Illumina platform generated 4,667,133,522 paired-end reads (60×; **Table S23**). Two strategies were used for genotyping of structural variation based on PacBio and Illumina reads. First, PacBio reads were aligned using NGMLR [11] (v0.2.6) tool to detect SVs. The second SV genotyping method is based on sequence assembly. Long reads were assembled using the MECAT [12] tool following steps: (1) PacBio reads were used to detect overlapping candidates using mecat2pw. (2) Pairwise overlapping candidates were corrected using mecat2cns. (3) Longest corrected reads covering at least 15× were extracted and mecat2canu (ErrorRate = 0.02) was used for genome assembly. The long reads were aligned to assembly contigs using the BLASR (v 5.3), which can check supporting mapping rate. Then, the Illumina pair-end reads were aligned against the contigs using BWA (v 0.7.10-r789) and were then polished using the pilon (v 1.23) program [13]. After two rounds of polishing, 2,550,224 SNPs, 13,154,090 insertions and 2,151,774 deletions were corrected on average for each cotton accession. Meanwhile, semi-wild landraces, American cottons and China cultivated modern accessions were assembled through a meta-genome likely strategy [14]. First, we merged PacBio reads from Acala, Paymaster 54, Stoneville 2B in the American group as 'GhImpUSO', Simian 3, CRI 7, Xinluzao 42, Xuzhou 142 from China improved modern cultivars as 'GhImpCHN', the *G. hirsutum richmondi*, *G. hirsutum morrilli*, *G. hirsutum yucatanense* from the semi-wild cottons as 'Ghlandrace' (**Additional file 1: Table S25**). Then, the merged reads were assembled by MECAT and contigs were polished using Illumina reads.

References

1. Wang M, Tu L, Lin M, Lin Z, Wang P, Yang Q, Ye Z, Shen C, Li J, Zhang L, et al. Asymmetric subgenome selection and cis-regulatory divergence during cotton domestication. Nat Genet. 2017;49:579-587.

2. Fang L, Wang Q, Hu Y, Jia Y, Chen J, Liu B, Zhang Z, Guan X, Chen S, Zhou B, et al. Genomic analyses in cotton identify signatures of selection and loci associated with fiber quality and yield traits. Nat Genet. 2017;49:1089-1098.

3. Ma Z, He S, Wang X, Sun J, Zhang Y, Zhang G, Wu L, Li Z, Liu Z, Sun G, et al. Resequencing a core collection of upland cotton identifies genomic variation and loci influencing fiber quality and yield. Nat Genet. 2018;50:803-813.

4. Poland JA, Bradbury PJ, Buckler ES, Nelson RJ. Genome-wide nested association mapping of quantitative resistance to northern leaf blight in maize. Proc Natl Acad Sci U S A. 2011;108:6893-6898.

5. Bradbury PJ, Zhang Z, Kroon DE, Casstevens TM, Ramdoss Y, Buckler ES. TASSEL: software for association mapping of complex traits in diverse samples. Bioinformatics. 2007;23:2633-2635.

6. Lippert C, Xiang J, Horta D, Widmer C, Kadie C, Heckerman D, Listgarten J. Greater power and computational efficiency for kernel-based association testing of sets of genetic variants. Bioinformatics. 2014;30:3206-3214.

7. Li H, Handsaker B, Wysoker A, Fennell T, Ruan J. The Sequence Alignment-Map format and SAMtools. Bioinformatics. 2009;25:2078-2079.

8. Fu L, Niu B, Zhu Z, Wu S, Li W. CD-HIT: accelerated for clustering the next-generation sequencing data. Bioinformatics. 2012;28:3150-3152.

9. Wang M, Tu L, Yuan D, Zhu, Shen C, Li J, Liu F, Pei L, Wang P, Zhao G, et al. Reference genome sequences of two cultivated allotetraploid cottons, *Gossypium hirsutum* and *Gossypium barbadense*. Nat Genet. 2019;51:224-229.

10. Hu Y, Chen J, Fang L, Zhang Z, Ma W, Niu Y, Ju L, Deng J, Zhao T, Lian J, et al. *Gossypium barbadense* and *Gossypium hirsutum* genomes provide insights into the origin and evolution of allotetraploid cotton. Nat Genet*.* 2019;51:739-748.

11. Sedlazeck FJ, Rescheneder P, Smolka M, Fang H, Nattestad M, von Haeseler A, Schatz MC: Accurate detection of complex structural variations using single-molecule sequencing. Nat Methods. 2018;15:461-468.

12. Xiao CL, Chen Y, Xie SQ, Chen KN, Wang Y, Han Y, Luo F, Xie Z. MECAT: fast mapping, error correction, and de novo assembly for single-molecule sequencing reads. Nat Methods. 2017;14:1072-1074.

13. Walker BJ, Abeel T, Shea T, Priest M, Abouelliel A, Sakthikumar S, Cuomo CA, Zeng Q, Wortman J, Young SK, Earl AM. Pilon: an integrated tool for comprehensive microbial variant detection and genome assembly improvement. PLoS One. 2014;9:e112963.

14. Yao W, Li G, Zhao H, Wang G, Lian X, Xie W. Exploring the rice dispensable genome using a metagenome-like assembly strategy. Genome Biol. 2015;16:187.
