# Supplementary material for: Cotton pan-genome retrieves the lost sequences and genes during domestication and selection
Source: Genome Biol. 2021 Apr 23;22:119. doi: 10.1186/s13059-021-02351-w (PMC8063427; doi:10.1186/s13059-021-02351-w)
Supplement: Supplementary file 4 — Additional file 4. Review history. [file 13059_2021_2351_MOESM4_ESM.docx]

Review History

**First round of review**

**Reviewer 1**

**Are you able to assess all statistics in the manuscript, including the appropriateness of statistical tests used?**

Yes, and I have assessed the statistics in my report.

**Comments to author:**

In their paper, Li et al. assemble a cotton pangenome using 1,913 accessions for two of the allopolyploid cottons Gossypium hirsutum and G. barbadense using the G. hirsutum reference genome. The authors map deletions associated with domestication, call SNPs and CNVs and run genome-wide association studies with those around traits of agronomic interest,

The outline and figure structure mostly follows the tomato pangenome (Gao et al., 2019, The tomato pan-genome uncovers new genes and a rare allele regulating fruit flavor), I can't find any major issues there, it's just not that novel in terms of findings. It should be interesting to people working in the cotton space, though

Some minor things:

- I think the paper's introduction would explain the pangenome concept better to readers outside of the field if one or two of the [18-31] citations (line 69) would be summarised for the reader

- line 108: 'found identify 32,099 deletions' should probably be 'identified' or 'found' alone

- line 133, 'including 274 homologous genepairs' - should that be 'homeologous gene pairs' as these seem to be between the two subgenomes? Same in line 140. I may misunderstand this point as the methods do not state how the gene pairs were calculated, that needs to be added. I'm guessing MCScanX?

- line 136, 'circadian rhythm process for fiber elongation and environmental adaptation' - this sounds like the circadian rhythm is involved in fiber elongation, is that true? Similarly, I don't see anything directly about environmental adaptation in Suppl. Figure 7. Perhaps that should be rewritten

- line 206, the citation for the 'reference-guided approach' points to the sunflower genome, but it should point to reference 21, Golicz et al., B. oleracea pangenome since that paper pioneered this approach in plants

- line 231, it says that 18.9 Mb of unmapped contigs were aligned, it would be helpful to have the percentage of the total size of all unmapped contigs here

- line 340, it says 'For genes favorable to cotton breeding,' - this sounds like these genes are helpful in the process of breeding itself, while in reality, they were selected for during domestication, either during random bottleneck events or because they confer some trait of interest to breeders. The word should be changed

- line 556, 'The best contig alignments against higher plants were considered as contaminant.' I'm guessing this should be NOT against higher plants, removing plants as contaminants would not be good

- line 557, this sentence is a bit confusing to me: 'The Ghpan-genome and Gbpan-genome were generated by combining the 'TM-1' and '3-79' reference genome and respective non-redundant sequences.' However, the earlier text (line 92, 108) seem to imply that only TM-1 was used, while line 209 implies that the two genomes were used separately as reference to make *two* pangenomes. The authors should clarify which approach was used.

- line 590, I don't understand this sentence; 'The final transcripts were aligned using interproscan [] and added to the final gene models'. Interproscan is normally used to search protein sequences for domains while this sentence implies that interproscan was used to align the gene models with something, but with what?

**Reviewer 2**

**Are you able to assess all statistics in the manuscript, including the appropriateness of statistical tests used?**

Yes, and I have assessed the statistics in my report.

**Comments to author:**

Recent cotton genome advances greatly drove cotton population research and it is the best time to perform structural variations among populations and pan-genome analysis. In this manuscript, Li et al. collected 1,913 cotton accessions to do cotton genomic variome and identified lost sequences and genes during population development. Obviously, the work will be a great genetic resource for cotton community. I applaud for their idea and the great effort the researchers put in to generate this resource.

This paper is globally interesting. However, I do think the weakest point of this manuscript is the lack of functional analysis of their novel QTLs/PAVs. Although they retrieved the lost sequences and genes during domestication and selection, they have no direct evidences to support that these sequences and genes were biologically important. All of their analysis in the manuscript rely totally on the association between CNVs/SVs/PAV and previously reported QTLs, which seems very rudimentary and is difficult to determine whether their identified SVs, CNV, PAV, or QTLs were biologically important and whether their novel QTLs were the major functional sites, so I do think the authors need to confirm the biological functions of at least one or two novel identified QTLs by transgenic cotton experiments (Overexpression, RNAi or genome editing system) (see my major concerns #1, 2, and 3). In summary, the manuscript has to go through a thorough revision with several new experiments before it can be considered for publication in Genome Biology!

Major concerns:

1. For Figure 2b-i, the authors identified novel QTLs based-on CNVs and listed several examples for SI, FL, BW, FU, FE, and FD. The SNPs-based QTLs for SI, FL, BW, FU, FE, and FD were previously reported in the reference #6 (Ma et al. (2018) Nat. Genet. 50: 803-13). Some of these QTLs were functionally validated in the reference by VIGS, differential expression analysis, and/or overexpression. Here, the authors choose a different analytical method (CNV-based QTLs) to analyze the same phenotypic data as reported in reference #6 and identified different QTLs. The question is which loci are the major QTLs for the traits. The authors did not analyze the biological functions of any of their new QTLs. That is not enough, they need to validate at least one novel QTLs by transgenic cotton experiments (Overexpression, RNAi or genome editing system).

2. For figure 6, the authors listed previously identified genes in figure 6b and newly identified genes in figure 6c. I think the authors should select at least a new identified gene to validate its biological functions and explain the biological importance when the gene was lost or retained during cotton improvement.

3. The authors highlighted the gene Ghir_A08G006710 in figure 6d and e, with no further functional studies. It is necessary to dig out the biological function of the gene by CRISPR-directed genome editing system in cotton. They failed also to work on PAV variations among different cotton population and did not tell the readers how PAV impact gene functions to cause the phenotype.

Other concerns:

1. The title "1,913 accessions-based cotton pan-genome retrieve the lost sequences and genes during domestication and selection". In fact, they only sequenced 89 cotton accessions in the manuscript, most of the cotton accessions (1,824) comes from various cited references. So the title tends to mislead the readers and exaggerate their contributions. I suggest that the title changes to "Cotton pan-genome retrieve the lost sequences and genes during domestication and selection".

2. In line 26, "…of 1-70 (average 9.6) and 27 wild Gossypium species ((AD)3-(AD)7) (Table S1)." However, there are only 25 cotton accessions from ((AD)3-(AD)7) in their manuscript (6 Gossypium darwinii; 5 Gossypium ekmanianum; 5 Gossypium mustelinum; 9 Gossypium tomentosum). Gossypium arboreum is A2-genome and Gossypium davidsonii is D3-genome, not the AD-genome!

3. It's also very strange that according to Table 1, the SVs (duplication, inversion, translocation) for GhImpCHN were missing, but I can still see these values in Figure 1e!

4. According to Table 1 and Table S7, there are only 504 TRAs, however, the range of TRA reported in Figure 1e is 0-20,000!

5. For Table S1, it is very roughly and casually prepared. For the sample 'PG21' in Table S1, it was collected from the Gossypium stephensii species (AD7-genome), and the authors classified it as improved Gossypium hirsutum cultivars (Gh. improved). It is an obvious mistake!

6. For 'PG17' and 'PG18' in Table S1, both were collected from the Gossypium tomentosum species (AD3-genome). However, the authors named the sample 'PG17' as AD3-genome and 'PG18' as AD4-genome.

7. Line 34 in Supplementary Notes "…were retained in 1,913 and 1,625 allotetraploid cotton, respectively (Tables S2 and S4)." According to the description and Table S1, there are a total of 1,685 G. hirsutum accessions (1,424 Gh.improved; 256 Gh.landrace; 5 wild Yucatanense)! Even though the authors removed 54 duplicated cotton accessions from the 1,685 G. hirsutum accessions, there are also a total of 1,631 G. hirsutum accessions, not the described 1,625!

8. Line 95 in the main text "…19,246,497 SNPs and 4,815,125 InDels with a minor allele frequency (MAF) > 0.01." The authors described the criteria for MAF > 0.01, but in Table S2-4 the criteria is MAF ≥ 0.01. In Table S7, the filter criteria is MAF < 0.01 in population. For Table S8 and line 111 in the main text, the filter criteria is MAF > 0.05. Please be consistent!

9. In lines 107-108 in the main text "We used 742 cotton accessions with a high sequencing depth (> 10x) against the G. hirsutum TM-1 reference genome." Obviously in Table S1, there are only 718 cotton accessions with >10x, not 742!

10. The authors used the cotton accessions with >10x sequencing depth to perform structural variants (SVs). In my opinion, the sequence depth tends to cause false positive and lost useful information, it's better to select cotton accessions with at least 20x sequencing depth or use other experimental methods to validate the SVs.

11. According to Table S8, there are a total of 742 G. hirsutum cotton accessions, but in fact there are a total of 792 G. hirsutum cotton accessions, which include 258 Ghlandrace, 267 GhImpUSO, and 267 GhImpCHN!

12. In lines 86-87 in the main text "…54 duplicates were removed to give a total of 1,913 cotton accessions for a genomic variation analysis", yet they did not remove these duplicated cotton accessions in Table S1, there are still a total of 1,967 cotton accessions retained in that table, with the title "Supplementary Table 1. Summary of genomic sequencing data of 1,913 cotton accessions in this study."

13. In lines 89-91, the authors stated that there are 1,369 Gh.improved (438 GhImpUSO + 931 GhImpCHN) accessions. In fact, there are a total of 1,424 Gh.improved in their Table S1. How to explain the contradiction?

14. In lines 91-92 in the main text, "…from China (GhImpCHN), and 261 G. barbadense accessions (Additional file 1: Table S1)." The authors claimed 261 G. barbadense accessions, but reports only 254 in Table S1.

15. Lines 105-106 "…ImpCHN, and 1.01x 10-3 in G. barbadense (Additional file 2: Figure S3), similar to 106 recent studies in cotton [3-6] (Fig. 1d)." According to Fig. 1d, the Fst between Ghlandrace and GbImpI is just 0.191, which is similar to the Fst of Ghlandrace and GhImpUSO (0.139), but far lower than the Fst of GbImpI and GhImpUSO (0.308), the Fst of GbImpII and GhImpUSO (0.424). G. barbadense and G. hirsutum have long been separated into two different cotton species, so how could it be possible that the Fst of G. barbadense and G. hirsutum is as low as 0.191? These authors suggest that their results are similar to recent reports in reference #3-6. However, in reference #4, the Fst between G. barbadense and G. hirsutum reached up to 0.63 or 0.65.

16. In Table 1 and Table S7, the author claimed "The number of genotypes in each group are in parentheses." According to the description in Table 1, the 1,913 cotton accessions were used for SVs analysis, not 742 cotton accessions described in line 107-108 of the main text.

17. Based on their Table S1, the outgroup species contained Gossypium arboreum (A2-genome), Gossypium davidsonii (D3d-genome), Gossypium tomentosum (AD3), Gossypium mustelinum (AD4), Gossypium darwinii (AD5), Gossypium ekmanianum (AD6). For figures 1a, b, c and Figure S1, S2, only the (AD3)-(AD7) species were used as the outgroup, not Gossypium arboreum (A2-genome) and Gossypium davidsonii(D3d-genome). There are almost no descriptions and analysis for Gossypium arboreum and Gossypium davidsonii in the manuscript!

18. For the note in Table S1 "The AD1 and AD2 allotetraploid cotton genome predicted size is ~2300 megabases (Mb). Depth (x) was calculated as the total bases of clean reads divided by 2,300 Mb". The genome sizes for Gossypium arboreum A2-genome and Gossypium davidsonii were ~1700 Mb and ~800 Mb, respectively. So when the authors calculated the depth for these species, it is an obvious mistake to divide the total bases of clean reads from Gossypium arboreum A2-genome and Gossypium davidsonii by 2,300 Mb!

19. Lines 125-203, most of the identified QTLs and domestication sweep regions were analyzed in cited references, so the authors should focus on the newly identified useful loci and highlight the novel findings in this manuscript. I suggest the authors focus on the biological functions of their novel identified DSRs and QTLs by designing related experiments.

20. Lines 135-138: "The domestication selected genes were involved in stress response, cell wall regulation and circadian rhythm process for fiber elongation and environmental adaptation (Additional file 2: Figure S7)." Sorry I see no enriched process during fiber elongation, according to Figure S7.

21. Line 163: "…conducted a genome-wide association study (GWAS) meta-analysis of 890 G. hirsutum accessions from three independent experimental cases with multiple environments." The authors claimed that 890 cotton accessions were used for GWAS analysis, but in Table S1, "The 957 non-redundant cotton accessions used for GWAS analysis." Which is true?

22. Line 207: "The sequencing data of 1,581 G. hirsutum…". Here the authors selected 1,581 cotton accessions, but I did not know which cotton accessions were used for the analysis? What is the criteria for their selection?

23. The authors aligned all sequencing reads against TM-1 genome. Since there were huge genomic differences among cotton species, it's better to describe the mapping rate and informative reads in STables.

24. The phenotypic data for GWAS should be deposited in public database, such as cottongen or other website.

25. For the legend of Figure 2a, there are two labels with the same color, but with different scales for Dom_SVs.

26. For the figure 2b-i, the readers do not know the details of CNV in these loci because the authors did not further zoom into these sites to display the sequences or copy variations of CNVs. Also, they need to provide corresponding analysis in a figure or a supplementary figure.

27. The y-axis scales for figure 3a and c are not correct. The same scales represent different values!

28. For figure 6, the authors should zoom into the variation regions and display the details of PAV and sequence variations in cotton accessions for the listed genes in figure 6b, c and d. Also, they need to provide corresponding analysis in a figure or a supplementary figure.

29. The authors highlighted the a pleiotropic QTL for yield (LP, FWPB) and fiber quality-related traits (FM, FS, MAT) in figure 6d. However, the cotton population displayed significant differences only in LP and FWPB, not in FM, FS. So no enough evidences to support their idea that "This gene may be the causal PAV for fiber and yield pleiotropic QTL".

30. The unit for 'FS (g/text)' in figure 6e is wrong.

**Reviewer 3**

**Are you able to assess all statistics in the manuscript, including the appropriateness of statistical tests used?**

No, I do not feel adequately qualified to assess the statistics.

**Comments to author:**

Li et al re-sequenced 89 cultivated tetraploid cotton accessions of Gossypium hirsutum and downloaded the re-sequencing data of 1,679 G. hirsutum, 261 G. barbadense, and 27 wild tetraploid cotton accessions from National Center for Biotechnology Information (NCBI) database. Upon mining large amounts of genomic variation (SNP, InDel, and SV), they identified 456 Mb and 357 Mb of the sequence for domestication and selection signals including loci associated with agronomic traits. The authors conducted the genome-wide association study (GWAS) meta-analysis of 890 G. hirsutum accessions from three independent experiments with multiple environments. Their comparison of 125 major QTLs with 4,751 candidate genes for 17 agronomic traits indicated that 78 were similar with previous studies and the other 47 were likely new QTLs. They also conducted a pangenome analysis to discover 32,569 G. hirsutum and 8,851 G. barbadense genes that were not found in the previously sequenced reference genome of the two cotton genetic standards. These new genes included 124 presence/absence variation (PAV) for fiber quality and yield.

This study is one of the first pangenome studies into the genome variation of Gossypium genus. The GWAS and other analyses also represents a significant advance over previously published cotton studies. The materials and methods used are appropriate to the objective of this study. The conclusions are supported by the large amounts of genomic, genetic, and phenotypic data presented in the manuscript. The information generated from the study is of broad interest to plant researchers not only in cotton but also in other polyploid crops. While strongly recommending this well-prepared manuscript to the journal of Genome Biology, I only have one specific comment for the authors to consider:

1. The total number of genes in tetraploid cotton genomes is likely somewhere between 70,000 and 80,000. While new genes may be discovered between the species and accessions, it is possible to overestimate the gene numbers from different prediction programs and parameters among different studies. I would encourage the authors to evaluate these numbers to reveal how many of their 102,768 genes belong to actual duplicates and/or alleles of the same genes, not the genes otherwise lost in the reference genomes. Such evaluations should be reflected in the Results and Discussion sections.

**Authors Response**

**Point-by-point responses to the reviewers’ comments:**

Reviewer #1:

In their paper, Li et al. assemble a cotton pangenome using 1,913 accessions for two of the allopolyploid cottons Gossypium hirsutum and G. barbadense using the G. hirsutum reference genome. The authors map deletions associated with domestication, call SNPs and CNVs and run genome-wide association studies with those around traits of agronomic interest,

The outline and figure structure mostly follows the tomato pangenome (Gao et al., 2019, The tomato pan-genome uncovers new genes and a rare allele regulating fruit flavor), I can't find any major issues there, it's just not that novel in terms of findings. It should be interesting to people working in the cotton space, though.

*Response: Thanks very much for all your comments. We used an integrative genomics strategy to construct a multi-dimensional variome that is the largest variation dataset up to now in cotton and will be of interest to cotton researchers. Through haplotype analysis, gene expression, and gene functional annotation for a fiber elongation related novel QTL (mqFE253), a candidate gene (GhIDD7) was validated by CRISPR/Cas9 experiment. For other comments, we have thoroughly revised the original manuscript based on your suggestions below.*

Some minor things:

- I think the paper's introduction would explain the pangenome concept better to readers outside of the field if one or two of the [18-31] citations (line 69) would be summarised for the reader

*Response: Thanks very much for your suggestion. The concept of pan-genome has been explained according to pan-genomics studies in tomato (Gao et al., 2019, 51:1044-1051) and soybean (Liu et al., 2020, 182:162-176). Please see the details on lines 69-78 in the revised manuscript.*

- line 108: 'found identify 32,099 deletions' should probably be 'identified' or 'found' alone

*Response: Thanks very much for pointing out this mistake. We have deleted the word ‘found’, and changed ‘identify’ to ‘identified’.*

- line 133, 'including 274 homologous gene pairs' - should that be 'homeologous gene pairs' as these seem to be between the two subgenomes?

*Response: Thanks very much for your suggestion. This description represented “homeologous gene pairs”. We have also provided details for the identification of homologous gene pairs in the Method section. The reciprocal best BLAST hits were used to identify homeologous gene pairs between At- and Dt-subgenome. Syntenic blocks were detected using MCScanX (see lines 560-562) in the revised manuscript.*

Same in line 140. I may misunderstand this point as the methods do not state how the gene pairs were calculated, that needs to be added. I'm guessing MCScanX?

*Response: Thanks very much for your suggestion. The method for identification of homologous gene pairs has been added to the Method section in the revised manuscript.*

- line 136, 'circadian rhythm process for fiber elongation and environmental adaptation' - this sounds like the circadian rhythm is involved in fiber elongation, is that true? Similarly, I don't see anything directly about environmental adaptation in Suppl. Figure 7. Perhaps that should be rewritten

*Response: Thanks very much for your suggestion. As showed in Figure S7, seven genes in the D-subgenome are involved in the rhythmic response process, such as the well-known gene phytochrome B (PHYB). We reorganized the description in Figure S7 to include a more significant jasmonic acid and ethylene pathway (GO:0009861). We have deleted ‘for fiber elongation and environmental adaptation’ (see lines 150-152).*

- line 206, the citation for the 'reference-guided approach' points to the sunflower genome, but it should point to reference 21, Golicz et al., B. oleracea pangenome since that paper pioneered this approach in plants

*Response: Thanks very much for pointing out this mistake. We have changed this reference citation in the revised manuscript.*

- line 231, it says that 18.9 Mb of unmapped contigs were aligned, it would be helpful to have the percentage of the total size of all unmapped contigs here

*Response: Thanks very much for your suggestion. To verify the reliability of assembled non-reference sequences based on short reads, the long reads-based assemblies from 10 representative accessions were aligned to the ‘TM-1’ reference genome and G. hirsutum non-reference sequences. A total of 641 Mb contigs from 10 accessions that were not mapped on the TM-1 reference genome were aligned to the 1041 Mb non-reference sequences, of which 18.9 Mb (~3%) were aligned. The detailed contig mapping ratio has been shown in the revised manuscript (see lines 268-270).*

- line 340, it says 'For genes favorable to cotton breeding,' - this sounds like these genes are helpful in the process of breeding itself, while in reality, they were selected for during domestication, either during random bottleneck events or because they confer some trait of interest to breeders. The word should be changed

*Response: Thanks very much for your suggestion. We have rewritten this sentence and also provided the differential gene alleles for selection genes that might contribute to agronomic traits.*

- line 556, 'The best contig alignments against higher plants were considered as contaminant.' I'm guessing this should be NOT against higher plants, removing plants as contaminants would not be good.

*Response: Thanks very much for your suggestion. This was a description mistake. This sentence has been rewritten in the revised manuscript. The non-reference sequences that were not aligned to sequences of higher plants in NCBI nt database were considered as contaminant sequences. Our filtering standard of novel sequences was according to the rice pan-genome (Wang et al., 2018;557:43-49).*

- line 557, this sentence is a bit confusing to me: 'The Ghpan-genome and Gbpan-genome were generated by combining the 'TM-1' and '3-79' reference genome and respective non-redundant sequences.' However, the earlier text (line 92, 108) seem to imply that only TM-1 was used, while line 209 implies that the two genomes were used separately as reference to make *two* pangenomes. The authors should clarify which approach was used.

*Response: Thanks very much for your suggestion. In the pan-genome analysis, short reads from G. hirsutum and G. barbadense accessions were aligned to the ‘TM-1’ and ‘3-79’ reference genomes to obtain unaligned reads, respectively (see lines 635-639). In Figure 1, only ‘TM-1’ reference genome was used for multiple variation calling and divergence analysis of G. hirsutum and G. barbadense. For the G. barbadense accessions, the ‘3-79’ reference genome was also used for SNP calling and constructing a phylogenetic tree. Please see Table S5 and Figure R1 (shown below). We have provided these details in the revised manuscript.*

- line 590, I don't understand this sentence; 'The final transcripts were aligned using interproscan [] and added to the final gene models'. Interproscan is normally used to search protein sequences for domains while this sentence implies that interproscan was used to align the gene models with something, but with what?

*Response: Thanks very much for your suggestion. The protein sequences translated from transcripts were aligned to the Interproscan database. Transcripts with at least one evidence (Interpro, Pfam, GO, KEGG) supporting annotation were retained. The remaining transcripts were used to filter gene model. We have provided the description details in the Method section, please see lines 670-675.*

Reviewer #2: ===

Recent cotton genome advances greatly drove cotton population research and it is the best time to perform structural variations among populations and pan-genome analysis. In this manuscript, Li et al. collected 1,913 cotton accessions to do cotton genomic variome and identified lost sequences and genes during population development. Obviously, the work will be a great genetic resource for cotton community. I applaud for their idea and the great effort the researchers put in to generate this resource.

This paper is globally interesting. However, I do think the weakest point of this manuscript is the lack of functional analysis of their novel QTLs/PAVs. Although they retrieved the lost sequences and genes during domestication and selection, they have no direct evidences to support that these sequences and genes were biologically important. All of their analysis in the manuscript rely totally on the association between CNVs/SVs/PAV and previously reported QTLs, which seems very rudimentary and is difficult to determine whether their identified SVs, CNV, PAV, or QTLs were biologically important and whether their novel QTLs were the major functional sites, so I do think the authors need to confirm the biological functions of at least one or two novel identified QTLs by transgenic cotton experiments (Overexpression, RNAi or genome editing system) (see my major concerns #1, 2, and 3). In summary, the manuscript has to go through a thorough revision with several new experiments before it can be considered for publication in Genome Biology!

*Response: Thanks very much for all your suggestions that help us to improve this manuscript. We have revised the manuscript thoroughly based on your suggestions.*

*In the revised manuscript, we mainly modified several aspects shown below:*

*1) We performed more analyses of a novel QTL (mqFE253) associated with fiber elongation (FE). A candidate gene (Ghir_D05G013680, GhIDD7) of mqFE253 was validated by CRISPR/Cas9 gene editing system, which encodes an indeterminate-domain 7 transcription factor. This gene was differentially expressed in different fiber development stages and was highly expressed at 30 DPA fibers. After knocking out this gene, the mature fiber in mutant plants was shorter than that in wild-type plants. We inferred that GhIDD7 was a previously uncharacterized transcription factor gene related to fiber development. These data were shown in Figure S10 and Figure S11.*

*2) We validated some CNVs and PAVs in non-reference genome sequences using reads mapping and PCR experiment. These data were shown in Figure S12 and Figure S19.*

*3) We performed a detailed analysis of domesticated genes in Figure 6, including SNP allele information, expression patterns between landraces and cultivars, and their tissue expression patterns. For Figure 6c, the PAV alignment was shown between representative presence and absence accessions. For Figure 6d, the PAV mapping was also visualized in some randomly selected GhImpCHN cultivars. Further analysis showed that Ghir_A08G006710 existed in landrace and GhImpUSO populations. However, a considerable number of cultivars in the GhImpCHN population did not have this gene, which suggested that this represented a recent gene loss event during cotton improvement. Further, we found that absence or presence of this gene coupled with significant changes in the expression of adjacent genes. These data were shown in Figure S26 and Figure S28.*

Major concerns:

1. For Figure 2b-i, the authors identified novel QTLs based-on CNVs and listed several examples for SI, FL, BW, FU, FE, and FD. The SNPs-based QTLs for SI, FL, BW, FU, FE, and FD were previously reported in the reference #6 (Ma et al. (2018) Nat. Genet. 50: 803-13). Some of these QTLs were functionally validated in the reference by VIGS, differential expression analysis, and/or overexpression. Here, the authors choose a different analytical method (CNV-based QTLs) to analyze the same phenotypic data as reported in reference #6 and identified different QTLs. The question is which loci are the major QTLs for the traits. The authors did not analyze the biological functions of any of their new QTLs. That is not enough, they need to validate at least one novel QTLs by transgenic cotton experiments (Overexpression, RNAi or genome editing system).

*Response: Thanks very much for your suggestions. In this study, we have conducted QTL analysis based on data from three independent experimental case studies. Ten novel QTLs for fiber elongation were identified in 890 panel accessions (Figure S10). We validated a novel QTL (mqFE253) located on the D05 chromosome. Some candidate genes were mined by integrating haplotype analysis, gene expression, and functional annotation (Figure S10). One candidate gene (Ghir_D05G013680, GhIDD7), an indeterminate-domain 7 transcription factor, was differentially expressed during four stages of fiber development. The two main haplotypes showed a significant negative correlation between fiber elongation and length (Figure S11b). After knockout of GhIDD7 in G. hirsutum acc. Jin668 cultivar, the mature fiber length was significantly shorter (25.8±0.3) compared with wild type (27.1±0.1) plants (Figure S11d, e). These results inferred that GhIDD7 was a previously uncharacterized gene contributed to fiber quality-related trait.*

2. For figure 6, the authors listed previously identified genes in figure 6b and newly identified genes in figure 6c. I think the authors should select at least a new identified gene to validate its biological functions and explain the biological importance when the gene was lost or retained during cotton improvement.

*Response: Thanks very much for your suggestion. We have performed more analyses for genes in Figure 6, such as expression levels in a few semi-wild and cultivated accessions (Figure 6d and Table S30) and haplotype analysis. We are trying to construct some bi-parent populations to explore the biological function roles of these PAVs, and also generate more CRISPR-Cas9 mutants to validate their function. However, it should take at least a couple of years to fulfill these experiments.*

3. The authors highlighted the gene Ghir_A08G006710 in figure 6d and e, with no further functional studies. It is necessary to dig out the biological function of the gene by CRISPR-directed genome editing system in cotton. They failed also to work on PAV variations among different cotton population and did not tell the readers how PAV impact gene functions to cause the phenotype.

*Response: Thanks very much for your suggestions. This PAV has been displayed (Figure R2). The presence frequency analysis showed that Ghir_A08G006710 was present in nearly all landrace (182) and GhImpUSO (205) accessions, but this gene was absent in many GhImpCHN cultivars with a low presence frequency (10.5%, 60 of 592 GhImpCHN accessions). This result indicated that this gene might represent an unfavorable (loss) gene in the recent breeding process (Fig. 6g). Because this gene is present in cultivar Jin668 that is used for transgenic transformation experiment in our lab, we are trying to knock out this gene to confirm its biological function, which will take at least a couple of years. Expression analysis in multiple tissues showed that this gene exhibited a dominant expression in fiber development (Figure S29a). Further, based on our recently published population RNA-Seq data of 15 DPA fiber from 251 upland cotton accessions (Li et al., 2020; 226:1738-1752), we investigated the possible regulatory effect of the presence and absence haplotype of this gene on flanking genomic region (200 Kb). We found that this gene was absent in 18 accessions and was present in 233 accessions. We investigated the gene expression flanking 200 Kb region which contained 17 genes and found that 4 genes were differentially expressed in the absence and presence of haplotypes. Among them, one gene, Ghir_A08G006730 (AUX/IAA transcriptional regulator family protein), exhibited significantly lower expression in accessions with the absence haplotype than those with the presence haplotype (Figure S29b). These results indicated that the presence and absence of Ghir_A08G006710 might be related to the expression of adjacent genes. Therefore, we think that this gene represented a gene loss event during cotton breeding, which may be involved in the expression regulation of AUX/IAA related gene and thus contributes to the phenotype difference.*

Other concerns:

1. The title "1,913 accessions-based cotton pan-genome retrieve the lost sequences and genes during domestication and selection". In fact, they only sequenced 89 cotton accessions in the manuscript, most of the cotton accessions (1,824) comes from various cited references. So the title tends to mislead the readers and exaggerate their contributions. I suggest that the title changes to "Cotton pan-genome retrieve the lost sequences and genes during domestication and selection".

*Response: Thanks very much for your suggestion. We have changed the title to “Cotton pan-genome retrieves the lost sequences and genes during domestication and selection”.*

2. In line 26, "…of 1-70 (average 9.6) and 27 wild Gossypium species ((AD)3-(AD)7) (Table S1)." However, there are only 25 cotton accessions from ((AD)3-(AD)7) in their manuscript (6 Gossypium darwinii; 5 Gossypium ekmanianum; 5 Gossypium mustelinum; 9 Gossypium tomentosum). Gossypium arboreum is A2-genome and Gossypium davidsonii is D3-genome, not the AD-genome!

*Response: Thanks very much for pointing out this error. The information of Gossypium arboreum (BYU80001, BYU80002) and Gossypium davidsonii (D3D) has been added to Method section in the revised manuscript. In 1,913 population, the outgroup (29 species) included 8 Gossypium tomentosum (AD3), 6 Gossypium darwinii (AD5), 6 Gossypium mustelinum (AD4), 5 Gossypium ekmanianum (AD6), 1 Gossypium stephensii (AD7), 2 Gossypium arboretum (A2), and 1 Gossypium davidsonii (D3) cottons.*

3. It's also very strange that according to Table 1, the SVs (duplication, inversion, translocation) for GhImpCHN were missing, but I can still see these values in Figure 1e!

*Response: Thanks very much for your concern. Figure 1e showed the distribution of SV numbers in different accessions compared with ‘TM-1’ reference genome, but Table 1 showed the number of combined SVs filtered by MAF < 0.01 instead of the SV number in each accession of the GhImpCHN population. DUP, INV, and TRA have a low frequency distribution in the population. We did not merge them in GhImpCHN population but identified the number in each accession compared with TM-1 reference genome. We have changed detailed information in the revised manuscript.*

4. According to Table 1 and Table S7, there are only 504 TRAs, however, the range of TRA reported in Figure 1e is 0-20,000!

*Response: Thanks very much for your concern. We have checked this data. It is known that TRA variation is in a relatively low frequency in specific population, which is the number of TRA compared to the reference genome. Compared with the TM-1 reference genome, the average of TRA number was 732, 894, 1870 in GhImpCHN, GhImpUSO, Ghlandrace population, while the GbImpI, GbImpII, outgroup was 3902, 4434, 6167, respectively. Due to the large number of TRA in individual of the Outgroup, the coordinate y-axis became larger, and these TRAs with low frequency were filtered out during merging. Therefore, we removed cotton accessions with the number of TRA more than 10,000 to display in the Figure 1e. We have also provided detailed description information in Table 1.*

5. For Table S1, it is very roughly and casually prepared. For the sample 'PG21' in Table S1, it was collected from the Gossypium stephensii species (AD7-genome), and the authors classified it as improved Gossypium hirsutum cultivars (Gh. improved). It is an obvious mistake!

*Response: Thanks very much for your suggestion. We have carefully checked all the information in the revised manuscript. The Gossypium stephensii species is represented with ‘AD7’ now.*

6. For 'PG17' and 'PG18' in Table S1, both were collected from the Gossypium tomentosum species (AD3-genome). However, the authors named the sample 'PG17' as AD3-genome and 'PG18' as AD4-genome.

*Response: Thanks very much for your suggestion. The information of ‘PG17’ and ‘PG18’ species was represented with Gossypium tomentosum (AD3) and Gossypium mustelinum (AD4), respectively. These ambiguous information have been corrected in the revised manuscript.*

7. Line 34 in Supplementary Notes "…were retained in 1,913 and 1,625 allotetraploid cotton, respectively (Tables S2 and S4)." According to the description and Table S1, there are a total of 1,685 G. hirsutum accessions (1,424 Gh.improved; 256 Gh.landrace; 5 wild Yucatanense)! Even though the authors removed 54 duplicated cotton accessions from the 1,685 G. hirsutum accessions, there are also a total of 1,631 G. hirsutum accessions, not the described 1,625!

*Response: Thanks very much for pointing out this error. We discarded 54 sequencing data from duplicated accessions, and the final accession number is 1,913 for SNP variation analysis, including 1,623 G. hirsutum accessions (256 landrace, 438 ImpUSO, 929 ImpCHN), 261 G. barbadense accessions and 29 other Gossypium accessions/species. We have corrected the ambiguous information. In our analysis, 6 G. hirsutum ycatanense and 250 G. hirsutum landrace accessions were categorized to the landrace group (256). Please see the Table S1 in the revised manuscript.*

8. Line 95 in the main text "…19,246,497 SNPs and 4,815,125 InDels with a minor allele frequency (MAF) > 0.01." The authors described the criteria for MAF> 0.01, but in Table S2-4 the criteria is MAF ≥ 0.01. In Table S7, the filter criteria is MAF< 0.01 in population. For Table S8 and line 111 in the main text, the filter criteria is MAF> 0.05. Please be consistent!

*Response: Thanks very much for pointing out this inconsistency. We have carefully corrected it at all points in Table S2-S4 and Table S7. We have checked data in Table S8, with a MAF of 0.01 as the selection criteria.*

9. In lines 107-108 in the main text "We used 742 cotton accessions with a high sequencing depth (> 10x) against the G. hirsutum TM-1 reference genome." Obviously in Table S1, there are only 718 cotton accessions with >10x, not 742!

*Response: Thanks very much for pointing out this inconsistency. These data were rechecked, and additional data for SV analysis have been added in Table S1. The 742 accessions including 693 accessions (sequencing depth > 10×) of 1,913 population and 49 accessions in other SRA projects (PRJNA321738, PRJNA450479, PRJNA576032) were also used for SV analysis. This detail information was shown in Table S8 and Supplementary Note 1.*

10. The authors used the cotton accessions with >10x sequencing depth to perform structural variants (SVs). In my opinion, the sequence depth tends to cause false positive and lost useful information, it's better to select cotton accessions with at least 20x sequencing depth or use other experimental methods to validate the SVs.

*Response: Thanks very much for your suggestion. Of course, higher sequencing depth will allow the identification of more accurate SVs. In this study, we aimed at discovering the difference of SVs between landraces and improved cultivars. The selection of cotton accessions with >10× sequencing coverage allows the use of more accessions for analysis. In fact, according to CNVcaller instructions, resequencing data with a depth of more than 5 can still be used for CNV calling, as described in the citation reference (Wang et al., 2017;6:1-12). To control the false positive rate of SV identification in 742 accessions, we selected 10 diploid species (D3, A2, D6, B1, K1 genomes) with high sequencing depth as the first outgroup, 19 tetraploid Gossypium species (AD3-AD7) as the second outgroup. According to the analysis of CNV population structure, the first outgroup and the second outgroup can be clearly separated from G. hirsutum accessions at different K values (K = 3, K = 5 and K = 8) (Figure S4e).*

11. According to Table S8, there are a total of 742 G. hirsutum cotton accessions, but in fact there are a total of 792 G. hirsutum cotton accessions, which include 258 Ghlandrace, 267 GhImpUSO, and 267 GhImpCHN!

*Response: Thanks very much for your concern. In this study, only 742 cottons were used for SV analysis. These detailed information of cotton accessions has been provided in Table S1. The 521 G. hirsutum cottons included 251 landrace accessions, 240 accessions from GhImpUSO, and 30 accessions from GhImpCHN. We have revised the accessions information in Table S1 and Table S8.*

12. In lines 86-87 in the main text "…54 duplicates were removed to give a total of 1,913 cotton accessions for a genomic variation analysis", yet they did not remove these duplicated cotton accessions in Table S1, there are still a total of 1,967 cotton accessions retained in that table, with the title "Supplementary Table 1. Summary of genomic sequencing data of 1,913 cotton accessions in this study."

*Response: Thanks very much for your suggestion. We have deleted the 54 duplicated accessions in Table S1. All the accessions have been checked carefully in the revised manuscript.*

13. In lines 89-91, the authors stated that there are 1,369 Gh.improved (438 GhImpUSO + 931 GhImpCHN) accessions. In fact, there are a total of 1,424 Gh.improved in their Table S1. How to explain the contradiction?

*Response: Thanks very much for your question. This information has been revised and checked in Table S1. In the original manuscript, the GhImpCHN population with accessions from Nanjing Agricultural University (NJAU) and Hebei Agricultural University (HBAU) project had 54 duplicated accessions. And one accession (Gossypium davidsonii) was mistakenly identified as Gossypium hirsutum. The calculation of duplicated accessions: 1424-1369-1=54.*

14. In lines 91-92 in the main text, "…from China (GhImpCHN), and 261 G. barbadense accessions (Additional file 1: Table S1)." The authors claimed 261 G. barbadense accessions, but reports only 254 in Table S1.

*Response: Thanks very much for your suggestion. The details of 261 G. barbadense have been shown in Table S1 of revised manuscript.*

15. Lines 105-106 "…ImpCHN, and 1.01x 10-3 in G. barbadense (Additional file 2: Figure S3), similar to 106 recent studies in cotton [3-6] (Fig. 1d)." According to Fig. 1d, the Fst between Ghlandrace and GbImpI is just 0.191, which is similar to the Fst of Ghlandrace and GhImpUSO (0.139), but far lower than the Fst of GbImpI and GhImpUSO (0.308), the Fst of GbImpII and GhImpUSO (0.424). G. barbadense and G. hirsutum have long been separated into two different cotton species, so how could it be possible that the Fst of G. barbadense and G. hirsutum is as low as 0.191? These authors suggest that their results are similar to recent reports in reference #3-6. However, in reference #4, the Fst between G. barbadense and G. hirsutum reached up to 0.63 or 0.65.

*Response: Thanks very much for your suggestion. According to minor allele frequency (MAF) threshold of 0.01 in 1,913 population, the fixation index (Fst) was calculated for multiple comparisons of populations. We obtained two Fst values (‘weighted Fst’ and ‘mean Fst’) from VCFtools. The formula of weighted Fst is sum(numerators) / sum(denominator), but mean Fst is average per site. So, we used weighted Fst in a window to evaluate the divergence between two populations. The weighted Fst between Ghlandrace and GbImpI is 0.58, which is consistent with reference #4 results. Other comparison groups were also re-evaluated: the Fst = 0.294 between Ghlandrace and GhImpUSO, the Fst = 0.784 between GbImpI and GhImpUSO, the Fst = 0.796 between GbImpII and GhImpUSO. These results are consistent with some references (Fang et al., Genome Biol. 2017;18:33; Nie et al., Plant J 2020;103(2):677-689). The Fst values were evaluated for both At and Dt subgenomes. Please see the Figure 1d and Additional 2: Figure S3.*

16. In Table 1 and Table S7, the author claimed "The number of genotypes in each group are in parentheses." According to the description in Table 1, the 1,913 cotton accessions were used for SVs analysis, not 742 cotton accessions described in line 107-108 of the main text.

*Response: Thanks very much for your question. We have revised the description for the data in Table S1. We have also explained this under the Table notes in Table 1.*

17. Based on their Table S1, the outgroup species contained Gossypium arboreum (A2-genome), Gossypium davidsonii (D3d-genome), Gossypium tomentosum (AD3), Gossypium mustelinum (AD4), Gossypium darwinii (AD5), Gossypium ekmanianum (AD6). For figures 1a, b, c and Figure S1, S2, only the (AD3)-(AD7) species were used as the outgroup, not Gossypium arboreum (A2-genome) and Gossypium davidsonii (D3d-genome). There are almost no descriptions and analysis for Gossypium arboreum and Gossypium davidsonii in the manuscript!

*Response: Thanks very much for your question. In 1,913 population, a total of 29 Gossypium species were used as outgroup, including genomes of AD3-AD7, A2 and D3. The descriptions in Figure 1a, 1b and Figure S1, Figure S2 have been revised.*

18. For the note in Table S1 "The AD1 and AD2 allotetraploid cotton genome predicted size is ~2300 megabases (Mb). Depth (x) was calculated as the total bases of clean reads divided by 2,300 Mb". The genome sizes for Gossypium arboreum A2-genome and Gossypium davidsonii were ~1700 Mb and ~800 Mb, respectively. So when the authors calculated the depth for these species, it is an obvious mistake to divide the total bases of clean reads from Gossypium arboretum A2-genome and Gossypium davidsonii by 2,300 Mb!

*Response: Thanks very much for your suggestion. We have re-evaluated the sequenced depth for all G. hirsutum accessions based on 2300 megabases (Mb) of genome size (Wang et al., 2019; Hu et al., 2019). The genome sizes of other species are from a previous study (Wendel JF and Grover CE, 2015). Sequencing depth for two Gossypium arboretum species and one Gossypium davidsonii species was calculated based on their genome sizes (1700 Mb and 800 Mb). All the sequencing depth information has been shown in Table S1. The sequencing depth of Gossypium anomalum (B1) and Gossypium exiguum (K1) accessions were also calculated according to genome sizes 1350 Mb and 2570 Mb.*

19. Lines 125-203, most of the identified QTLs and domestication sweep regions were analyzed in cited references, so the authors should focus on the newly identified useful loci and highlight the novel findings in this manuscript. I suggest the authors focus on the biological functions of their novel identified DSRs and QTLs by designing related experiments.

*Response: Thanks very much for your suggestion. We have highlighted the novel QTLs in this study and validated one fiber elongation related novel QTL using CRISPR/Cas9 experiment. Besides, the expression of domestication related genes was surveyed using public RNA-Seq data at 10 DPA and 20 DPA fibers between wild/landraces and improved cultivars (Yoo and Wendel, 2014). For example, the Ghir_A03G008350 (Squamosa promoter-binding protein-like, SPL) and Ghir_D01G018080 (Pectin lyase-like superfamily protein) in novel DSR exhibited higher expression levels at 10 DPA and 20 DPA fibers in wild than in cultivars (Figure S6a, c). In Figure 6b, we found that Ghir_A12G025340 (GRF) and Ghir_D13G021640 (PRF3) genes were highly expressed in cultivars. The Ghir_D03G008950 (CIP) and Ghir_D10G015750 (KCS2) were highly expressed in landraces (Figure S6d). Some fiber-related domesticated genes were differentially expressed between wild/landraces and improved cultivars.*

20. Lines 135-138: "The domestication selected genes were involved in stress response, cell wall regulation and circadian rhythm process for fiber elongation and environmental adaptation (Additional file 2: Figure S7)." Sorry I see no enriched process during fiber elongation, according to Figure S7.

*Response: Thanks very much for your suggestion. As can be seen from the Figure S7, genes in jasmonic acid and ethylene (GO:0009861) pathway were significantly enriched. Many previous studies have shown that the ethylene and other plant hormone (JA, IAA) pathway were involved in fiber elongation (Huang et al., Annu. Rev. Plant Biol. 2021. 72:2.1–2.26; Shi et al., 2007. 18:651–64). Since there were not so many direct functional evidence of these genes for fiber elongation and environmental adaptation, we deleted the description of “for fiber elongation and environmental adaptation”.*

21. Line 163: "…conducted a genome-wide association study (GWAS) meta-analysis of 890 G. hirsutum accessions from three independent experimental cases with multiple environments." The authors claimed that 890 cotton accessions were used for GWAS analysis, but in Table S1, "The 957 non-redundant cotton accessions used for GWAS analysis." Which is true?

*Response: Thanks very much for your question. Table S1 has been carefully checked. A total of 890 accessions were used for meta genome-wide association study. The phenotype and genotype data have been submitted to figshare website https://figshare.com/s/cb3c104782a1dcd90ab0/articles/13625771.*

22. Line 207: "The sequencing data of 1,581 G. hirsutum…". Here the authors selected 1,581 cotton accessions, but I did not know which cotton accessions were used for the analysis? What is the criteria for their selection?

*Response: Thanks very much for your question. The 1,581 G. hirsutum accessions were assembled into non-reference sequences, of which 1,020 accessions (sequencing coverage > 5) were used for PAV analysis. These accession information has been added to Table S1. The outgroup species (AD3-AD7 and diploid species) and unaligned paired reads were not used for pan-genomic construction.*

23. The authors aligned all sequencing reads against TM-1 genome. Since there were huge genomic differences among cotton species, it's better to describe the mapping rate and informative reads in STables.

*Response: Thanks very much for your suggestion. The informative mapping reads and mapping rates have been added to Table S1.*

24. The phenotypic data for GWAS should be deposited in public database, such as cottongen or other website.

*Response: Thanks very much for your suggestion. We have deposited all the phenotypic and genotype data of 890 accessions to public Figshare website https://figshare.com/s/cb3c104782a1dcd90ab0/articles/13625771.The BLUP breeding values of 13 agronomic traits in 419 panel accessions were obtained across 12 environments (Ma et al., 2018). The BLUP breeding values of 15 agronomic traits in 264 panel accessions were obtained across 12 environments (Huang et al., 2017). The BLUP breeding values of 15 agronomic traits in 207 panel accessions were obtained across 9 environments (Fang et al., 2017). The fiber length (FL), fiber elongation (FE), fiber strength (FS), fiber uniformity (FU) and fiber micronaire (FM) BLUP breeding values were combined from three case studies in non-redundant panel accessions.*

25. For the legend of Figure 2a, there are two labels with the same color, but with different scales for Dom_SVs.

*Response: Thanks very much for your suggestion. The different scales for Dom_SVs have been shown with different colors in the revised Figure 2a.*

26. For the figure 2b-i, the readers do not know the details of CNV in these loci because the authors did not further zoom into these sites to display the sequences or copy variations of CNVs. Also, they need to provide corresponding analysis in a figure or a supplementary figure.

*Response: Thanks very much for your suggestion. The lead CNV loci have been shown using representative cotton accessions for each genotype (Figure S12).*

27. The y-axis scales for figure 3a and c are not correct. The same scales represent different values!

*Response: Thanks very much for your suggestion. Because there are different numbers of genes at different frequencies, the exponent of dividing the axes into 10 is based on the R function math_format (10^.x) according to a previous published reference (Gao et al., Nat Genet. 2019; 51:1044-1051). Here is the truncated coordinate true y-axis (Figure R2), but this does not seem to be a better snapshot of low frequency genes. So, we would like to keep the original Figure 3a and Figure 3c.*

28. For figure 6, the authors should zoom into the variation regions and display the details of PAV and sequence variations in cotton accessions for the listed genes in figure 6b, c and d. Also, they need to provide corresponding analysis in a figure or a supplementary figure.

*Response: Thanks very much for your suggestion. The SNPs and PAVs variation regions have been shown with representative cultivars. In Figure 6d, PAV variations in the Ghir_A08G006710 and their flanking 2 Kb regions have been shown in 20 cotton accessions. Please see Figure S28.*

29. The authors highlighted the a pleiotropic QTL for yield (LP, FWPB) and fiber quality-related traits (FM, FS, MAT) in figure 6d. However, the cotton population displayed significant differences only in LP and FWPB, not in FM, FS. So no enough evidences to support their idea that "This gene may be the causal PAV for fiber and yield pleiotropic QTL".

*Response: Thanks very much for your suggestion. We have thoroughly analyzed the gene (Ghir_A08G006710) of figure 6d, including sequence variation, gene expression of flanking regions in accessions with presence and absence haplotypes. This gene may be a candidate gene for fiber yield trait.*

30. The unit for 'FS (g/text)' in figure 6e is wrong.

*Response: Thanks very much for your suggestion. The ‘g/text’ has been changed to ‘cN/tex’.*

Reviewer #3: Li et al re-sequenced 89 cultivated tetraploid cotton accessions of Gossypium hirsutum and downloaded the re-sequencing data of 1,679 G. hirsutum, 261 G. barbadense, and 27 wild tetraploid cotton accessions from National Center for Biotechnology Information (NCBI) database. Upon mining large amounts of genomic variation (SNP, InDel, and SV), they identified 456 Mb and 357 Mb of the sequence for domestication and selection signals including loci associated with agronomic traits. The authors conducted the genome-wide association study (GWAS) meta-analysis of 890 G. hirsutum accessions from three independent experiments with multiple environments. Their comparison of 125 major QTLs with 4,751 candidate genes for 17 agronomic traits indicated that 78 were similar with previous studies and the other 47 were likely new QTLs. They also conducted a pangenome analysis to discover 32,569 G. hirsutum and 8,851 G. barbadense genes that were not found in the previously sequenced reference genome of the two cotton genetic standards. These new genes included 124 presence/absence variation (PAV) for fiber quality and yield.

This study is one of the first pangenome studies into the genome variation of Gossypium genus. The GWAS and other analyses also represents a significant advance over previously published cotton studies. The materials and methods used are appropriate to the objective of this study. The conclusions are supported by the large amounts of genomic, genetic, and phenotypic data presented in the manuscript. The information generated from the study is of broad interest to plant researchers not only in cotton but also in other polyploid crops. While strongly recommending this well-prepared manuscript to the journal of Genome Biology, I only have one specific comment for the authors to consider:

1. The total number of genes in tetraploid cotton genomes is likely somewhere between 70,000 and 80,000. While new genes may be discovered between the species and accessions, it is possible to overestimate the gene numbers from different prediction programs and parameters among different studies. I would encourage the authors to evaluate these numbers to reveal how many of their 102,768 genes belong to actual duplicates and/or alleles of the same genes, not the genes otherwise lost in the reference genomes. Such evaluations should be reflected in the Results and Discussion sections.

*Response: Thanks very much for your comments. We appreciate this suggestion and believe that improved gene prediction will contribute to accuracy in pan-genome analysis. For gene prediction, a total of 1,041 Mb and 309 Mb non-reference sequences were used for gene prediction in G. hirsutum and G. barbadense. After two rounds of MAKER2 coupling with AUGUSTUS and SNAP, there were 90,376 and 22,969 transcripts in G. hirsutum and G. barbadense non-reference genomes, respectively. We have carried out several filtering steps for non-reference genes including protein annotation evidence, non-reference genes alignment references, gene location in contigs, all-by-all alignment to get reliable non-redundant non-reference gene sequences. Finally, 32,569 (65,679 transcripts) and 8,851 (12,076 transcripts) genes were obtained in G. hirsutum and G. barbadense for further pan genes analysis. Please see the Table R1. The final non-reference genes were aligned with other TM-1 reference genes (Hu et al., 2019; Chen et al., 2020) to ensure that all genes were non-redundant.*

*In Figure 3a, the frequency distribution of PAVs showed that there were large numbers of shell (3,803 genes with frequency 1%-97% in 1,020 population) and cloud (12,434 genes with frequency 0-1% in 1,020 population) genes in G. hirsutum pan-genome. Many accession-specific genes were found, especially in landrace accessions (Fig. 5d).*

*The current pan-genome has many contig sequences that were fragmented, leading to incomplete gene annotations. In the revised version, we have added some details of filtering steps for non-reference genes through all-by-all alignment to filter potential redundant non-reference sequences in the Method section. In the future, the annotation of non-reference genes may be combined with population transcriptome data, which can improve the accuracy for predicting coding genes and non-coding RNAs. This idea has been added to the discussion section in the revised manuscript. Again, thank you very much for your suggestion regarding of evaluating the number of pan genes in cotton.*

*References*

*1. Gao L, Gonda I, Sun H, Ma Q, Bao K, Tieman DM, Burzynski-Chang EA, Fish TL, Stromberg KA, Sacks GL, et al. The tomato pan-genome uncovers new genes and a rare allele regulating fruit flavor. Nat Genet. 2019;51:1044-1051.*

*2. Liu Y, Du H, Li P, Shen Y, Peng H, Liu S, Zhou G-A, Zhang H, Liu Z, Shi M, et al. Pan-Genome of Wild and Cultivated Soybeans. Cell. 2020;182:162-176.*

*3. Ma Z, He S, Wang X, Sun J, Zhang Y, Zhang G, Wu L, Li Z, Liu Z, Sun G, et al. Resequencing a core collection of upland cotton identifies genomic variation and loci influencing fiber quality and yield. Nat Genet. 2018;50:803-813.*

*4. Huang C, Nie X, Shen C, You C, Li W, Zhao W, Zhang X, Lin Z. Population structure and genetic basis of the agronomic traits of upland cotton in China revealed by a genome-wide association study using high-density SNPs.*

*5. Wang M, Tu L, Lin M, Lin Z, Wang P, Yang Q, Ye Z, Shen C, Li J, Zhang L, et al. Asymmetric subgenome selection and cis-regulatory divergence during cotton domestication. Nat Genet. 2017;49:579-587.*

*6. Fang L, Wang Q, Hu Y, Jia Y, Chen J, Liu B, Zhang Z, Guan X, Chen S, Zhou B, et al. Genomic analyses in cotton identify signatures of selection and loci associated with fiber quality and yield traits. Nat Genet. 2017;49:1089-1098.*

*7. Wang W, Mauleon R, Hu Z, Chebotarov D, Tai S, Wu Z, Li M, Zheng T, Fuentes RR, Zhang F, et al. Genomic variation in 3,010 diverse accessions of Asian cultivated rice. Nature. 2018;557:43-49.*

*8. Huang G, Huang JQ, Chen XY, Zhu YX, Recent Advances and Future Perspectives in Cotton Research. Annu. Rev. Plant Biol. 2021;72:2.1-2.26.*

*9. Shi YH, Zhu SW, Mao XZ, Feng JX, Qin YM, et al. Transcriptome profiling, molecular biological, and physiological studies reveal a major role for ethylene in cotton fiber cell elongation. Plant Cell. 2006;18:651–64.*

*10. Wendel JF and Grover CE. Taxonomy and Evolution of the Cotton Genus, Gossypium Cotton.*

*2nd. ed. Madison, WI, USA: Agronomy Monograph. 2010; 25-44.*

*11. Yoo NJ and Wendel JF. Comparative Evolutionary and Developmental Dynamics of the Cotton*

*(Gossypium hirsutum) Fiber Transcriptome. PLoS Genet. 2014;10:e1004073.*

*12. Wang X, Zheng Z, Cai Y, Chen T, Li C, Fu W, Jiang Y. CNVcaller: highly efficient and widely applicable software for detecting copy number variations in large populations. Gigascience. 2017;6:1-12.*

**Second round of review**

**Reviewer 1**

I am very satisfied with the replies from the authors, all good!

**Reviewer 2**

The manuscript has been improved greatly. In the revised manuscript, the authors failed to confirm the functions of their highlighted PAV for Ghir_A08G006710 gene by constructing transgenic cotton lines. Instead, they analyzed the expression of nearby genes and suggest that the PAV may regulate the expression of these adjacent genes (AUX/IAA related) and thus contributes to the reported phenotype. I understand the authors’ reluctances in carrying out full-scale transgenic cotton experiments, however, they need, at least, to analyze the levels of related plant hormones (not only IAA) during cotton fiber development in the selected cotton accessions, with or without the PAV.

Other concerns:

1. The authors listed 12 references in the “point-to-point response” file as the response to reviewers. They need to put these references in their manuscript accordingly.

2. For the newly added 5 references in the revised manuscript (ref. #85-89), 4 of these cited references were from the authors’ lab. Again, the ref. #88 and 89 have the same author name and title, and also were not peer-reviewed. DELETE ALL OF THEM!

3. For the newly added Figure S6d, the authors did not describe how they normalized the level of gene expression. I did not understand why the expression values of KCS2 for both wild and cultivar were lower than 0!

4. For figure 6g, the legend color for ‘Presence’ is not correct.

5. It’s not a good choice to delete the description of “for fiber elongation and environmental adaption”. I suggest the authors just rework the sentence and add related references.

6. For the newly added Figure S11a, the author should also check the DNA sequence in the edited region for the At-subgenome homologous gene, not only the target region in the Dt-subgenome.

7. For the newly added Figure S19, the two gel pictures were too dirty and ugly. Please redo it with enough repeats.

8. Line 486-488, according to the revised Table S1, there are 1,874 reported cotton accessions and 87 newly sequenced cotton accessions, not 1,872 and 89, respectively.

9. According to the point-to-point response, the authors did not analyze and combine the DUP, INV, TRA because the low frequency distribution in the population. It’s better to clarify this in the note in Table 1.

10. The author need to keep all barbadense TRA accessions in Figure 1e. I suggest them to use two panels (G. hirsutum population and G. barbadense population) with different scales to display the data.

11. No description for Figure S6b in the main text in this revised version. Delete this part of the figure or cite it in the text!

12. The authors deleted the FS and FM for the figure 6f in this revised version. They need to retain these parts and revising the description in the manuscript.

**Authors Response**

**Point-by-point responses to the reviewers’ comments:**

Reviewer #1: I am very satisfied with the replies from the authors, all good!

*Thanks very much for all your previous comments and suggestions!*

Reviewer #2: The manuscript has been improved greatly. In the revised manuscript, the authors failed to confirm the functions of their highlighted PAV for Ghir_A08G006710 gene by constructing transgenic cotton lines. Instead, they analyzed the expression of nearby genes and suggest that the PAV may regulate the expression of these adjacent genes (AUX/IAA related) and thus contributes to the reported phenotype. I understand the authors’ reluctances in carrying out full-scale transgenic cotton experiments, however, they need, at least, to analyze the levels of related plant hormones (not only IAA) during cotton fiber development in the selected cotton accessions, with or without the PAV.

*Response: Thanks very much for your comments. In the revised manuscript, we mainly measured the levels of plant hormones, including IAA, ABA, JA and JA-Ile, in representative cotton accessions with absence and presence variations. A total of 8 accessions with absence haplotype (in the GhImpCHN group) and 8 accessions with presence haplotype (4 from the GhImpUSO group and 4 from the GhImpCHN group) were used. We found that the content of IAA exhibited a generally lower level in accessions with the absence haplotype than those with the presence haplotype at both 8 DPA and 16 DPA cotton fibers (Figure R1). There was a slight difference in ABA content at 16 DPA fiber between two haplotypes. However, JA and JA-Ile levels did not show significant changes between absence and presence haplotypes. Actually, the content of plant hormones are regulated by many genes, and it is a challenge to identify the key genetic variation with a predominant role using only the current natural population which exhibits a large difference of genomic background between these accessions. Considering this, we are trying to knock out this presence variation using the CRISPR/Cas9 system. The comparison between CRISPR/Cas9 mutants and normal plants in the future may provide more evidence showing the putative role of this PAV in the regulation of auxin levels in cotton fibers. In the current manuscript, we only show that the PAV was accompanied by significant low expression of an adjacent gene encoding an AUX/IAA transcriptional regulator family protein. This result was shown in Supplementary Figure 30.*

Other concerns:

1. The authors listed 12 references in the “point-to-point response” file as the response to reviewers. They need to put these references in their manuscript accordingly.

*Response: Thank you for this suggestion. We confirmed that all the 12 references were cited in the manuscript.*

2. For the newly added 5 references in the revised manuscript (ref. #85-89), 4 of these cited references were from the authors’ lab. Again, the ref. #88 and 89 have the same author name and title, and also were not peer-reviewed. DELETE ALL OF THEM!

*Response: Thank you for this suggestion. Ref. #88 and #89 have been deleted in the revised manuscript.*

3. For the newly added Figure S6d, the authors did not describe how they normalized the level of gene expression. I did not understand why the expression values of KCS2 for both wild and cultivar were lower than 0!

*Response: Thank you for this suggestion. Here, gene expression level was calculated using Fragments Per Kilobase of exon model per Million mapped fragments (FPKM), and the formula of gene expression normalization is log2(FPKM+0.1). So, the normalized gene expression may be negative (KCS2 in cultivars with FPKM <1). We have added this normalization method to the figure legend of revised manuscript.*

4. For figure 6g, the legend color for ‘Presence’ is not correct.

*Response: Thanks very much for pointing out this mistake. The color for ‘Presence’ has been corrected in the revised manuscript.*

5. It’s not a good choice to delete the description of “for fiber elongation and environmental adaption”. I suggest the authors just rework the sentence and add related references.

*Response: Thank you for this suggestion. We have rewritten this sentence to “Further manipulation of these genes in plant hormone pathway and stress response pathway may help illustrate their putative regulatory role in fiber quality improvement and environmental adaptation during cotton domestication”.*

*Two new references were cited as below.*

*1. Huang G, Huang JQ, Chen XY, Zhu YX. Recent Advances and Future Perspectives in Cotton Research. Annu Rev Plant Biol. 2021;72:2.1-2.26.*

*2. Shi YH, Zhu SW, Mao XZ, Feng JX, Qin YM, et al. Transcriptome profiling, molecular biological, and physiological studies reveal a major role for ethylene in cotton fiber cell elongation. Plant Cell. 2006;18:651–64.*

6. For the newly added Figure S11a, the author should also check the DNA sequence in the edited region for the At-subgenome homologous gene, not only the target region in the Dt-subgenome.

*Response: Thanks very much for this suggestion. As you know, many homoeologous genes between At- and Dt subgenome in tetraploid cotton showed a very high sequence similarity. It is a challenge to knock out every specific gene, especially for many gene pairs with identical sequences. In the sgRNA design, we tried to use specific sequences in the At and Dt subgenome. We compared the sgRNA sequence in the Dt subgenome with homologous sequence in the At subgenome, and found that there was a SNP variation on sgRNA (c>g; Figure S11c). We amplified homologous PCR fragments of At- and Dt-subgenome to detect putative homologous editing. A total of 80 TA clones were used for Sanger sequencing. According to SNP variation, we found that both A and D homologous genes were edited. For Ghir_D05G013680, 1 bp insertion and 6 bp deletion were the main editing type; and 1 bp deletion was the main type for Ghir_A05G013930 (Figure S11c). Even though both genes were edited, the similarity of amino acid sequence between Ghir_D05G013680 and Ghir_A05G013930 was 97.8% (Figure R2), which suggests that they may have similar biology function and should not affect the functional study of this gene in fiber development.*

7. For the newly added Figure S19, the two gel pictures were too dirty and ugly. Please redo it with enough repeats.

*Response: Thanks very much for this suggestion. We performed the gel electrophoresis experiment again and the new gel pictures were shown.*

8. Line 486-488, according to the revised Table S1, there are 1,874 reported cotton accessions and 87 newly sequenced cotton accessions, not 1,872 and 89, respectively.

*Response: Thank you very much. We have modified the number of sequenced accessions in the revised manuscript.*

9. According to the point-to-point response, the authors did not analyze and combine the DUP, INV, TRA because the low frequency distribution in the population. It’s better to clarify this in the note in Table 1.

*Response: Thanks very much for this suggestion. We have clarified the description of structural variation in Table 1.*

10. The author need to keep all barbadense TRA accessions in Figure 1e. I suggest them to use two panels (G. hirsutum population and G. barbadense population) with different scales to display the data.

*Response: Thanks very much for your suggestion. We have shown the SV number of G. hirsutum panel (GhImpCHN, GhImpUSO, Ghlandrace) and G. barbadense panel (GbImpI, GbImpII, Outgroup) in the revised manuscript.*

11. No description for Figure S6b in the main text in this revised version. Delete this part of the figure or cite it in the text!

*Response: Thanks. The order of Figure S6 has been reorganized and Figure S6a-S6d has been put into the main text of revised manuscript.*

12. The authors deleted the FS and FM for the figure 6f in this revised version. They need to retain these parts and revising the description in the manuscript.

*Response: Thanks very much for your suggestion. The phenotype changes of FS and FM have been shown for accessions with the presence and absence haplotypes in Figure 6f. The description has also been revised.*
